# Organotin(IV) Alkoxides, Siloxides, and Related Stannoxanes. Characterisation and Thermogravimetric Studies

**DOI:** 10.1002/open.202400494

**Published:** 2025-02-04

**Authors:** Vlad Penciu, Liliana Bizo, Richard A. Varga, Adrian‐Alexandru Someşan

**Affiliations:** ^1^ Department of Chemistry Supramolecular Organic and Organometallic Chemistry Centre Faculty of Chemistry and Chemical Engineering Babeş-Bolyai University Cluj-Napoca RO-400028 Romania; ^2^ Department of Chemical Engineering Faculty of Chemistry and Chemical Engineering Babeş-Bolyai University 400028 Cluj-Napoca Romania

**Keywords:** Siloxide, Solid-state structures, Stannaboroxane, Thermogravimetric analysis, Tin

## Abstract

A series of *C,O*‐chelated organotin(IV) alkoxides, L_2_PhSnO^*t*^Bu (**4**), L_2_PhSnOMe (**6**), L_2_Sn(O^*t*^Bu)_2_ (**11**), and siloxides L_2_PhSnOSiPh_3_ (**3**), L_2_Sn(OSiPh_3_)_2_ (**10**) (L=[2‐(CH_2_O)_2_CH]C_6_H_4_), was prepared by salt elimination reactions. They were obtained from the organotin(IV) iodides L_2_PhSnI (**1**) or L_2_SnI_2_ (**2**) upon reactions with ^*t*^BuOK, MeONa or Ph_3_SiONa, respectively, in dry THF or methanol. Under non‐inert conditions, compounds **4** and **6** undergo combined hydrolysis and condensation to give the hexaorganodistannoxane (L_2_PhSn)_2_O (**5**). The stannoxane **5** is easily hydrolysed to L_2_PhSnOH (**7**), which quickly converts back when heated. Basic hydrolysis of diiodide **2** produces the cyclic oxide (L_2_SnO)_3_ (**8**). Its reaction with an equimolar amount of Ph_3_SiONa gives only a mixture of the expected L_2_SnI(OSiPh_3_) (**9**), **10** and the precursor, **2**. Yet, **8** shows a unique reactivity pattern when combine with *m*‐tolyl boronic acid, affording stannaboroxane (L_2_SnO)_2_OB(*m*‐tol) (**12**). All the isolated species were characterised in solution by NMR spectroscopy and mass spectrometry. The solid‐state molecular structures of **1**–**5**, **10**–**12** were established by single‐crystal X‐ray diffraction (XRD). Additionally, thermogravimetric analysis of **3**–**5**, **8**, **10**, and **12** was conducted.

## Graphical abstract

The pendant‐arm ligand [2‐(CH_2_O)_2_CH]C_6_H_4_ (L) was successfully employed in the isolation of a limited series of organotin(IV) alkoxides and siloxides. A unique example of stannaboroxane was synthesised following a straight‐forward protocol. The thermogravimetric studies performed on the novel organotin(IV) siloxides reveal their potential application as single‐source precursors for tin‐silicate materials.

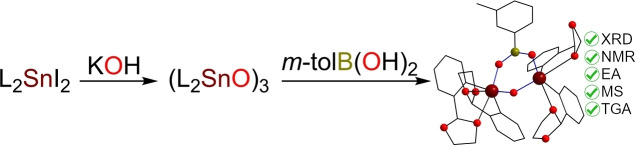

## Introduction

**Figure 1:**
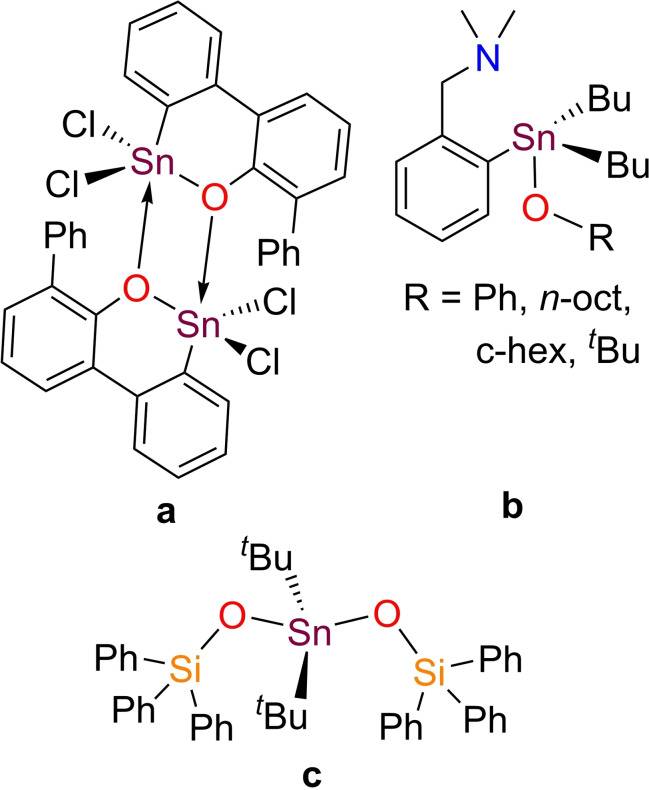
Selected examples of organotin(IV) alkoxides (a, b) and siloxides (c).

Metal alkoxides represent versatile species that are useful precursors for a wide variety of metal oxide materials,[^1^, ^2^] or inorganic‐organic hybrid materials.[^3^, ^4^] Although Davis and coworkers reported two alternative methods to prepare organotin(IV) alkoxides (*i. e*. reaction of bis[trialkyltin(IV)] oxides with dialkyl carbonates or with alcohols) more than 50 years ago,^[5]^ structurally characterised organotin(IV) alkoxides with general formula R_n_Sn(OR’)_4‐n_ (R, R’=alkyl, aryl; n=1–3) are scarce.[^6^, ^7^, ^8^, ^9^, ^10^] An illustrative example of intramolecular arene C−H bond activation was observed in the reaction of SnCl_4_ with 2 equiv. of LiOAr (OAr=2,6‐diphenylphenoxide) which gave a cyclometalated dimer (Figure 1a).^[11]^ Deacon showed the ability of Me_3_SnOAr (OAr=2,6‐di‐*tert*‐butyl‐4‐methylphenoxide) to transfer the aryloxide ligand to Sm or Yb *via* a redox transmetallation reaction in THF.^[12]^ Several diaryltin(IV) di‐*iso*‐propoxides were screened for their catalytic activity in ring‐opening polymerization of L–Lactide. The activity found was moderate.^[13]^ A significant contribution was made by Růžička and co‐workers who synthesised [2‐(Me_2_NCH_2_)C_6_H_4_]‐containing tin compounds (Figure 1b) and probed their reactivity towards CO_2_ or employed them in transesterification reactions.^[14]^ Yet, none of the isolated organotin(IV) alkoxides was structurally authenticated by single‐crystal XRD analysis.

Metal triorganosiloxides (M–OSiR_3_) are also suitable precursors for MSiO_x_ metal silica‐based materials.[^15^, ^16^, ^17^, ^18^, ^19^] However, the lack of molecular organotin(IV) siloxides is even more pronounced compared to alkoxides. Thus, only a few studies have reported fully characterised organotin(IV) siloxides up to date, while their reactivity potential was neglected.[^20^, ^21^, ^22^] A recent paper describes the synthesis, photocatalytic properties and potential application of some diaryltin(IV) siloxanes as single‐source precursors for tin‐silicate materials. Nevertheless, only dibuthyltin(IV) bis(triphenylsiloxide) (Figure 1c) was validated by single‐crystal XRD.^[23]^

Organotin(IV) oxides are also a significant class of tin compounds.[^24^, ^25^, ^26^] Their solid‐state structure ranges from polymeric, when small substituents are present on the metal centre,^[27]^ to trimeric[^28^, ^29^, ^30^] or even monomeric, as in hexaorganodistannoxanes (R_3_Sn)_2_O (R = Ph,^[31]^ *o*‐tol,^[32]^ *o*‐methoxyphenyl,^[33]^ etc.). Moreover, it was shown that the polarity of the crystallisation solvent can constrain the oligomerization rate of various stannoxanes.^[34]^

In recent years, the seeking for metallaboroxines [M(O_3_B_2_R_2_), M = Au,^[35]^ Sb,[^36^, ^37^] Bi,[^37^, ^38^, ^39^] Sn,[^36^, ^39^] Ga^[40]^] was extended. Thus, a variety of synthetic methods have been described, such as the reaction of organoboronic acids or boroxines^[41]^ with either organopnictogen(III) oxides,^[39]^ organotin(IV) carbonates,^[36]^ or organogallium(III) amides,^[40]^ or by arrested transmetallation reactions.^[35]^ Some of these molecular compounds have shown promising features, including luminescence^[35]^ or the utilisation as precursors for the deposition of thin film layers.^[40]^ Yet, there are still aspects to clarify regarding the bond situation and reactivity of all these species.

We have successfully employed various *C,O*‐chelating aromatic ligands in organotin(IV) chemistry as an alternative to the ubiquitous [2‐(Me_2_NCH_2_)C_6_H_4_]. Their oxygen‐containing pendant arms displayed more functionalisation possibilities, *i. e*. the aldehyde can be readily converted into a carboxylic acid or imine without affecting the C−Sn bond.[^42^, ^43^, ^44^, ^45^] The resulting species show great potential as organometalloligands able to generate heterometallic species containing Sn/Pd,^[43]^ Sn/Zn^[44]^ or Sn/Cu^[46]^ cores. We are currently exploring other potential applications for organotin(IV) species containing *C,O*‐chelating aromatic substituents. Thus, herein we report on the synthesis, spectroscopic characterisation, thermogravimetric studies, and the crystal structures of novel aryltin(IV) oxides, alkoxides and siloxides.

## Results and Discussion

### Synthesis

**Scheme 1:**
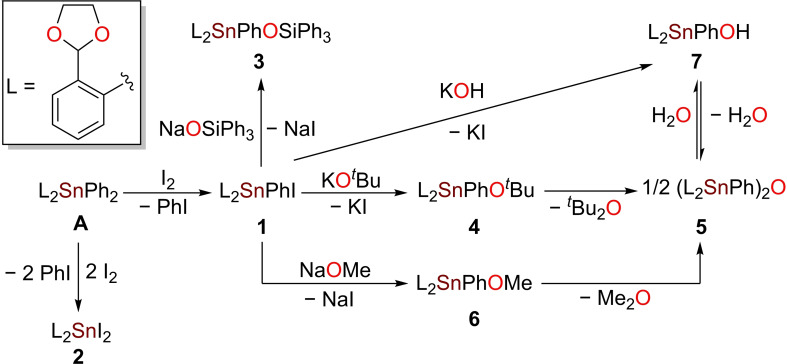
Synthesis of compounds **1**–**7**.

The reaction in dichloromethane of L_2_SnPh_2_ ^[47]^ (**A**) (L = [2‐(CH_2_O)_2_CH]C_6_H_4_) with one or two molar equivalents of elemental iodine afforded in high yields the organotin(IV) iodides L_2_PhSnI (**1**) and L_2_SnI_2_ (**2**), respectively, as pale‐yellow crystalline solids (Scheme 1). Treatment of **1** with a THF solution of NaOSiPh_3_ ^[48]^ using Schlenk techniques gave the desired L_2_PhSnOSiPh_3_ (**3**), a rare occurrence of structurally characterised organotin(IV) siloxide, prepared through the salt metathesis reaction.[^20^, ^22^] The siloxide **3** is an air and moisture stable solid which is inert towards reaction with CO_2_. The alkoxides L_2_PhSnO^*t*^Bu (**4**) and L_2_PhSnOMe (**6**) were synthesised in a similar manner by reaction of **1** with a THF solution of ^*t*^BuOK or a methanolic solution of freshly prepared sodium methoxide. The moisture‐sensitive **4** forms in yields over 70 % in 30 minutes, when a commercially available 1 M THF solution of ^*t*^BuOK is used. Yet, the utilisation of solid ^*t*^BuOK only affords traces of **4**, while the main product is (L_2_PhSn)_2_O (**5**) as authenticated by NMR and XRD analysis (Figure S43). Although **6** was isolated as a white crystalline solid, the decomposition product **5**, was also detected in solution by ^1^H and ^119^Sn{^1^H} NMR spectroscopy. The amount of stannoxane **5** is about 14 %. On the other hand, **5** is quantitatively hydrolysed to give L_2_PhSnOH (**7**), which rapidly converts back upon heating. Basic hydrolysis of **1** in a biphasic CH_2_Cl_2_/water system is an alternative pathway to **7**.

The cyclotristannoxane (L_2_SnO)_3_ (**8**) was isolated almost quantitatively as colourless sharp melting solid upon reaction of **2** with aqueous KOH in THF.

**Scheme 2:**
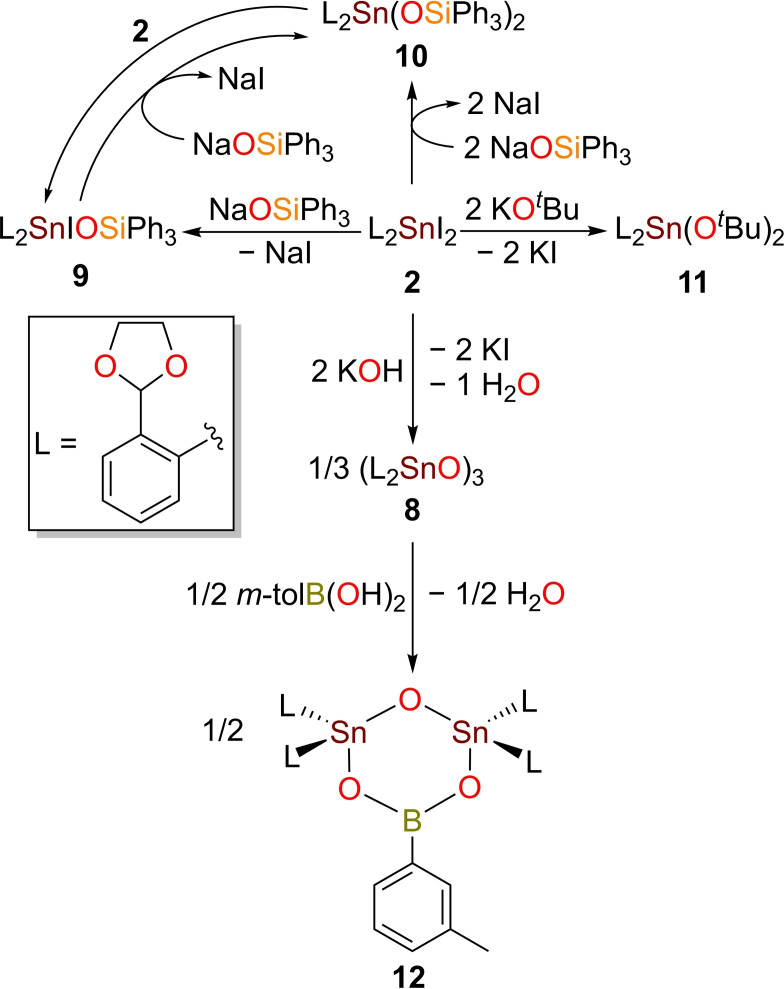
Synthesis of compounds **8**–**12**.

Treatment of **2** with two equivalents of Ph_3_SiONa in dry THF under inert atmosphere effected its quantitative conversion to diaryltin(IV) bis(triphenylsiloxide) L_2_Sn(OSiPh_3_)_2_ (**10**) (Scheme 2). Compound **10** was isolated as an air stable colourless solid. A mixture of L_2_SnIOSiPh_3_ (**9**), **10** and **2** was detected in solution by NMR spectroscopy, when **2** reacted with only one equiv. of Ph_3_SiONa at −78 °C in THF. The same outcome was observed by the reaction of **2** with **10** in a 1 : 1 molar ratio, showing there is an equilibrium in solution between these 3 species. Compound **9** was only detected in solution by ^1^H and ^119^Sn{^1^H} NMR spectroscopy (*δ*_119Sn_=−297.9 ppm) (Figure S36).

The diorganotin(IV) dialkoxide L_2_Sn(O^*t*^Bu)_2_ (**11**) was prepared upon reacting **2** with two equivalents of ^*t*^BuOK in THF, for 30 minutes. It shows good solubility in acetonitrile, THF, and in aromatic hydrocarbons and is less prone to hydrolysis compared to **6**. When stannoxane **8** is heated at reflux in toluene with *m*‐tolyl boronic acid in a 2 : 3 molar ratio, the stannaboroxane (L_2_SnO)_2_OB(*m*‐tol) (**12**) is obtained as a single product.

The new species exhibit good solubility in THF and/or in chlorinated (CH_2_Cl_2_, CHCl_3_) solvents.

### Spectroscopic Characterisation

All the isolated compounds were fully characterised in solution by ^1^H, ^13^C, ^119^Sn and ^29^Si NMR where appropriate, in CDCl_3_ and/or C_6_D_6_ at room temperature. Except for compound **9** being involved in an equilibrium with different species, the ^1^H and ^13^C{^1^H} NMR spectra each display one set of characteristic resonance signals for the organic substituents bound to the tin centre. The ^1^H NMR spectra of **1** and **2** exhibit similar features, with the most deshielded resonances corresponding to *H*‐6 (for the numbering scheme, see Figure 11). The two iodide substituents attached to tin in **2** increase the ^3^ *J*_Sn‐H_ coupling constant of *H*‐6 resonance, from 75 Hz, found for the iodide **1**, to 111 Hz. The C*H*_2_ protons from the 1–3 dioxolane rings in **1** give rise to a multiplet centred at 3.6 ppm, whereas the same protons appear as a broad resonance (*δ*=3.7 ppm) for **2**. The difference was also noticed in ^13^C{^1^H} NMR spectra, where only one resonance was observed for C8−C9 in **2** (*δ*=65.10 ppm), which contrasts with two distinct resonances (*δ*=64.65, 64.96 ppm) corresponding to the same carbon atoms in **1**. This indicates a strong intramolecular O→Sn interaction in solution of **1**, which alters the symmetry of the dioxolane rings. This feature is also consistent with different strength of Sn−O interactions observed in the solid‐state structure of **1** (*vide infra*). The same behaviour perpetuates to derivatives **3**–**7** (Figures S7–S20). Besides, on the NMR time scale, there is a faster fluctional behaviour involving coordination/decoordination of both *C,O*‐pendant‐arm ligands of **2** in CDCl_3_, implied by the broad resonance in ^1^H NMR and only one resonance for C8‐C9 in ^13^C{^1^H} NMR. The formation of the siloxide **3** was easily recognizable by ^1^H NMR, where the singlet resonance corresponding to *H*‐7 shifted from 5.85 ppm (**1**) to 5.64 ppm. The ^29^Si NMR for **3** completed its characterisation in solution with a singlet at −22.3 ppm. Indeed, the resonance for *H*‐7 in ^1^H NMR spectra is also indicative for the formation of the alkoxides **4** and **6** [*δ*_1H_=5.77 ppm (**4**); 5.69 ppm (**6**)], the oxide **5** (*δ*_1H_=5.75 ppm), or the hydroxide **7** (*δ*_1H_=5.77 ppm). Each of the ^1^H NMR spectrum of *tert*‐butoxides **4** and **11** in C_6_D_6_ exhibits a singlet [*δ*_1H_=1.45 ppm (**4**), 1.52 ppm (**11**)] as expected, while the presence of the methoxy substituent in **6** is confirmed by a sharp resonance (*δ*_1H_=4.00 ppm), surrounded by tin satellites (^3^ *J*_Sn‐H_=38 Hz). A sharp singlet was also detected in the ^1^H NMR spectrum of **7**, where the ‐O*H* proton shows at *δ*_1H_=0.69 ppm (^2^ *J*_Sn‐H_=24 Hz). This is consistent with solution NMR data found for similar organotin(IV) hydroxides reported in the literature.^[14]^

**Figure 2:**
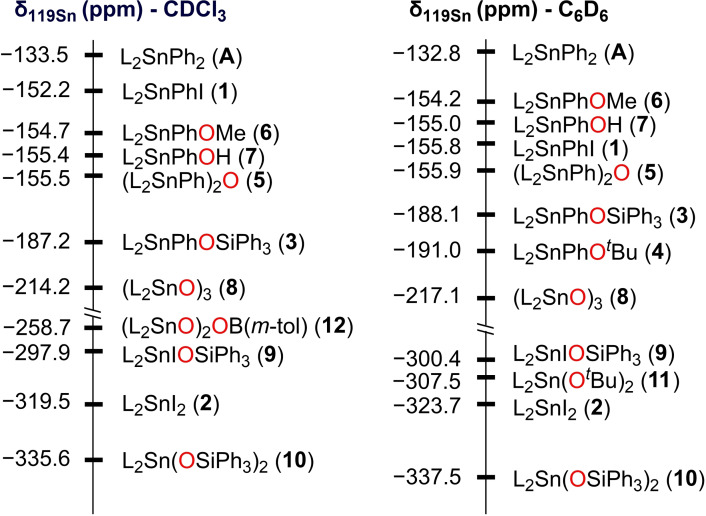
^119^Sn NMR chemical shifts for compounds **1**–**12** and **A**.

The ^119^Sn{^1^H} NMR spectroscopy was an indispensable tool that consolidated our findings in solution and helped in the monitoring of the reactions. Thus, ^119^Sn NMR spectra have been recorded in both C_6_D_6_ and/or CDCl_3_ and the resulted chemical shifts are summarized in Figure 2. The triorganotin(IV) compounds **1**, **3**–**7** display typical ^119^Sn NMR chemical shifts (range between −154.3 to −191.0 ppm in C_6_D_6_) which are indicative for pentacoordinated triaryltin(IV) species in solution showing only one O→Sn intramolecular interaction. These findings fit well those from a series of compounds containing the L^1^ = [2‐(Me_2_NCH_2_)C_6_H_4_] moiety, *i. e*.: L^1^Ph_2_SnX (*δ*_119Sn_ (ppm)=−199.5, X = I;^[49]^ −187.6, X = OH; −173.2, X = OSnPh_2_L^1^)^[14]^ and with the phosphine‐based derivative, [*o*‐(Ph_2_P)C_6_H_4_]_3_SnI, *δ*_119Sn_=−177.2 ppm.^[50]^ The ^119^Sn NMR resonance found for **3** is significantly upfield shifted compared to that reported for (Ph_3_SnO)Ph_2_SiOSiPh_2_(OSnPh_3_) (−106.5 ppm),^[22]^ suggesting the presence of intramolecular O→Sn contacts in solution. The diorganotin diiodide **2** and the corresponding disiloxide derivative **10** exhibit ^119^Sn NMR resonances at −319.5 ppm and −335.6 ppm, substantially shifted related to that in **12** (−258.7 ppm), Ph_2_SnI_2_ (−243.8 ppm)^[51]^ or (*o*‐An)_2_SnI_2_ (−287.3 ppm)^[52]^ and in the same region as those containing the *C,N*‐pendant arm ligand, L^1^, *e. g*. L^1^_2_SnI_2_ (−346.9 ppm)^[49]^ and L^1^PhSnI_2_ (−337.4 ppm).^[53]^

In the ESI+ or APCI+HRMS spectra of **1**–**3** and **5** the base peaks correspond to [M−X]^+^ fragments, where X = I (**1**, **2**), OSiPh_3_ (**3**) and OSnPhL_2_ (**5**). Relevant fragments were also detected for **8** *m/z*=435.02344 [L_2_SnOH]^+^ and **10** *m/z*=693.10999 [L_2_SnOSiPh_3_]^+^ that validate the fragmentation pattern for the isolated species (see Figures S52–58, for details). The molecular ion [M+H]^+^ of the stannaboroxane **12** was also observed (*m/z*=985.10458).

### Solid‐State Structures

Despite the well‐known di‐ and triaryltin chlorides, there are only several examples of XRD structurally authenticated aryltin(IV) iodides and diiodides that can be found in CCDC.

Suitable crystals for XRD analysis of the triorganotin iodide **1** and diorganotin diiodide **2** were grown by slow diffusion of pentane into concentrated CH_2_Cl_2_ solutions of the corresponding compound. Compound **1** crystallised in the monoclinic space group *P*21/c with two pairs of crystallographic independent molecules in the unit cell (Figure S37–S39).

**Figure 3:**
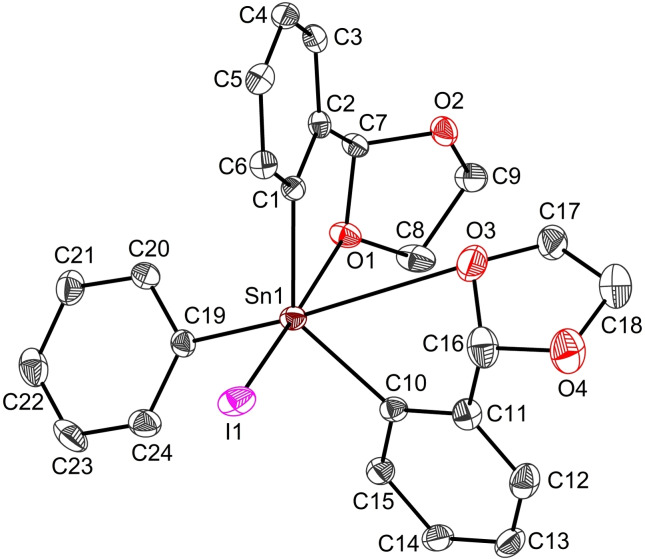
View of the molecular structure of L_2_PhSnI (**1**) with ellipsoids at the 50 % probability level. Only one, *S*_C7_‐*S*_C16_‐**1 a**, of the two enantiomers in the asymmetric unit is shown.

The molecular structure of *S*_C7_‐*S*_C16_‐**1 a** isomer (Figure 3) features a hexacoordinated tin centre, in a distorted octahedral arrangement, with the O1−Sn1−I1 angle of 174.35(6)°, slightly smaller than the one found in *S*_C31_‐*S*_C40_‐**1 b** [O5−Sn2−I2 175.72(5)°]. In addition, the coordination geometry in **1 a** displays a higher distortion from the ideal octahedral one, with O3−Sn1−C19 angle of 164.99(1)° substantially altered with respect to its correspondent angle in **1 b** [O7−Sn2−C43 178.37(1)°]. The two O→Sn intramolecular contacts in **1 a** [O1→Sn1 2.430(2) Å, O3→Sn1 3.120(3) Å] are also different than those observed in **1 b** [O5→Sn2 2.492(2) Å, O7→Sn2 2.847(2) Å] (Table 1, Table S3). These magnitudes are commensurate with those found for other hexacoordinated triaryltin(IV) halides containing *C,O*‐bidentate ligands, *e. g*. L_3_SnI [2.598(4)/2.849(5) Å],^[54]^ and [2‐(O=CH)C_6_H_4_]_2_PhSnCl [2.444(2)/2.931(2) Å]^[47]^ or *O,C,O* pincer‐type ligands, *e. g*. [2,6‐(ROCH_2_)_2_C_6_H_3_]SnPh_2_X [2.586(2)/2.891(2) Å; R = Me, X = I;^[55]^ 2.567(2)/2.993(2) Å; R = Me X = Cl].^[56]^

**Table 1:** Selected interatomic distances (Å) and bond angles (°) for compounds **1**, **3**, **4** and **10**.

	**1**	**3**	**4**	**10**
Sn1−C1	2.134(3)	2.138(4)	2.130(2)	2.126(4)
Sn1−C10	2.144(3)	2.140(3)	2.145(2)	2.119(4)
Sn1−X^[a]^	2.817(3)	2.005(2)	1.9997(1)	1.996(3)
Sn1−Y^[b]^	2.147(3)	2.139(4)	2.135(2)	1.991(3)
Sn1−O1	2.430(2)	2.563(2)	2.706(2)	2.492(3)
Sn1−O3	3.120(3)	2.966(2)	3.713(1)	2.567(3)
O1−Sn1−X	174.35(6)	168.27(7)	163.89(5)	171.97(11)
O3−Sn1−C19	164.99(1)	171.34(11)	151.82(5)	165.60(11)
C1−Sn1−C10	132.36(13)	126.48(14)	121.86(6)	148.11(18)
C1−Sn1−C19	112.70(13)	114.32(12)	112.12(6)	104.79(14)
C1−Sn1−O1	74.21(10)	71.66(11)	70.07(5)	73.21(14)
C1−Sn1−O3	68.27(12)	67.61(11)	63.37(5)	80.38(13)
C1−Sn1−X	92.50(9)	96.74(14)	93.83(6)	99.25(15)

^[a]^X=I1 for **1**, O5 for **3**, **4** and **10**. ^[b]^Y=C19 for **1**, **3** and **4**, O6 for **10**.

Consequently, two five‐membered SnC_3_O rings are formed in **1**, and the methylene carbon atoms (C7, C16) become chiral centres, giving a racemate of two enantiomers for each different molecule in the crystal (see Figure S38 and S39 for details).

**Figure 4:**
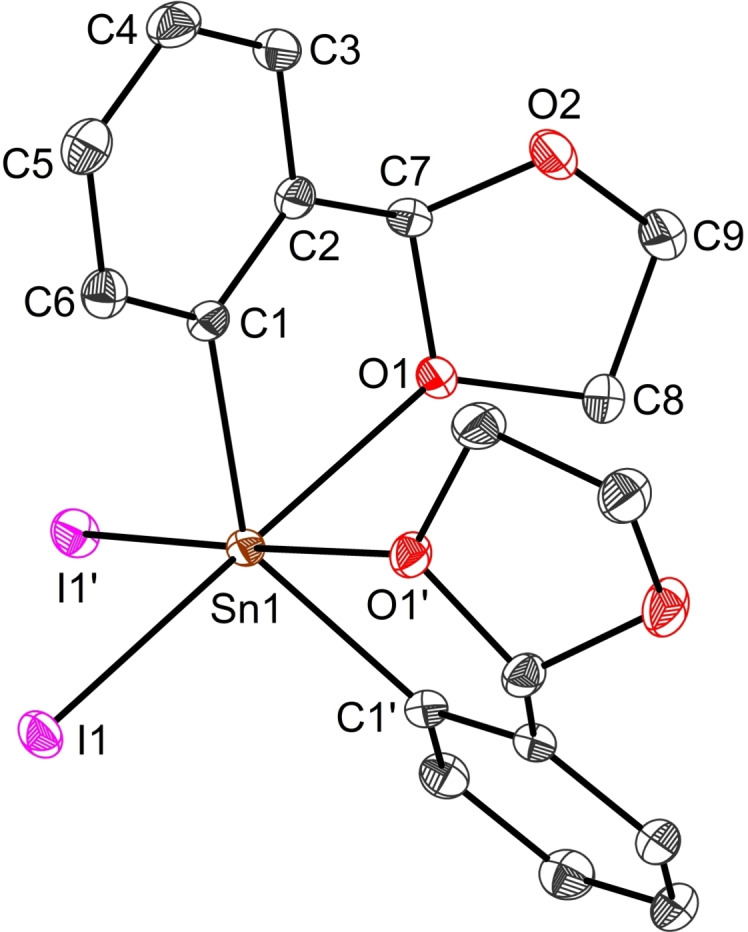
View of the molecular structure of L_2_SnI_2_ (**2**) with ellipsoids at the 50 % probability level. Only one, *S*_C7_‐*S*_C16_‐**2** of the two enantiomers is shown. Selected distances (Å) and angles (°): Sn1−C1 2.1308(18), Sn1−I1 2.7777(17), Sn1−O1 2.4903(13); O1−Sn1−I1 176.86(3), C1−Sn1−I1’ 100.23(5), C1−Sn1−O1’ 79.62, C1−Sn1−O1 73.37(6), C1−Sn1−I1 105.25(5).

Compound **2** crystalises in the centrosymmetric monoclinic space group C2/c. Its molecular structure shows the tin(IV) atom to lie in a six‐coordinate environment adopting a distorted octahedral geometry with a *cis*‐arrangement of the two iodide substituents (Figure 4). Both aryl ligands act in a bidentate fashion, with O1 strongly coordinated to Sn [2.4903(13) Å]. This value matches those observed in related *cis*‐configured diaryltin(IV) dihalides L_2_SnCl_2_ [2.500(5)/2.475(5) Å], [2‐(O=CH)C_6_H_4_]_2_SnCl_2_ [2.431(7)/2.480(7) Å],^[43]^ [2‐(MeOCH_2_)C_6_H_4_]_2_SnBr_2_ [2.419(4)/ 2.499(4) Å],^[57]^ and is smaller than the dative bonds found in [2,6‐(^*t*^BuOCH_2_)_2_C_6_H_3_]PhSnI_2_ [2.843(3)/2.789(3) Å].^[58]^ Yet, the O1→Sn1 contact in **2** is considerably weaker than those in the *trans*‐configured P=O→Sn coordinated diorganotin(IV) dihalide (L^P=O^)PhSnCl_2_ [L^P=O^={C_6_H_2_[P(O)(OEt)_2_]_2_–2,6‐^*t*^Bu‐4}^−^] [Sn1−O1 2.278(6) Å, Sn1−O2 2.203(5) Å].^[59]^

**Figure 5:**
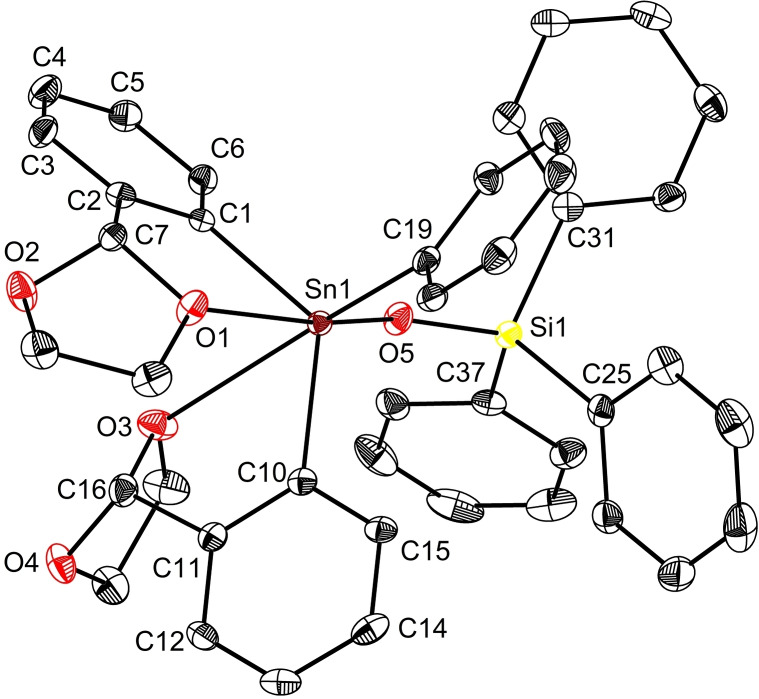
View of the molecular structure of L_2_PhSnOSiPh_3_ (**3**) with ellipsoids at the 50 % probability level. Only one, *S*_C7_‐*R*_C16_‐**3**, of the two enantiomers in the asymmetric unit is shown.

The siloxide **3** crystalised upon slow diffusion of hexane into an Et_2_O solution of the compound. Crystal contains a racemic mixture of *S*_C7_‐*R*_C16_‐**3** (Figure 5) and *R*_C7_‐*S*_C16_‐**3** (Figure S41). The O→Sn intramolecular interactions in **3** lie between the sum of the covalent radii [Σr_cov_(Sn,O) 2.05 Å]^[60]^ and the sum of the vdW radii [Σr_vdW_(Sn,O) 3.92 Å]^[61]^ of the elements, contributing to a distorted octahedral arrangement around tin with 3 bond angles close to 180° (Table 1). The Sn1−O5 bond [2.005(2) Å] is slightly longer compared to those in other reported organotin(IV) siloxides with a four‐coordinate tin centre [1.87(1) Å in Ph_3_SnOSiPh_3_,^[20]^ 1.93(2)/1.96(1) Å in (Ph_3_SnO)Ph_2_SiOSiPh_2_(OSnPh_3_)^[22]^], but commensurable with those in six‐coordinate tin containing siloxides.[^62^, ^63^]

**Figure 6:**
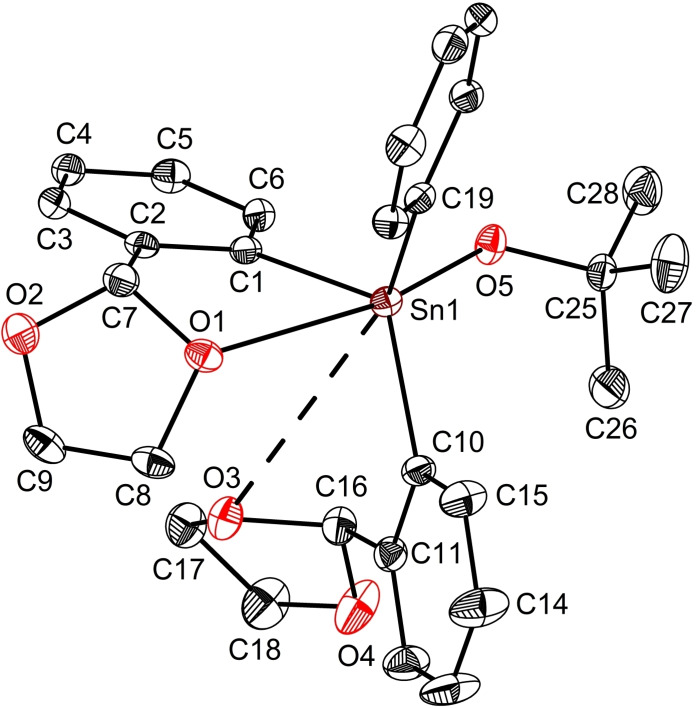
View of the molecular structure of L_2_PhSnO^*t*^Bu (**4**) with ellipsoids at the 50 % probability level. Only one, *S*_C7_‐*S*_C16_‐**4**, of the two enantiomers in the asymmetric unit is shown.

Compound **4** is a very rare example of non‐solvated aryltin(IV) alkoxide authenticated by single‐crystal XRD (Figure 6).[^13^, ^64^] Its solid‐state structure reveals a six‐coordinate tin atom with a loose O3→Sn1 contact of 3.713 Å, [Σr_vdW_(Sn,O) 3.92 Å]. Thus, the geometry about the tin centre in **4** could rather be described as a distorted capped trigonal bipyramid (τ_5_=0.70).^[65]^ The angle between the axial atoms, O1−Sn1−O5 is 163.89(5)°, while the equatorial site angles range between 112.12(7)° and 121.86(6)°, close to the ideal value of 120°. The Sn1−O5 bond length in **4** [1.9997(1) Å] resembles those observed in the few reported triaryltin(IV) alkoxides, Ph_3_SnOCMe_2_‐C(O)OEt [1.996(1) Å],^[64]^ (*p*‐Me_2_NC_6_H_4_)_3_SnO^*i*^Pr [2.0007(15) Å],^[13]^ and that from the siloxide **3** [2.005(2) Å]. The electronic density brought by the *tert*‐butoxy fragment clearly decreases the Lewis acidity of the tin centre in **4**. Accordingly, the coordination of O1 and O3 atoms to tin is weakened with respect to that in **1** or **3** (*vide supra*). The five‐membered ring SnC_3_O1 in **4** is folded along the Sn⋅⋅⋅C7 axis [dihedral angle SnC_3_/SnCO of 31.32°] with O1 atom deviated −0.58 Å out of the best SnC_3_ plane. This particularity contrasts the data disclosed for the molecular structures of **1** and **3**, where the chelating rings, generated by O1→Sn coordination, are almost planar [dihedral angles: 5.65° in **1**; 3.74° in **3**, with O1 out of the best SnC_3_ plane with 0.08 Å/−0.06 Å respectively].

**Figure 7:**
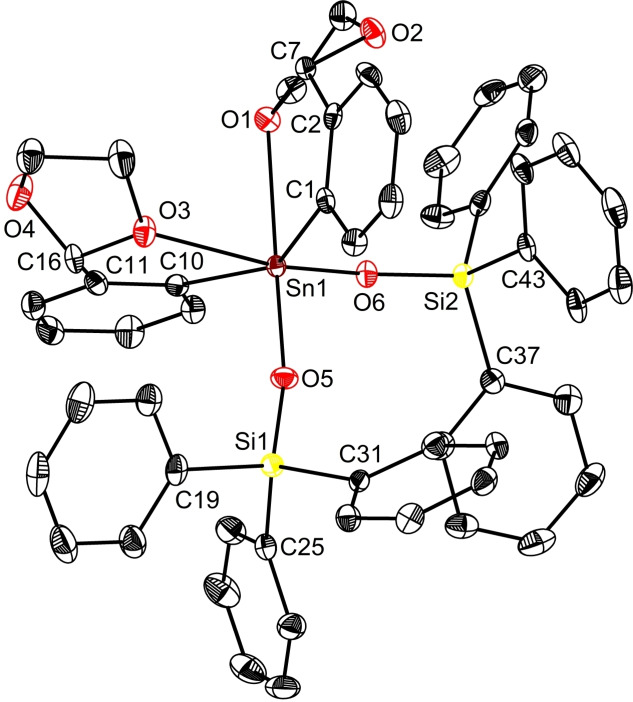
View of the molecular structure of L_2_Sn(OSiPh_3_)_2_ (**10**) with ellipsoids at the 50 % probability level. Only one, *S*_C7_‐*S*_C16_‐**10**, of the two molecules in the asymmetric unit is shown.

The asymmetric unit of the disiloxide, L_2_Sn(OSiPh_3_)_2_ (**10**) contains two independent molecules, with one depicted in Figure 7. Both pendant‐arm aromatic ligands are strongly coordinated to tin through O1 and O3 respectively, [2.492(3)/2.567(3) Å] giving a distorted octahedral geometry about the metal centre. The *cis* arrangement of the bulky Ph_3_SiO^−^ fragments on tin, alters substantially the Sn−O−Si angles. Accordingly, the Sn1−O5−Si1 and Sn1−O6−Si2 angles [168.41(2)/149.68(2)°] are wider relative to that found in **3** [144.7(2)°], but comparable with those observed in (^*t*^Bu)_2_Sn(OSiPh_3_)_2_ 163.84(1)°/149.75(1)°.^[23]^ Additional crystallographic data (including those for the second independent molecule of **10**) are listed in Table S5. On the whole, compound **10** is only the third representative of the family of diorganotin(IV) disiloxides,[^23^, ^66^] and the sole example with aromatic ligands.

**Figure 8:**
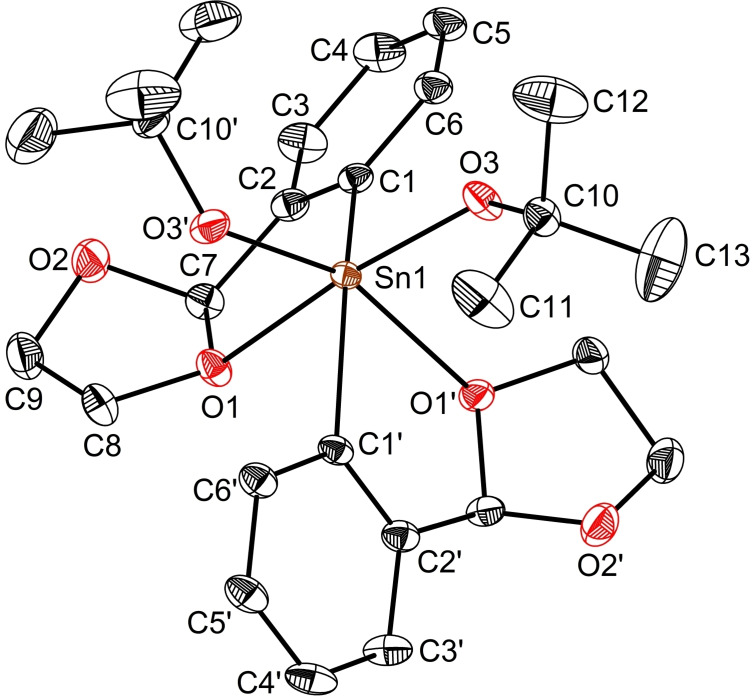
View of the molecular structure of L_2_Sn(O^*t*^Bu)_2_ (**11**) with ellipsoids at the 50 % probability level. Only one, *R*_C7_‐*R*_C7’_‐**11**, of the two enantiomers is shown [Symmetry equivalents atoms (*3/2–x, 3/2–y, z*) are given by ‘].

Single crystals of **11** suitable for XRD were grown by cooling a concentrated acetonitrile solution to −24 °C. Compound **11** is a rare case of structurally characterised organotin(IV) dialkoxide[^13^, ^67^] and the sole mononuclear one. Its molecular structure (Figure 8) shows the tin centre to rest in a six‐coordinate environment featuring a distorted octahedral geometry. The Sn1−O3 distance in **11** [1.991(1) Å] match those in **10** [Sn1−O5 1.996(2) Å, Sn1−O6 1.991(3) Å]. Yet, the O3−Sn1−O3’ angle is wider [107.17(8)°] compared to O5−Sn1−O6 angle in **10** [97.67(1)°], showing a higher distortion of the octahedral core (Table S6).

**Figure 9:**
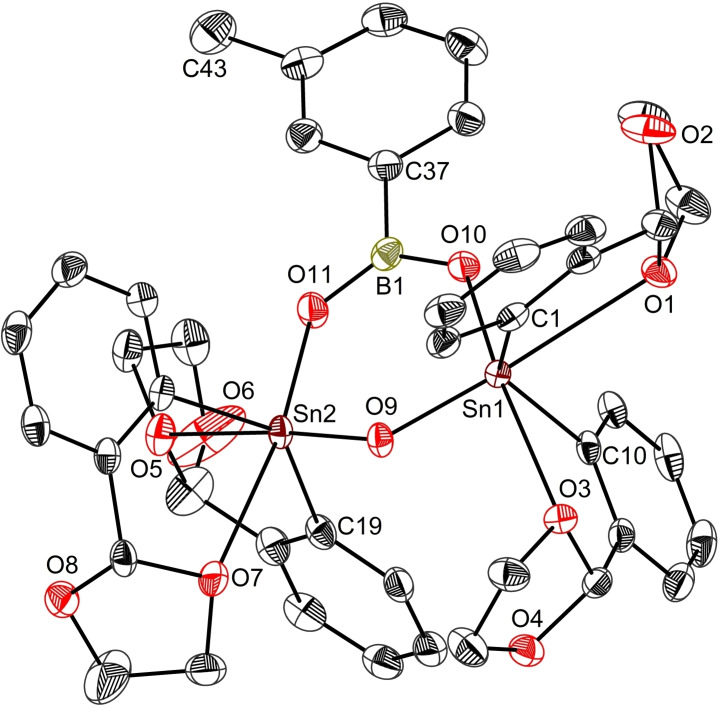
View of the molecular structure of **12** with ellipsoids at the 50 % probability level. Only one, *S*_C7_ *‐S*_C16_ *‐S*_C25_ *‐R*_C34_ *–* **12**, of the two enantiomers in the asymmetric unit is shown.

Single crystals of the stannaboroxane **12** grown by slow diffusion of hexane into a concentrated dichloromethane solution. Its unique molecular structure reveals two symmetric tin atoms, comprised in a non‐planar six‐membered heterocycle (Figure 9). The O9 atom is out −0.396 Å of the Sn_2_BO_2_ plane. Four strong intramolecular O→Sn interactions [O1−Sn1 2.781(2) Å, O3−Sn1 2.624(1) Å, O5−Sn2 2.604(2) Å, O7−Sn2 2.635(2) Å], that satisfy the electronic demands of the two tin atoms, prevent the addition of the intermediate, L_2_Sn(OH)_2_, to the six‐membered ring, contrasting the structure reported by Molloy, where a ^*t*^Bu_2_Sn(OH)_2_ unit wraps up the molecular structure of the stannaboroxane.^[68]^ As a result, both metal centres are hexacoordinated, displaying a distorted octahedral environment (for crystallographic data, see Table S7; details about the reaction pathway for the formation of **12** are given in Figure S50).

### Thermogravimetric Analysis (TG)

Thermal degradation of the alkoxide **4**, oxides **5** and **8**, siloxides **3**, **10**, and stannaboroxane **12** was monitored by thermogravimetry. The most interesting features were observed for the organotin(IV) siloxides.

**Figure 10:**
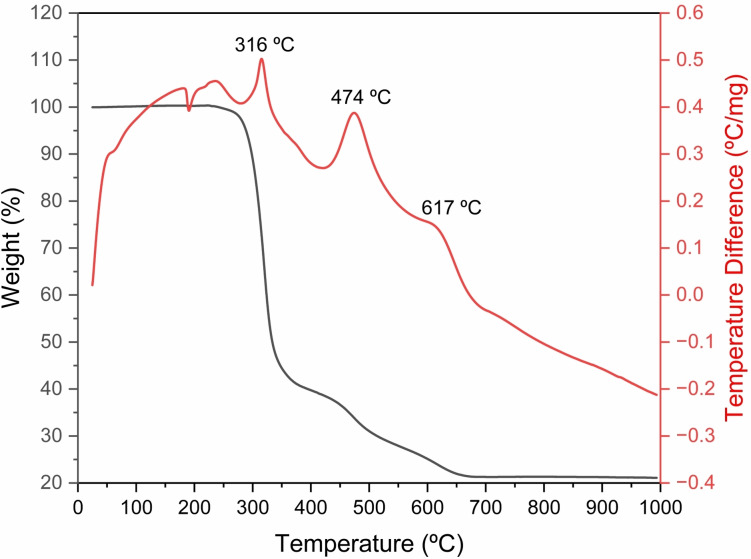
Thermogravimetry (TG ‐ black) and differential thermal analysis (DTA ‐ red) curves of **10** (exo up).

Thus, thermal behaviour of **10** is presented in Figure 10, where the obtained TG and DTA curves are displayed. The curves present variations in thermal stability among the sample. Compound **10** exhibits a three‐step decomposition during TG analysis (three exothermic peaks on DTA curve). The total weight loss of 78.63 % is close to the theoretically calculated weight loss due to organic moieties of the sample (78.22 %), resulting in 21.37 % SnO_2_.SiO_2_ ceramic.

Compound **3** exhibits a thermal behaviour with a two‐step decomposition visible on the TG/DTA curve. (Figure S59) The DTA curve shows two exothermic peaks at 310 °C and 474 °C resulting in an overall weight loss of 73.13 % (calculated 72.60 %). As previously, this total weight loss is close the theoretical value, thus resulting in 26.87 % residue, which could be attributed to SnO_2_.SiO_2_ ceramic (calculated 27.39 %).

The alkoxide **4** and the oxides **5** and **8** displayed similar two‐step decomposition patterns (Figure S60–S62). However, the residual mass found for these species (Table S8) does not resemble with any kind of tin oxide material, thus, for the complete identification of their decomposition products, additional investigations are required. A peculiar thermal behaviour was also observed for the stannaboroxane **12**. The TGA curve of **12** (Figure S63) displays a multistep decomposition pattern that progresses even at elevated temperatures (>1000 °C). This shows a complete decomposition of the material indicating sublimation of the title compound.

## Conclusions

The synthesis and complete characterisation of some organotin(IV) alkoxides, siloxides, stannoxanes and a unique stannaboroxane were presented. Both triorganotin(IV) alkoxides **4** and **6** show moisture sensitivity, their hydrolysis giving organotin(IV) oxide (L_2_PhSn)_2_O (**5**). The diorganotin dialkoxide **11** is more stable when exposed to air, while the corresponding siloxides **3** and **10** are inert towards oxygen or CO_2_. The two intramolecular O→Sn interactions, which vary in strength depending on the substituents, are a defining feature shared by all these derivatives. A *cis*‐arrangement of the substituents was observed in the solid‐state structures of the diorganotin(IV) compounds **2**, **10**–**12**. Both organotin(IV) siloxides display typical decomposition patterns, producing a tin silicate material residue. Most importantly, we achieved the preparation of a unprecedented stannaboroxane (**12**) following a facile high‐yield protocol that starts from the cyclic oxide (L_2_SnO)_3_ (**8**). An attempt at isolating a mono‐substituted diorganotin(IV) siloxide, **9**, failed under the experimental conditions employed but instead showed an equilibrium between the starting materials and the expected product. We are now further exploring the potential of different siloxides to be used as tin‐silicate precursors and will report on these findings in a forthcoming report.

## Experimental Section

### Materials and Procedures

All air‐ and moisture‐sensitive reactions were carried out under argon atmosphere, using standard Schlenk techniques or in a dry glovebox (Jacomex: O_2_ <1 ppm, H_2_O <1 ppm) for reagents loading. THF was distilled under argon from K prior to use. Starting materials: L_2_SnPh_2_ ^[47]^ and Ph_3_SiONa^[48]^ were prepared according to literature procedures. Commercially available products such as I_2_, Ph_3_SiOH, ^*t*^BuOK, KOH and Na were purchased from Fluorochem or Sigma and used as received. Deuterated benzene (Deutero, Germany) was stored in sealed ampoule over activated 4 Å molecular sieves and degassed by a minimum of three freeze–thaw cycles.

NMR spectra were recorded at room temperature on Bruker Avance III 400 and 600 NMR spectrometers. All chemical shifts are reported in *δ* units (ppm) relative to the residual signal of the deuterated solvent. (ref. CDCl_3_: ^1^H 7.26 ppm, ^13^C 77.16 ppm; ref. C_6_D_6_: ^1^H 7.16 ppm, ^13^C 128.06 ppm). Assignment of the signals was conducted using 1D (^1^H, ^13^C{^1^H}) and 2D (COSY, HMBC, HSQC) NMR experiments. (for the numbering schemes, see Figure 11; for ^1^H, ^13^C, and ^119^Sn NMR spectra, see Figures S1–S36). The chemical shifts for the ^119^Sn NMR spectra are reported relative to SnMe_4_ as external standard. The NMR spectra were processed using MestReNova software.^[69]^

**Figure 11:**
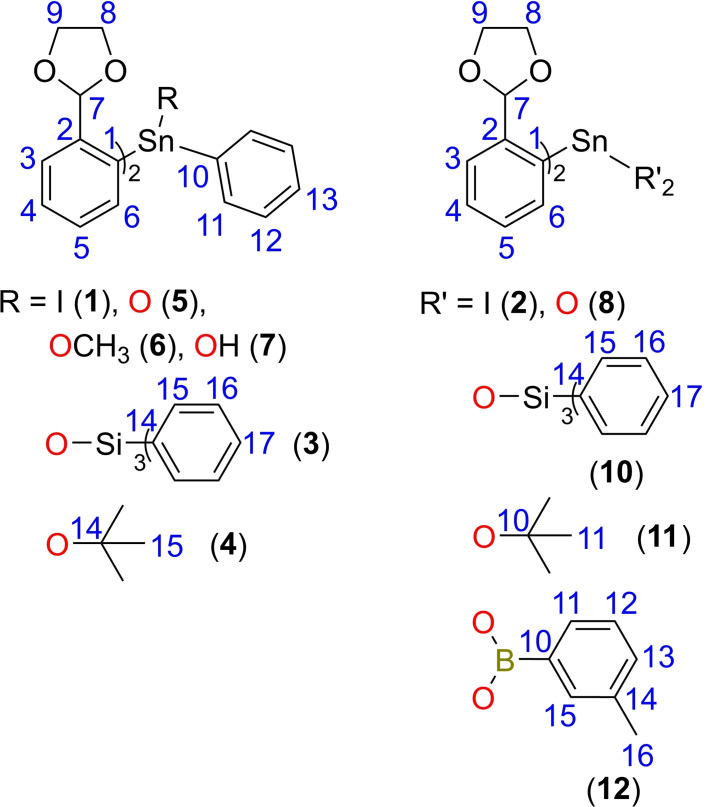
General NMR numbering scheme for compounds **1**–**8** and **10**.

Mass spectra were recorded on a Thermo Scientific LTQ Orbitrap XL mass spectrometer equipped with a standard ESI/APCI source. Data analysis and calculations of the theoretical isotopic patterns were carried out with the Xcalibur software package.^[70]^ Melting points were measured with an Electrothermal 9200 apparatus and are not corrected. Elemental analyses were carried out on a Flash EA 1112 analyser. Infrared spectra were recorded on a JASCO FT/IR‐615 instrument.

Thermal analyses were conducted on an SDT Q600 (USA) device from T.A. Instruments. Data on thermogravimetry (TG) and differential thermal analysis (DTA) curves, were simultaneously acquired under the following measurement conditions: heating from laboratory temperature to 1000 °C, at a heating rate of 10 °C/min, under normal air atmosphere, using alumina crucibles. For each measurement ~5 mg of material was used.

### Crystal Structure Determination

The details of the crystal structure determination and refinement for compounds **1**–**5**, **10**–**12** are given in Tables S1 and S2, respectively (see ESI†). The crystals were collected on a Bruker D8 VENTURE diffractometer using Mo−Kα radiation (λ=0.71073 Å) from a IμS 3.0 microfocus source with multilayer optics, at low temperature (100 K). The structures were refined with anisotropic thermal parameters for non‐H atoms. Hydrogen atoms were placed in fixed, idealized positions and refined with a riding model and a mutual isotropic thermal parameter. For structure solving and refinement the Bruker APEX5 Software Package was used.^[71]^ Visual representations were created with the Diamond program.^[72]^ The C41 and C42 carbon atoms from one of the 1,3‐dioxolan‐2‐yl rings of compound **1** are disordered over two positions and were modelled with site occupancies of 58 : 42. For compound **10** the measured crystal proved to be a two‐component twin [twin ratio: 80 : 20], arising from a rotation of −179.97° around the vector normal to (−1 0 0). In a final difference Fourier map, highly disordered electron density was observed for compound **12**. The residual electron density was difficult to model and therefore the SQUEEZE routine in PLATON^[73]^ was used to eliminate this contribution of the electron density in the solvent region from the intensity data. The solvent‐free model was employed for the final refinement. It was estimated that each cavity contains 42 electrons which correspond to a solvent molecule of dichloromethane (CH_2_Cl_2_).

#### Synthesis of L_2_PhSnI (1)

Elemental iodine (1.226 g, 4.83 mmol) was dissolved in CH_2_Cl_2_ (90 mL) and slowly added dropwise to a solution of [2‐{(CH_2_O)_2_CH}C_6_H_4_]_2_SnPh_2_ (**A**) (2.759 g, 4.83 mmol) in CH_2_Cl_2_ (60 mL), at 0 °C. The reaction mixture was then left under vigorous stirring overnight. The crude was washed with a concentrated aqueous solution of Na_2_S_2_O_3_, then dried over Na_2_SO_4_ and separated by filtration. The organic solvent was removed in a rotary evaporator and pentane (50 mL) was added to the resulting oil to precipitate the title compound. The precipitate was filtered, washed with pentane (30 mL) and dried, resulting in 2.524 g (84 %) of a colourless crystalline solid, m.p. 107.8–108.2 °C. **Elemental analysis** calcd. for C_24_H_23_IO_4_Sn (621.06 g/mol): C, 46.41; H, 3.73; Found: C, 45.12; H, 3.62 %. ^**1**^ **H NMR** (CDCl_3_, 400.13 MHz, 21 °C), *δ* (ppm): 3.63 (m, 8H, *H*‐8, *H*‐9), 5.85 (s, 2H, *H*‐7), 7.37 (m, 3H, *H*‐5, *H*‐13), 7.45 (m, 4H, *H*‐4, *H*‐12), 7.52 (m, 2H, *H*‐3), 7.81 (dd, 2H, ^3^ *J*_H‐H_ =7.7 Hz, ^4^ *J*_H‐H_ =1.5 Hz, ^3^ *J*_Sn‐H_ =61 Hz, *H*‐11), 7.99 (m, 2H, ^3^ *J*_Sn‐H_ =75 Hz, *H*‐6). ^**13**^ **C{^1^H} NMR** (CDCl_3_, 100.62 MHz, 21 °C) *δ* (ppm): 64.65 (s, *C*‐8/9), 64.96 (s, *C*‐8/9), 103.16 (s, ^3^ *J*_Sn‐C_=19 Hz, *C*‐7), 127.09 (s, ^3^ *J*_Sn‐C_=59 Hz, C‐3), 128.45 (s, ^3^ *J*_117Sn‐C_=61 Hz, ^3^ *J*_119Sn‐C_=64 Hz, *C*‐12), 129.12 (s, ^4^ *J*_Sn‐C_=14 Hz, *C*‐13), 129.40 (s, ^3^ *J*_Sn‐C_=71 Hz, *C*‐5), 129.55 (s, ^4^ *J*_Sn‐C_=14 Hz, *C*‐4), 136.75 (s, ^2^ *J*_Sn‐C_=48 Hz, *C*‐6), 136.87 (s, ^2^ *J*_Sn‐C_=47 Hz, *C*‐11), 137.94 (s, ^1^ *J*_117Sn‐C_=715 Hz, ^1^ *J*_119Sn‐C_=748 Hz, *C*‐1), 141.04 (s, ^2^ *J*_Sn‐C_=38 Hz, *C*‐2), 142.25 (s, ^1^ *J*_117Sn‐C_=598 Hz, ^1^ *J*_119Sn‐C_=626 Hz, *C*‐10). ^**119**^ **Sn{^1^H} NMR** (CDCl_3_, 149.19 MHz, 21 °C) *δ* (ppm): −152.2. **HRMS** (ESI+, MeCN), *m/z* (relative intensity, %): [L_2_SnPh]^+^, calcd for C_24_H_23_O_4_Sn: 495.06128. Found 495.06185 (100). **IR** (ATR, υ, cm^−1^): ν(C−O−C) 948(s), 937(s).

#### Synthesis of L_2_SnI_2_ (2)

Elemental Iodine (1.912 g, 7.53 mmol) was added to a solution of **A** (2.151 g, 3.77 mmol) in CH_2_Cl_2_ (100 mL) and the mixture was stirred overnight. The crude was washed with a concentrated aqueous solution of Na_2_S_2_O_3_, then dried over Na_2_SO_4_ and separated by filtration. The organic solvent was removed in a rotary evaporator and the product was precipitated with pentane from the resulting oil. The compound was further washed with pentane and then dried to give 2.137 g (85 %) of a colourless crystalline solid, m.p. 206.4–206.6 °C. **Elemental analysis**: calcd. for: C_18_H_18_I_2_O_4_Sn (670.86 g/mol): C, 32.23; H, 2.70; Found: C, 33.08; H, 3.13. ^**1**^ **H NMR** (CDCl_3_, 600.13 MHz, 21 °C), *δ* (ppm): 3.70 (m, 8H, *H*‐8, *H*‐9), 5.91 (s, 2H, *H*‐7), 7.44 (dd, 2H, ^3^ *J*_H‐H_ =7.7 Hz, ^4^ *J*_H‐H_ =1.4 Hz, *H*‐3), 7.49 (td, 2H, ^3^ *J*_H‐H_=7.5 Hz, ^4^ *J*_H‐H_=1.5 Hz, *H*‐4), 7.61 (td, 2H, ^3^ *J*_H‐H_=7.4 Hz, ^4^ *J*_H‐H_=1.5 Hz, *H*‐5), 8.22 (dd, 2H, ^3^ *J*_H‐H_=7.5 Hz, ^4^ *J*_H‐H_=1.2 Hz, ^*3*^ *J*_Sn‐H_=111 Hz, *H*‐6). ^**13**^ **C{^1^H} NMR** (CDCl_3_, 150.92 MHz, 21 °C), *δ* (ppm): 65.10 (s, *C*‐8, *C*‐9), 102.04 (s, ^3^ *J*_Sn‐C_=21 Hz, C‐7), 127.10 (s, ^3^ *J*_Sn‐C_=81 Hz, *C*‐3), 129.86 (s, ^3^ *J*_117Sn‐C_=97 Hz, ^3^ *J*_119Sn‐C_=101 Hz, *C*‐5), 130.41 (s, ^4^ *J*_Sn‐C_=19 Hz, *C*‐4), 134.68 (s, ^2^ *J*_Sn‐C_=62 Hz, *C*‐6), 136.82 (s, ^1^ *J*_117Sn‐C_=961 Hz, ^1^ *J*_119Sn‐C_=1004 Hz, *C*‐1), 138.14 (s, ^2^ *J*_Sn‐C_=54 Hz, *C*‐2). ^**119**^ **Sn{^1^H} NMR** (CDCl_3_, 223.76 MHz, 21 °C) *δ* (ppm): −319.5. **HRMS** (APCI+, MeCN), *m/z* (relative intensity, %): [L_2_SnI]^+^, calcd. for C_18_H_18_IO_4_Sn: 544.92663. Found 544.92749 (100); [L'LSnI]^+^, calcd. for C_16_H_14_IO_3_Sn: 500.90041. Found 500.90125 (67); [L’_2_SnI]^+^, calcd. for C_14_H_10_IO_2_Sn: 456.87420. Found: 456.87500 (37); L’=2‐(O=CH)C_6_H_4_.

#### Synthesis of L_2_SnPhOSiPh_3_ (3)

A solution of Ph_3_SiONa (0.216 g, 0.72 mmol, 1.5 equiv.) in anhydrous THF (10 mL) was added dropwise to a solution of **1** (0.300 g, 0.48 mmol) in anhydrous THF (10 mL) and was left under stirring overnight. The solvent was removed in vacuo and anhydrous toluene (15 mL) was added to the mixture. The solution was filtered, and the solvent removed. The resulting solid was washed with small portions of Et_2_O and dried, resulting in 0.195 g (52 %) of a white solid, m.p. 119.5 °C. **Elemental analysis**: calcd. for C_42_H_38_O_5_SiSn (769.56 g/mol): C, 65.55; H, 4.98; Found: C, 65.73; H, 5.35.^**1**^ **H NMR** (CDCl_3_, 400.13 MHz, 20 °C), *δ* (ppm): 3.34–3.60 (m, 8H, *H*‐8, *H*‐9), 5.64 (s, 2H, *H*‐7), 7.17 (t, 6H, ^3^ *J*_H‐H_=7.3 Hz, H‐16), 7.24 (m, 2H, *H*‐12), 7.27 (m, 4H, *H*‐17, *H*‐13), 7.32 (m, 2H, *H*‐5). 7.41 (td, 2H, ^3^ *J*_H‐H_=7.4 Hz, ^4^ *J*_H‐H_=1.4 Hz, *H*‐4), 7.46 (m, 8H, *H*‐3, *H*‐15), 7.59 (m, 2H, *H*‐11), 7.85 (dd, 2H, ^3^ *J*_H‐H_=7.4 Hz, ^4^ *J*_H‐H_=1.4 Hz, ^3^ *J*_Sn,H_=68 Hz, *H*‐6). ^**13**^ **C{^1^H} NMR** (CDCl_3_, 100.62 MHz, 20 °C), *δ* (ppm): 64.54 (s, *C*‐9), 64.79 (s, *C*‐8), 103.32 (s, ^3^ *J*_Sn‐C_=20 Hz, *C*‐7), 126.84 (s, ^3^ *J*_Sn‐C_=61 Hz, *C*‐3), 127.24 (s, *C*‐16), 128.17 (s, ^3^ *J*_Sn‐C_=62 Hz, *C*‐12), 128.70 (s, *C*‐17), 128.71 (s, *C*‐13), 129.02 (s, ^3^ *J*_Sn‐C_=66 Hz, *C*‐5), 129.09 (s, ^4^ *J*_Sn‐C_=13 Hz, *C*‐4), 135.42 (s, *C*‐15), 136.28 (s, ^2^ *J*_Sn‐C_=41 Hz, *C*‐6), 136.77 (s, ^2^ *J*_Sn‐C_=45 Hz, *C*‐11), 139.54 (s, ^3^ *J*_Sn‐C_=79 Hz, *C*‐14), 139.55 (s, ^1^ *J*_117Sn,C_=792 Hz, ^1^ *J*_119Sn,C_=825 Hz, *C*‐1), 141.73 (s, ^2^ *J*_Sn‐C_=41 Hz, *C*‐2), 143.37 (s, ^1^ *J*_117Sn‐C_=665 Hz, ^1^ *J*_119Sn‐C_=695 Hz, *C*‐10).^**119**^ **Sn{^1^H} NMR** (CDCl_3_, 149.19 MHz, 19 °C), *δ* (ppm): −183.2. ^**29**^ **Si INEPT NMR** (CDCl_3_, 79.49 MHz, 19 °C), *δ* (ppm): −22.3. **HRMS** (APCI+, MeCN), *m/z* (relative intensity, %): [LL'SnPh]^+^, calcd. for C_22_H_19_O_3_Sn: 451.03507. Found 451.03555 (100); [L_2_SnPh]^+^, calcd. for C_24_H_23_O_4_Sn: 495.06128. Found 495.06165 (62); [L’_2_SnPh]^+^, calcd. for C_20_H_15_O_2_Sn: 407.00885. Found 407.00940 (57); [Ph_3_Si]^+^, calcd. for C_18_H_15_Si: 259.09375. Found 259.09393 (67); L’=2‐(O=CH)C_6_H_4_.

#### Synthesis of L_2_SnPhO^*t*^Bu (4)

A solution of ^*t*^BuOK (0.112 g, approx. 1 mL of 1 M solution in THF) was added dropwise to a solution of **1** (0.621 g, 1.00 mmol) in anhydrous THF (4 mL), and then stirred for 30 minutes. The resulting suspension was filtered through a cannula and the solvent was removed in vacuo, resulting in a colourless, viscous oil. The title compound was obtained as a white crystalline solid 0.400 g (71 %) upon crystallisation from acetonitrile at −24 °C, m.p.=106 °C. ^**1**^ **H NMR** (C_6_D_6_, 400.13 MHz, 22 °C), *δ* (ppm): 1.45 (s, 9H, *H*‐15), 3.00–3.23 (m, 8H, *H*‐8, *H*‐9), 5.77 (s, 4H, ^4^ *J*_Sn‐H_=7 Hz, *H*‐7), 7.15–7.23 (m, 3H, *H*‐5, *H*‐13), 7.25 (td, 2H, ^3^ *J*_H‐H_=7.4 Hz, ^4^ *J*_H‐H_=1.5 Hz, *H*‐4), 7.30 (m, 2H, *H*‐12), 7.60 (m, 2H, *H*‐3), 8.15 (d, 2H, ^3^ *J*_H‐H_=8.0 Hz, ^4^ *J*_H‐H_=1.4 Hz, *H*‐11), 8.39 (m, 4H, ^3^ *J*_Sn‐H_=61 Hz, *H*‐6). ^**13**^ **C{^1^H} NMR** (C_6_D_6_, 100.62 MHz, 22 °C), *δ* (ppm): 34.32 (s, ^3^ *J*_Sn‐C_=13 Hz, *C*‐15), 64.57 (s, *C*‐8/*C*‐9), 64.70 (s, *C*‐8/*C*‐9), 71.85 (s, ^2^ *J*_Sn‐C_=31 Hz, *C*‐14), 104.04 (s, ^3^ *J*_Sn‐C_=23 Hz, *C*‐7), 127.18 (s, ^3^ *J*_Sn‐C_=56 Hz, *C*‐3), 128.52 (s, *C*‐12), 128.97 (s, *C*‐4/*C*‐5/*C*‐13), 129.10 (s, *C*‐4/*C*‐5/*C*‐13), 137.06 (s, ^2^ *J*_Sn‐C_=35 Hz, *C*‐6), 137.41 (s, ^2^ *J*_Sn‐C_=43 Hz, *C*‐11), 141.82 (s, ^1^ *J*_117Sn‐C_=722 Hz, ^1^ *J*_119Sn‐C_=755 Hz, *C*‐1), 143.40 (s, ^2^ *J*_Sn‐C_=40 Hz, *C*‐2), 144.82 (s, ^1^ *J*_117Sn‐C_=660 Hz, ^1^ *J*_119Sn‐C_=691 Hz, *C*‐10). ^**119**^ **Sn{^1^H} NMR** (C_6_D_6_, 149.19 MHz, 21 °C) *δ* (ppm): −191.0.

#### Synthesis of (L_2_SnPh)_2_O (5)

In a 100 mL Schlenk flask a solution of **1** (1.00 g, 1.06 mmol) in anhydrous THF (25 mL) was added dropwise to a suspension of ^*t*^BuOK (0.208 g, 1.85 mmol, 1.15 equiv.) in anhydrous THF (25 mL), and the mixture was left under stirring overnight. The solvent was removed in vacuo and anhydrous Et_2_O (30 mL) was added to extract the product. The etheric solution was transferred into a new Schlenk flask, then the solvent was removed in vacuo and the resulting solid was dried. The product was obtained as a white solid, 0.699 g (76 %), m.p. 70.3–72.9 °C. **Elemental analysis**: calcd. for C_48_H_46_O_9_Sn_2_ (1004.31 g/mol): C, 57.71; H, 4.62; O, 14.34; Found: C, 57.70; H, 4.68. ^**1**^ **H NMR** (C_6_D_6_, 600.13 MHz, 21 °C), *δ* (ppm): 3.04–3.30 (m, 16H, *H*‐8, *H*‐9), 5.75 (s, 4H, ^4^ *J*_Sn‐H_=7 Hz, *H*‐7), 7.16 (m, 12H, *H*‐4, *H*‐5, *H*‐12), 7.20 (td, 2H, ^3^ *J*_H‐H_=7.5 Hz, ^4^ *J*_H‐H_=1.6 Hz, *H*‐13), 7.58 (dd, 4H, ^3^ *J*_H‐H_=7.5 Hz, ^4^ *J*_H‐H_=1.5 Hz, ^4^ *J*_Sn‐H_=31 Hz, *H*‐3), 7.86 (m, 4H, *H*‐11), 8.20 (dd, 4H, ^3^ *J*_H‐H_=7.2 Hz, ^4^ *J*_H‐H_=1.6 Hz, ^3^ *J*_Sn‐H_=63 Hz, *H*‐6). ^**13**^ **C{^1^H} NMR** (C_6_D_6_, 150.92 MHz, 21 °C), *δ* (ppm): 64.46 (s, *C*‐8/*C*‐9), 64.71 (s, *C*‐8/*C*‐9), 103.99 (s, ^3^ *J*_Sn‐C_=21 Hz, *C*‐7), 126.84 (s, ^3^ *J*_Sn‐C_=55 Hz, *C*‐3), 128.35 (s, *C*‐4/*C*‐5/*C*‐12/*C*‐13), 128.38 (s, *C*‐4/*C*‐5/*C*‐12/*C*‐13), 128.51 (s, *C*‐4/*C*‐5/*C*‐12/*C*‐13), 128.56 (s, *C*‐4/*C*‐5/*C*‐12/*C*‐13), 137.16 (s, ^2^ *J*_Sn‐C_=39 Hz, *C*‐6), 137.53 (s, ^*2*^ *J*_Sn‐C_=44 Hz, *C*‐11), 142.98 (s, ^1^ *J*_117Sn‐C_=720 Hz, ^1^ *J*_119Sn‐C_=755 Hz, *C*‐1), 143.43 (s, ^2^ *J*_Sn‐C_=38 Hz, *C*‐2), 145.86 (s, ^1^ *J*_117Sn‐C_=641 Hz, ^1^ *J*_119Sn‐C_=670 Hz, *C*‐10). ^**119**^ **Sn{^1^H} NMR** (C_6_D_6_, 223.76 MHz, 21 °C), *δ* (ppm): −155.9. **HRMS** (APCI+, MeCN), *m/z* (relative intensity, %): [L_2_SnPh]^+^, calcd. for C_24_H_23_O_4_Sn: 495.06128. Found 495.06193 (100); [L'LSnPh]^+^, calcd. for C_22_H_19_O_3_Sn: 451.03507. Found 451.03671 (33); [L’_2_SnPh]^+^, calcd. for C_20_H_15_O_2_Sn: 407.00885. Found 407.01052 (9); L’=2‐(O=CH)C_6_H_4_.

#### Synthesis of L_2_SnPhOMe (6)

Clean sodium (0.100 g, 4.35 mmol) was washed with petroleum ether and added into MeOH (25 mL) in a 2‐neck round‐bottom flask equipped with a Liebig condenser and a vacuum adapter. The mixture was left with no stirring, under continuous argon flow until the sodium was consumed in the reaction. Compound **1** (1.00 g, 1.61 mmol) was added under stirring and the mixture was heated at reflux at 70 °C for 4 hours. The solvent was removed in vacuo and anhydrous toluene (20 mL) was added. The resulting suspension was filtered through a canula, and the solvent was evaporated. The compound was washed with anhydrous Et_2_O (20 mL) and dried in vacuum, to yield 0.671 g (79 %) of a white solid. The title compound was obtained as the major product (86 %) in a mixture with **5**, one of its decomposition products. m.p.=71 °C. ^**1**^ **H NMR** (C_6_D_6_, 600.13 MHz, 21 °C), *δ* (ppm): 3.05–3.30 (m, 8H, *H*‐8, *H*‐9), 4.00 (s, 3H, ^3^ *J*_Sn‐H_=38 Hz, ‐OC*H*_3_), 5.69 (s, 2H, ^4^ *J*_Sn‐H_=7 Hz, *H*‐7), 7.19 (m, 4H, *H‐*12, *H*‐4), 7.23 (m, 1H, *H*‐13), 7.31 (t, 2H, ^3^ *J*_H‐H_=7.6 Hz, *H*‐5), 7.54 (dd, 2H, ^3^ *J*_H‐H_=7.1 Hz, ^4^ *J*_H‐H_=1.9 Hz, *H*‐3), 8.07 (dd, ^3^ *J*_H‐H_=7.7 Hz, ^4^ *J*_H‐H_=1.2 Hz, ^3^ *J*_Sn‐H_=54 Hz, *H*‐11), 8.20 (dd, ^3^ *J*_H‐H_=7.0 Hz, ^4^ *J*_H‐H_=1.4 Hz, ^3^ *J*_Sn‐H_=63 Hz, *H*‐6) ^**119**^ **Sn{^1^H} NMR** (C_6_D_6_, 223.76 MHz, 21 °C) *δ* (ppm): −154.2.

#### Synthesis of L_2_SnPhOH (7)

**(Route 1)** An aqueous solution of KOH (0.200 g, 3.56 mmol, 2 mL H_2_O) was added to a solution of **1** (0.225 g, 0.36 mmol) in CH_2_Cl_2_ (5 mL) and stirred vigorously for 20 minutes. The reaction mixture was then extracted with CH_2_Cl_2_ (2x10 mL) and the resulting organic phases were combined and dried over Na_2_SO_4_. The title compound was obtained quantitatively as a colourless oil after filtration and removal of the organic solvent.

**(Route 2)** Compound **5** (0.045 g, 0.04 mmol) was dissolved in CH_2_Cl_2_ (5 mL) and stirred overnight with distilled water (6 mL). The mixture was then extracted with CH_2_Cl_2_ (3x5 mL), and the combined organic layers were dried over Na_2_SO_4_. The solvent was removed using a rotary evaporator after filtration to produce a colourless oil, 0.027 g (60 %). ^**1**^ **H NMR** (CDCl_3_, 400.13 MHz, 20 °C), *δ* (ppm): 0.69 (s, 1H, ^2^ *J*_Sn‐H_=24 Hz, O*H*), 3.62 (m, 8H, *H*‐8, *H*‐9), 5.77 (s, 2H, ^4^ *J*_Sn‐H_=7 Hz, *H*‐7), 7.38 (m, 3H, *H*‐5, *H*‐13), 7.43 (m, 4H, *H*‐4, *H*‐12), 7.49 (m, 2H, *H*‐3), 7.77 (m, 2H, *H*‐11), 7.88 (m, 2H, *H*‐6). ^**13**^ **C{^1^H} NMR** (CDCl_3_, 100.62 MHz, 21 °C) *δ* (ppm): 64.81 (s, *C*‐8), 64.83 (s, *C*‐9), 103.75 (s, ^3^ *J*_Sn‐C_=20 Hz, *C*‐7), 127.43 (s, ^3^ *J*_117Sn‐C_=55 Hz, ^3^ *J*_119Sn‐C_=57 Hz, *C*‐3), 128.44 (s, ^3^ *J*_117Sn‐C_=59 Hz, ^3^ *J*_119Sn‐C_=62 Hz, *C*‐5), 129.03 (s, ^4^ *J*_Sn,C_=13 Hz, *C*‐13), 129.23 (s, *C*‐12), 129.29 (s, ^4^ *J*_Sn,C_=*C*‐4), 136.45 (s, *C*‐6), 136.65 (s, *C*‐11), 138.99 (s, *C*‐1), 141.88 (s, ^2^ *J*_Sn‐C_=38 Hz, *C*‐2), 142.62 (s, *C*‐10). ^**119**^ **Sn{^1^H} NMR** (C_6_D_6_, 223.8 MHz, 23 °C) *δ* (ppm): −155.0. ^**119**^ **Sn{1H} NMR** (CDCl_3_, 149.19 MHz, 20 °C) *δ* (ppm): −155.4.

#### Synthesis of (L_2_Sn)_3_O_3_ (8)

An aqueous solution of KOH (0.209 g, 3.75 mmol, 5 equiv., 5 mL H_2_O) was added to a solution of **2** (0.250 g, 0.37 mmol) in THF (20 mL) and the resulting mixture was stirred overnight. After removal of the organic solvent, the title compound was extracted with 3×20 mL CH_2_Cl_2_. The combined organic phases were dried over anhydrous Na_2_SO_4_, filtered and the solvent was removed in a rotary evaporator. Pentane was added to the resulting oil and the resulting precipitate is dried, affording 0.157 g (98 %) isolated product, m.p.=165.7–167.4 °C. **Elemental analysis**: calcd. for: C_54_H_54_O_15_Sn_3_ (1299.14 g/mol): C, 49.92; H, 4.19; Found: C, 50.14; H, 4.45. ^**1**^ **H NMR** (CDCl_3_, 600.13 MHz, 21 °C), *δ* (ppm): 3.48–3.73 (m, 24H, *H*‐8, *H*‐9), 5.65 (s, 6H, *H*‐7), 7.14 (t, 6H, ^3^ *J*_H‐H_=7.2 Hz, *H*‐4), 7.20–7.27 (m, 12H, *H*‐3, *H*‐5), 7.97 (d, 6H, ^3^ *J*_H‐H_=7.3 Hz, ^3^ *J*_Sn‐H_=73 Hz, *H*‐6). ^**13**^ **C{^1^H} NMR** (CDCl_3_, 150.92 MHz, 21 °C), *δ* (ppm): 64.77 (s, *C*‐8, *C*‐9), 103.34 (s, ^3^ *J*_Sn‐C_=24 Hz, *C*‐7), 126.33 (s, ^3^ *J*_Sn‐C_=72 Hz, *C*‐3), 128.25 (s, ^4^ *J*_Sn‐C_=14 Hz, *C*‐4), 128.43 (s, ^3^ *J*_Sn‐C_=73 Hz, *C*‐5), 136.16 (s, ^2^ *J*_Sn‐C_=35 Hz, *C*‐6), 141.31 (s, ^2^ *J*_Sn‐C_=50 Hz, *C*‐2), 142.51 (s, ^1^ *J*_117Sn‐C_=974 Hz, ^1^ *J*_119Sn‐C_=1019 Hz, *C*‐1). ^**119**^ **Sn{^1^H} NMR** (CDCl_3_, 223.8 MHz, 21 °C) *δ* (ppm): −214.2. **HRMS** (APCI+, MeCN), *m/z* (relative intensity, %): [LL'SnOH]^+^, calcd. for C_16_H_15_O_4_Sn: 390.99868. Found 390.99754 (100); [L_2_SnOH]^+^ calcd. for C_18_H_19_O_5_Sn: 435.02490. Found: 435.02344 (53); [LL'SnH]^+^, calcd. for C_16_H_15_O_3_Sn: 375.00377. Found 375.00270 (49); [L’_2_SnOH]^+^, calcd. for C_14_H_11_O_3_Sn: 346.97247. Found 346.97148 (34); [L’_2_SnH]^+^, calcd. for C_14_H_11_O_2_Sn: 330.97755. Found 330.97664 (46); L’=2‐(O=CH)C_6_H_4_.

#### Synthesis of L_2_Sn(OSiPh_3_)_2_ (10)

In a 50 mL Schlenk flask a solution of the compound Ph_3_SiONa (0.300 g, 1.0 mmol, 1.1 equiv.) in anhydrous THF (6 mL) was added dropwise to a solution of **2** (0.300 g, 0.48 mmol) in anhydrous THF (11 mL) and the mixture was stirred overnight. The solvent was removed in vacuum and anhydrous toluene (12 mL) was added to the mixture. The obtained suspension was filtered in another Schenk flask and toluene was then removed in vacuum, giving a colourless solid. The product was crystallised by layering hexane over a concentrated THF solution of **10** resulting in 0.317 g (73 %) colourless crystals. m.p.=207.5–209.8 °C. **Elemental analysis**: calcd. for: C_54_H_48_O_6_Si_2_Sn (967.85 g/mol): C, 67.01; H, 5.00; Found: C, 67.94; H, 5.17. ^**1**^ **H NMR** (CDCl_3_, 600.13 MHz, 21 °C), *δ* ppm: 3.03–3.63 (m, 8H, *H*‐8, *H*‐9), 5.01 (s, 2H, ^4^ *J*_Sn‐H_=10 Hz, *H*‐7), 7.09 (t, 12H, ^3^ *J*_H‐H_=7.5 Hz, *H*‐12), 7.24 (m, 8H, *H*‐3, *H*‐13), 7.35 (td, 2H, ^3^ *J*_H‐H_=7.4 Hz, ^4^ *J*_H‐H_=1.4 Hz, *H*‐5), 7.39 (td, 2H, ^3^ *J*_H‐H_=7.4 Hz, ^4^ *J*_H‐H_=1.4 Hz, *H*‐4), 7.45 (d, 12H, ^3^ *J*_H‐H_=7.0 Hz, *H*‐11), 8.10 (d, 2H, ^3^ *J*_H‐H_=7.2 Hz, ^3^ *J*_Sn‐H_=87 Hz, *H*‐6). ^**13**^ **C{^1^H} NMR** (CDCl_3_, 150.92 MHz, 21 °C), *δ* ppm: 64.53 (s, *C*‐8, *C*‐9), 102.03 (s, ^3^ *J*_Sn‐C_=28 Hz, *C*‐7),127.03 (s, *C*‐3), 127.22 (s, *C*‐12), 128.70 (s, *C*‐13), 129.52 (s, *C*‐4, *C*‐5), 135.17 (s, *C*‐6), 135.48 (s, *C*‐11), 137.69 (s, *C*‐1), 139.24 (s, *C*‐10), 139.87 (s, *C*‐2). ^**119**^ **Sn{^1^H} NMR** (CDCl_3_, 223.76 MHz, 21 °C) *δ* (ppm): −335.6 **HRMS** (APCI+, MeCN), *m/z* (relative intensity, %): [LL'SnPh]^+^, calcd. for C_22_H_19_O_3_Sn: 451.03507. Found 451.03398 (100); [L_2_SnOSiPh_3_]^+^, calcd. for C_36_H_33_O_5_SiSn: 693.11137. Found 693.10999 (84)_;_ [Ph_3_Si]^+^, calcd. for C_18_H_15_Si: 259.09375. Found 259.09236 (56)_;_ L’=2‐(O=CH)C_6_H_4_.

#### Synthesis of L_2_Sn(O^*t*^Bu)_2_ (11)

A solution of ^*t*^BuOK (0.167 g, approx. 1.5 mL of 1 M solution in THF) was added dropwise to a solution of **2** (0.500 g, 0.75 mmol) in anhydrous THF (20 mL), and the reaction mixture was stirred for 30 minutes. The resulting suspension was filtered through a cannula and the solvent removed in vacuum, resulting a colourless, viscous oil. Colourless crystals of the title compound, 0.344 g (82 %), were isolated after one day upon cooling a concentrated MeCN solution to −24 °C. m.p.=180–182 °C. ^**1**^ **H NMR** (C_6_D_6_, 400.13 MHz, 21 °C), *δ* (ppm): 1.52 (s, 18H, *H*‐11), 3.00 (m, 4H, *H*‐8/*H*‐9), 3.29 (m, 4H, *H*‐8/*H*‐9), 5.53 (s, 2H, ^4^ *J*_Sn‐H_=8 Hz, *H*‐7), 7.17 (td, 2H, ^3^ *J*_H‐H_=7.5 Hz, ^4^ *J*_H‐H_=1.4 Hz, *H*‐3), 7.33 (td, 2H, ^3^ *J*_H‐H_=7.4 Hz, ^4^ *J*_H‐H_=1.4 Hz, *H*‐4), 7.39 (m, 2H, *H*‐5), 8.80 (dd, 2H, ^3^ *J*_H‐H_=7.5 Hz, ^4^ *J*_H‐H_=1.4 Hz, ^3^ *J*_Sn‐H_=73 Hz, *H*‐6). ^**13**^ **C{^1^H} NMR** (C_6_D_6_, 100.62 MHz, 21 °C), *δ* (ppm): 34.28 (s, ^3^ *J*_Sn‐C_=16 Hz, *C*‐11), 64.57 (s, C‐*8*, C‐*9*), 72.06 (s, ^2^ *J*_Sn‐C_=36 Hz, *C*‐10), 103.09 (s, ^3^ *J*_Sn‐C_=24 Hz, *C*‐7), 127.57 (s, *C*‐3), 129.01 (s, ^4^ *J*_Sn‐C_=15 Hz, *C*‐4), 129.18 (s, ^3^ *J*_117Sn‐C_=73 Hz, ^3^ *J*_119Sn_‐_C_=76 Hz, *C*‐5), 136.69 (s, ^2^ *J*_Sn‐C_=25 Hz, *C*‐6), 141.00 (s, ^2^ *J*_Sn‐C_=53 Hz, *C*‐2), 142.48 (s, ^1^ *J*_117Sn‐C_=1053 Hz, ^1^ *J*_119Sn‐C_=1103 Hz, *C*‐1). ^**119**^ **Sn{^1^H} NMR** (C_6_D_6_, 149.19 MHz, 21 °C) *δ* (ppm): −307.5.

#### Synthesis of (L_2_SnO)_2_OB(*m–*tol) (12)

Compound **8** (0.440 g, 0.34 mmol) and 3‐Me‐C_6_H_4_B(OH)_2_ (0.069 g, 0.51 mmol) were dissolved in toluene (60 mL) and heated at reflux in a 100 mL round‐bottom flask equipped with a Dean‐Stark apparatus and an air condenser, for 22 h. Removal of the solvent using a rotary evaporator yielded a colourless oil. Pentane was added to the resulting oil affording the title compound as a white precipitate, 0.473 g (95 %). m.p.=191–194 °C. **Elemental analysis**: calcd. for: C_43_H_43_BO_11_Sn_2_ (984.04 g/mol): C, 52.49; H, 4.40; Found: C, 53.46; H, 4.78. ^**1**^ **H NMR** (CDCl_3_, 400.13 MHz, 19 °C), *δ* (ppm): 2.32 (s, 3H, *H*‐16), 3.75 (m, 8H, *H*‐8/*H*‐9), 3.92 (m, 8H, *H*‐8/*H*‐9), 5.87 (s, 4H, ^4^ *J*_Sn‐H_=7 Hz, *H*‐7,), 7.11 (m, 1H, *H*‐13), 7.18 (t, 1H, ^3^ *J*_H‐H_=7.6 Hz, *H*‐12), 7.24 (td, 4H, ^3^ *J*_H‐H_=7.2 Hz, ^4^ *J*_H,H_=1.3 Hz, *H*‐5), 7.34 (td, 4H, ^3^ *J*_H‐H_=7.4 Hz, ^4^ *J*_H‐H_=1.4 Hz, *H*‐4), 7.39 (m, 4H, *H*‐3), 7.72 (m, 2H, H‐11, H‐15), 7.97 (d, 4H, ^3^ *J*_H‐H_=7.1 Hz, ^3^ *J*_Sn‐H_=78 Hz, H‐6). ^**13**^ **C{^1^H} NMR** (CDCl_3_, 100.62 MHz, 19 °C), *δ* (ppm): 21.80 (s, *C*‐16), 64.96 (s, *C*‐8/*C*‐9), 65.03 (s, *C*‐8/*C*‐9), 103.01 (s, ^3^ *J*_Sn‐C_=25 Hz, *C*‐7), 126.80 (s, ^3^ *J*_Sn‐C_=79 Hz, *C*‐3), 127.04 (s, *C*‐12), 129.09 (s, ^3^ *J*_Sn‐C_=82 Hz, *C*‐5), 129.11 (s, ^4^ *J*_Sn‐C_=15 Hz, *C*‐4), 129.32 (s, *C*‐13), 131.88 (s, *C*‐11), 135.61 (s, ^2^ *J*_Sn‐C_=38 Hz, *C*‐6), 135.70 (s, *C*‐15), 136.01 (s, *C*‐10), 139.54 (s, ^1^ *J*_117Sn‐C_=1047 Hz, ^1^ *J*_119Sn‐C_=1097 Hz, *C*‐1), 141.02 (s, ^2^ *J*_Sn‐C_=54 Hz, *C*‐2). ^**119**^ **Sn{^1^H} NMR** (CDCl_3_, 149.19 MHz, 21 °C) *δ* (ppm): −258.7. ^**11**^ **B{^1^H} NMR** (CDCl_3_, 128.38 MHz, 21 °C) *δ* (ppm): 28.9 (br). **HRMS** (APCI+, MeCN), *m/z* (relative intensity, %): [M+H]^+^ calcd. for C_43_H_44_BO_11_Sn_2_: 985.10092. Found 985.10458 (100).

## Conflict of Interests

The authors declare no conflict of interest.

## Supporting information

As a service to our authors and readers, this journal provides supporting information supplied by the authors. Such materials are peer reviewed and may be re‐organized for online delivery, but are not copy‐edited or typeset. Technical support issues arising from supporting information (other than missing files) should be addressed to the authors.

Supporting Information

## Data Availability

The data that support the findings of this study are available in the supplementary material of this article.

## References

[1] X. Liu , M. Gong , S. Deng , T. Zhao , J. Zhang , D. Wang , J. Mater. Chem. A 2020, 8, 10130–10149.

[2] M. J. Hampden-Smith , T. A. Wark , C. J. Brinker , Coord. Chem. Rev. 1992, 112, 81–116.

[3] S. Mishra , S. Daniele , Chem. Rev. 2015, 115, 8379–8448. 26186083 10.1021/cr400637c

[4] U. Schubert , Acc. Chem. Res. 2007, 40, 730–737. 17518436 10.1021/ar600036x

[5] A. G. Davies , D. C. Kleinschmidt , P. R. Palan , S. C. Vasishtha , J. Chem. Soc. C 1971, 3972–3976.

[6] J. Beckmann , K. Jurkschat , M. Schürmann , J. Organomet. Chem. 2001, 626, 49–52.

[7] H. Yasuda , J.-C. Choi , S.-C. Lee , T. Sakakura , J. Organomet. Chem. 2002, 659, 133–141.

[8] M. Suzuki , I. H. Son , R. Noyori , H. Masuda , Organometallics 1990, 9, 3043–3053.

[9] G. D. Smith , V. M. Visciglio , P. E. Fanwick , I. P. Rothwell , Organometallics 1992, 11, 1064–1071.

[10] A. G. Lichtscheidl , M. T. Janicke , B. L. Scott , A. T. Nelson , J. L. Kiplinger , Dalton Trans. 2015, 44, 16156–16163. 26295362 10.1039/c5dt01980j

[11] G. D. Smith , P. E. Fanwick , I. P. Rothwell , J. Am. Chem. Soc. 1989, 111, 750–751.

[12] S. Beaini , G. B. Deacon , M. Hilder , P. C. Junk , D. R. Turner , Eur. J. Inorg. Chem. 2006, 2006, 3434–3441.

[13] M. H. Chisholm , J. C. Gallucci , C. Krempner , Polyhedron 2007, 26, 4436–4444.

[14] Z. Padělková , T. Weidlich , L. Kolářová , A. Eisner , I. Císařová , T. A. Zevaco , A. Růžička , J. Organomet. Chem. 2007, 692, 5633–5645.

[15] T. J. Boyle , J. M. Sears , D. Perales , R. E. Cramer , P. Lu , R. O. Chan , B. A. Hernandez-Sanchez , Inorg. Chem. 2018, 57, 8806–8820. 29979585 10.1021/acs.inorgchem.8b00630

[16] P. A. Williams , J. L. Roberts , A. C. Jones , P. R. Chalker , J. F. Bickley , A. Steiner , H. O. Davies , T. J. Leedham , J. Mater. Chem. 2002, 12, 165–167.

[17] K. Samedov , Y. Aksu , M. Driess , ChemPlusChem 2012, 77, 663–674.

[18] J. Beckmann , K. Jurkschat , Coord. Chem. Rev. 2001, 215, 267–300.

[19] D. Balkenhol , J. Beckmann , K. Jurkschat , M. Schürmann , Z. Anorg. Allg. Chem. 2004, 630, 1875–1878.

[20] B. Morosin , L. A. Harrah , Acta Crystallogr., Sect. B: Struct. Crystallogr. Cryst. Chem. 1981, 37, 579–586.

[21] P. Brown , M. F. Mahon , K. C. Molloy , J. Chem. Soc., Dalton Trans. 1990, 2643–2651.

[22] B. J. Brisdon , M. F. Mahon , K. C. Molloy , P. J. Schofield , J. Organomet. Chem. 1994, 465, 145–151.

[23] M. Gopalakrishnan , N. Palanisami , RSC Adv. 2016, 6, 1760–1768.

[24] J. Ayari , C. R. Göb , I. M. Oppel , M. Lutter , W. Hiller , K. Jurkschat , Angew. Chem. Int. Ed. 2020, 59, 23892–23898. 10.1002/anie.202012248 PMC7756359 32964645

[25] A. G. Davies, *Organotin Chemistry, 2nd, Completely Revised and Updated Edition*, WILEY-VCH Verlag GmbH & Co. KGaA, **2004**, 179–195.

[26] V. Chandrasekhar , S. Nagendran , V. Baskar , Coord. Chem. Rev. 2002, 235, 1–52.

[27] R. K. Harris , A. Sebald , J. Organomet. Chem. 1987, 331, C9–C12.

[28] S. Masamune , L. R. Sita , D. J. Williams , J. Am. Chem. Soc. 1983, 105, 630–631.

[29] H. Grützmacher , H. Pritzkow , Chem. Ber. 1993, 126, 2409–2413.

[30] J. Beckmann , K. Jurkschat , S. Rabe , M. Schürmann , Z. Anorg. Allg. Chem. 2001, 627, 2413–2419.

[31] C. Glidewell , D. C. Liles , Acta Crystallogr., Sect. B: Struct. Crystallogr. Cryst. Chem. 1978, 34, 1693–1695.

[32] T. P. Lockhart , H. Puff , W. Schuh , H. Reuter , T. N. Mitchell , J. Organomet. Chem. 1989, 366, 61–72.

[33] I. Wharf , A.-M. Lebuis , G. A. Roper , Inorg. Chim. Acta 1999, 294, 224–231.

[34] Z. Padělková , M. S. Nechaev , Z. Černošek , J. Brus , A. Růžička , Organometallics 2008, 27, 5303–5308.

[35] A. R. Browne , N. Deligonul , B. L. Anderson , M. Zeller , A. D. Hunter , T. G. Gray , Chem. Commun. 2015, 51, 15800–15803. 10.1039/c5cc05200a 26365433

[36] B. Mairychová , T. Svoboda , P. Štěpnička , A. Růžička , R. W. A. Havenith , M. Alonso , F. D. Proft , R. Jambor , L. Dostál , Inorg. Chem. 2013, 52, 1424–1431. 23327544 10.1021/ic302153s

[37] L. Dostál , R. Jambor , A. Růžička , R. Jirásko , A. Lyčka , J. Beckmann , S. Ketkov , Inorg. Chem. 2015, 54, 6010–6019. 26016827 10.1021/acs.inorgchem.5b00893

[38] M. Kořenková , M. Erben , R. Jambor , A. Růžička , L. Dostál , J. Organomet. Chem. 2014, 772–773, 287–291.

[39] M. Kořenková , B. Mairychová , A. Růžička , R. Jambor , L. Dostál , Dalton Trans. 2014, 43, 7096–7108. 24671260 10.1039/c3dt53012d

[40] Y. Milasheuskaya , J. Schwarz , L. Dostál , Z. Růžičková , M. Bouška , Z. Olmrová Zmrhalová , T. Syrový , R. Jambor , Dalton Trans. 2021, 50, 18164–18172. 34859799 10.1039/d1dt02975d

[41] M. Kořenková , B. Mairychová , R. Jambor , Z. Růžičková , L. Dostál , Inorg. Chem. Commun. 2014, 47, 128–130.

[42] I. Barbul , R. A. Varga , C. Silvestru , Eur. J. Inorg. Chem. 2013, 3146–3154.

[43] I. Barbul , R. A. Varga , K. C. Molloy , C. Silvestru , Dalton Trans. 2013, 42, 15427–15436. 24014046 10.1039/c3dt52022f

[44] A.-A. Someşan , I. Barbul , S.-M. Vieriu , R. A. Varga , C. Silvestru , Dalton Trans. 2019, 48, 6527–6538. 31011717 10.1039/c9dt00817a

[45] A.-A. Someşan , I. Barbul , S.-M. Vieriu , R. A. Varga , Rev. Roum. Chim. 2020, 65, 725–733.

[46] A.-A. Someşan , C. Silvestru , R. A. Varga , New J. Chem. 2021, 45, 3817–3827.

[47] A.-A. Someşan , S.-M. Vieriu , A. Crăciun , C. Silvestru , P. Chiroi , A. Nutu , A. Jurj , R. Lajos , I. Berindan-Neagoe , R. A. Varga , Appl. Organomet. Chem. 2022, 36, e6540.

[48] P. Wytrych , J. Utko , J. Kłak , M. Ptak , M. Stefanski , T. Lis , J. Ejfler , Ł. John , Molecules 2022, 27, 147. 10.3390/molecules27010147 PMC8746333 35011379

[49] R. A. Varga , A. Rotar , M. Schuermann , K. Jurkschat , C. Silvestru , Eur. J. Inorg. Chem. 2006, 1475–1486.

[50] H. Kameo , T. Kawamoto , S. Sakaki , D. Bourissou , H. Nakazawa , Eur. J. Inorg. Chem. 2019, 3045–3052.

[51] R. Alan Howie , J. L. Wardell , Acta Crystallogr., Sect. C: Struct. Chem. 1996, 52, 1424–1426.

[52] G. Ferguson , J. N. Low , J.-N. Ross , E. J. Storey , J. L. Wardell , Main Group Met. Chem. 1999, 22, 453–462.

[53] P. Švec , Z. Růžičková , P. Vlasák , J. Turek , F. De Proft , A. Růžička , J. Organomet. Chem. 2016, 801, 14–23.

[54] A.-A. Somesan , R. A. Varga , C. Silvestru , Rev. Roum. Chim. 2015, 60, 1097–1106.

[55] B. Kašná , R. Jambor , L. Dostál , A. Růžička , I. Císařová , J. Holeček , Organometallics 2004, 23, 5300–5307.

[56] R. Jambor , L. Dostál , A. Růžička , I. Císařová , J. Brus , M. Holčapek , J. Holeček , Organometallics 2002, 21, 3996–4004.

[57] H. S. van den Bogaard , H. K. Sharma , A. Mettta-Magaña , K. H. Pannell , Chem. Asian J. 2023, 18, e202300767. 37738358 10.1002/asia.202300767

[58] L. Dostál , R. Jambor , A. Růžička , R. Jirásko , J. Taraba , J. Holeček , J. Organomet. Chem. 2007, 692, 3750–3757.

[59] M. Mehring , M. Schuermann , K. Jurkschat , Organometallics 1998, 17, 1227–1236.

[60] B. Cordero , V. Gómez , A. E. Platero-Prats , M. Revés , J. Echeverría , E. Cremades , F. Barragán , S. Alvarez , Dalton Trans. 2008, 2832–2838. 18478144 10.1039/b801115j

[61] S. Alvarez , Dalton Trans. 2013, 42, 8617–8636. 23632803 10.1039/c3dt50599e

[62] Z. Padělková , P. Švec , H. Kampová , J. Sýkora , M. Semler , P. Štěpnička , S. Bakardjieva , R. Willem , A. Růžička , Organometallics 2013, 32, 2398–2405.

[63] Z. Padělková , P. Švec , V. Pejchal , A. Růžička , Dalton Trans. 2013, 42, 7660–7671. 23538786 10.1039/c3dt50278c

[64] M. H. Chisholm , E. E. Delbridge , J. C. Gallucci , New J. Chem. 2004, 28, 145–152.

[65] A. W. Addison , T. N. Rao , J. Reedijk , J. van Rijn , G. C. Verschoor , J. Chem. Soc., Dalton Trans. 1984, 1349–1356.

[66] M. Beuter , U. Kolb , A. Zickgraf , E. Bräu , M. Bletz , M. Dräger , Polyhedron 1997, 16, 4005–4015.

[67] J.-C. Choi , T. Sakakura , T. Sako , J. Am. Chem. Soc. 1999, 121, 3793–3794.

[68] P. Brown , M. F. Mahon , K. C. Molloy , J. Chem. Soc., Dalton Trans. 1992, 3503–3509.

[69] *MestReNova, version 14.0.0-23239, Mestrelab Research S. L., Feliciano Barrera 9B, Bajo, 15706 Santiago de Compostela, Spain*, **2020**.

[70] *Qual Browser Thermo Xcalibur, version 4.0.27.19, ThermoFischer Scientific Inc.: Waltham, MA, 02454*, **2016**.

[71] G. M. Sheldrick , Acta Crystallogr., Sect. A: Found. Crystallogr. 2015, 71, 3–8. 10.1107/S2053273314026370 PMC4283466 25537383

[72] DIAMOND – Visual Crystal Structure Information System, CRYSTAL IMPACT: Postfach 1251, D-53002 Bonn, Germany.>

[73] A. L. Spek , Acta Crystallogr., Sect. D: Biol. Crystallogr. 2009, 65, 148–155. 19171970 10.1107/S090744490804362X PMC2631630

[74] Deposition Number(s) 2387283 (for **1**), 2387279 (for **2**), 2387280 (for **3**), 2387282 (for **4**), 2403846 (for **5**), 2387281 (for **10**), 2403845 (for **11**), and 2389609 (for **12**) contain(s) the supplementary crystallographic data for this paper. These data are provided free of charge by the joint Cambridge Crystallographic Data Centre and Fachinformationszentrum Karlsruhe Access Structures service.

