# Supplementary material for: Organotin(IV) Alkoxides, Siloxides, and Related Stannoxanes. Characterisation and Thermogravimetric Studies
Source: ChemistryOpen. 2025 Feb 4;14(8):e202400494. doi: 10.1002/open.202400494 (PMC12368887; doi:10.1002/open.202400494)
Supplement: Supplementary file 1 — Supporting Information [file OPEN-14-e202400494-s001.pdf]

ChemistryOpen

Supporting Information

Organotin(IV) Alkoxides, Siloxides, and Related Stannoxanes. Characterisation and Thermogravimetric Studies

Vlad Penciu, Liliana Bizo, Richard A. Varga, and Adrian-Alexandru Someşan*

Supplementary Information
for

Organotin(IV) alkoxides, siloxides, and related stannoxanes. Characterisation and thermogravimetric studies

Vlad Penciu, Liliana Bizo, Richard A. Varga and Adrian-Alexandru Someşan*

Department of Chemistry, Supramolecular Organic and Organometallic Chemistry Centre,
Faculty of Chemistry and Chemical Engineering, Babeș-Bolyai University,
Cluj-Napoca RO-400028, Romania. Tel: +(40) 264-593833
E-mail: adrian.somesan@ubbcluj.ro

Table of Contents

Contents	*Figures/Tables*	*Pages*
Attempts for synthesis of $L_2SnIOSiPh_3$ (**9**)		p. 3
^{1}H, $^{13}C\{^{1}H\}$ and $^{119}Sn\{^{1}H\}$ NMR spectra of **1-3**	S1 – S9	pp. 4-8
^{29}Si INEPT NMR spectrum of **3**	S10	p. 9
^{1}H, $^{13}C\{^{1}H\}$ and $^{119}Sn\{^{1}H\}$ NMR spectra of **4** and **5**	S11 – S16	pp. 10-13
^{1}H and $^{119}Sn\{^{1}H\}$ NMR spectra of **6**	S17 – S18	p. 14
^{1}H, $^{13}C\{^{1}H\}$ and $^{119}Sn\{^{1}H\}$ NMR spectra of **7**, **8**,**10, 11**	S19 – S30	pp. 15-22
^{1}H, $^{13}C\{^{1}H\}$, $^{11}B\{^{1}H\}$ and $^{119}Sn\{^{1}H\}$ NMR spectrum of **12**	S31 – S34	pp. 23-24
$^{119}Sn\{^{1}H\}$ NMR stacked spectra for **1-10**	S35 – S36	p. 25
X-ray crystallographic data for compounds **1-5**, **10-12**	Table S1, S2	pp. 26-27
Molecular structure and data of the two distinct molecules in **1**	S37 / Table S3	p. 28
Representation of the four isomers in the crystal of **1**	S38 – S39	p. 29
Representation of the two isomers in the crystal of **2**	S40	p. 30
Representation of the two isomers in the crystal of **3** and **4**	S41 – S42	p. 31
Molecular structure and data of **5**	S43 / Table S4	p. 32
Representation of the two isomers in the crystal of **5**	S44	p. 33
Molecular structure and data of the two distinct molecules in **10**	S45 / Table S5	p. 34
Representation of the four isomers in the crystal of **10**	S46 – S47	p. 35
Representation of the two isomers in the crystal of **11**	S48 / Table S6	p. 36
Representation of the two isomers in the crystal of **12**	S49	p. 37
Reaction pathway for the formation of **12**	S50	p. 37
Molecular structure and data of **12**	S51 / Table S7	p. 38
HRMS spectra of **1-3**, **5**, **8** and **10-12**	S52 – S58	pp. 39-42
TGA curves of **3-5**, **8** and **12**	S59 – S63	pp. 43-45
References / Author Contributions		p. 46

Attempts for synthesis of $L_2SnIOSiPh_3$ (9):
(**Route 1)** Compounds [2-{$(CH_2O)_2CH$}C_6H_4]$_2SnI_2$ (**2**) (0.139 g, 0.21 mmol) and [2-{$(CH_2O)_2CH$}C_6H_4]$_2Sn(OSiPh_3)_2$ (**10**) (0.200 g, 0.21 mmol) were mixed and dissolved in toluene (30 mL). After 20 h of reflux, toluene was removed using a rotary evaporator, then pentane (30 mL) was added over the resulting oil and stirred for 30 minutes. The obtained precipitate was filtered and dried, resulting in 0.221 g (65%) of a yellow solid. The reaction is a chemical equilibrium; hence the title compound is obtained in a mixture with the reactants.
(Route 2) A solution of Ph_3SiONa (0.146 g, 0.49 mmol) in THF (6 mL) was added dropwise to a solution of **2** (0.327 g, 0.49 mmol) in THF (20 mL), at 0 °C, and the mixture was stirred overnight. The solvent was removed in a rotary evaporator and toluene (15 mL) was added. The obtained suspension was filtered, and toluene was evaporated, resulting in 0.300 g of a pale-yellow solid (60%). The title compound is obtained in mixture with the starting material **2** and the disubstituted product, $L_2Sn(OSiPh_3)_2$ (**10**). Attempts to perform the reaction under inert atmosphere or to decrease the reaction temperature to −78 °C resulted in the same equilibrium. **$^{119}Sn\{^1H\}$ NMR** ($CDCl_3$, 223.74 MHz, 22 °C) δ (ppm): −297.8. **$^{119}Sn\{^1H\}$ NMR** (C_6D_6, 223.74 MHz, 21 °C) δ (ppm): −300.4.

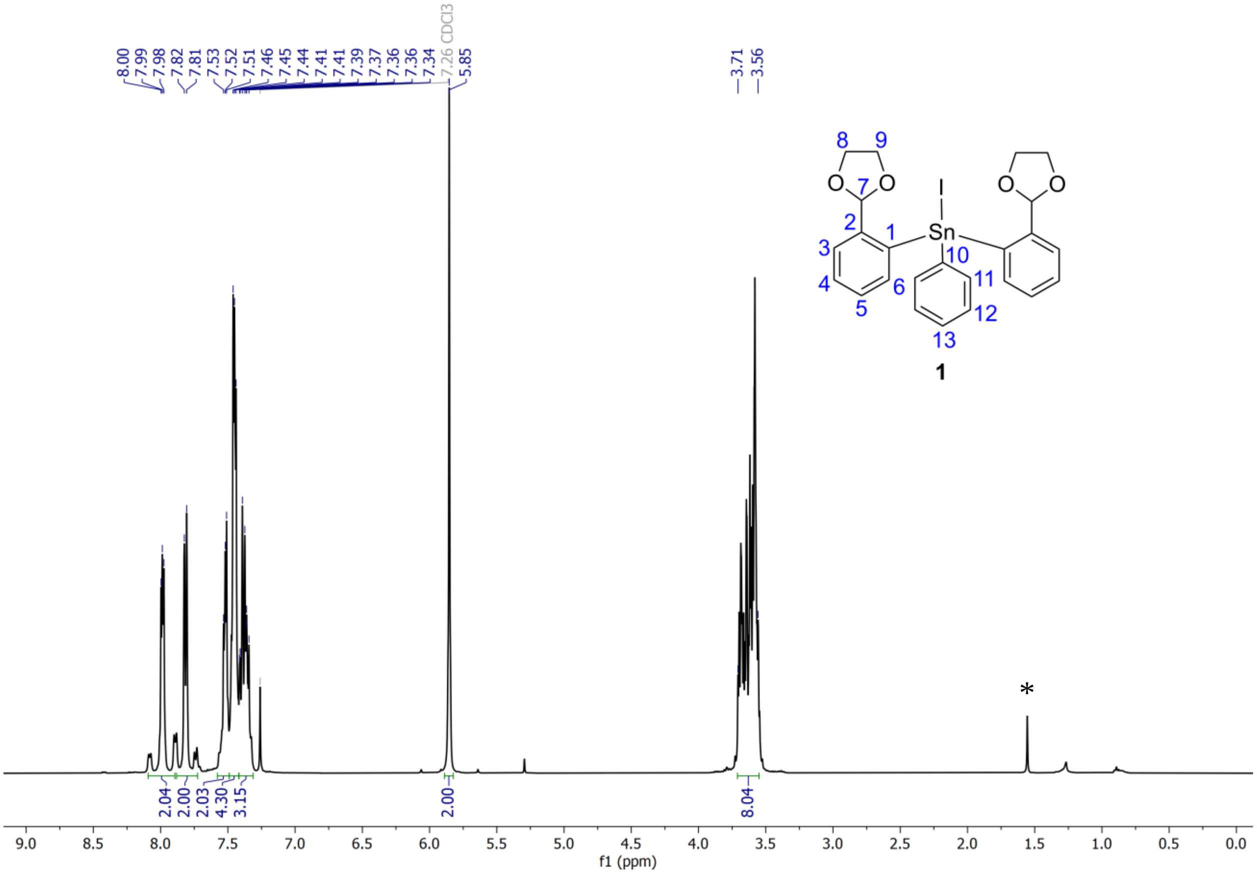

Figure S1. ^{1}H NMR spectrum (400.13 MHz, 21 °C, $CDCl_3$) of L_2PhSnI (**1**). Traces of water are indicated by *.

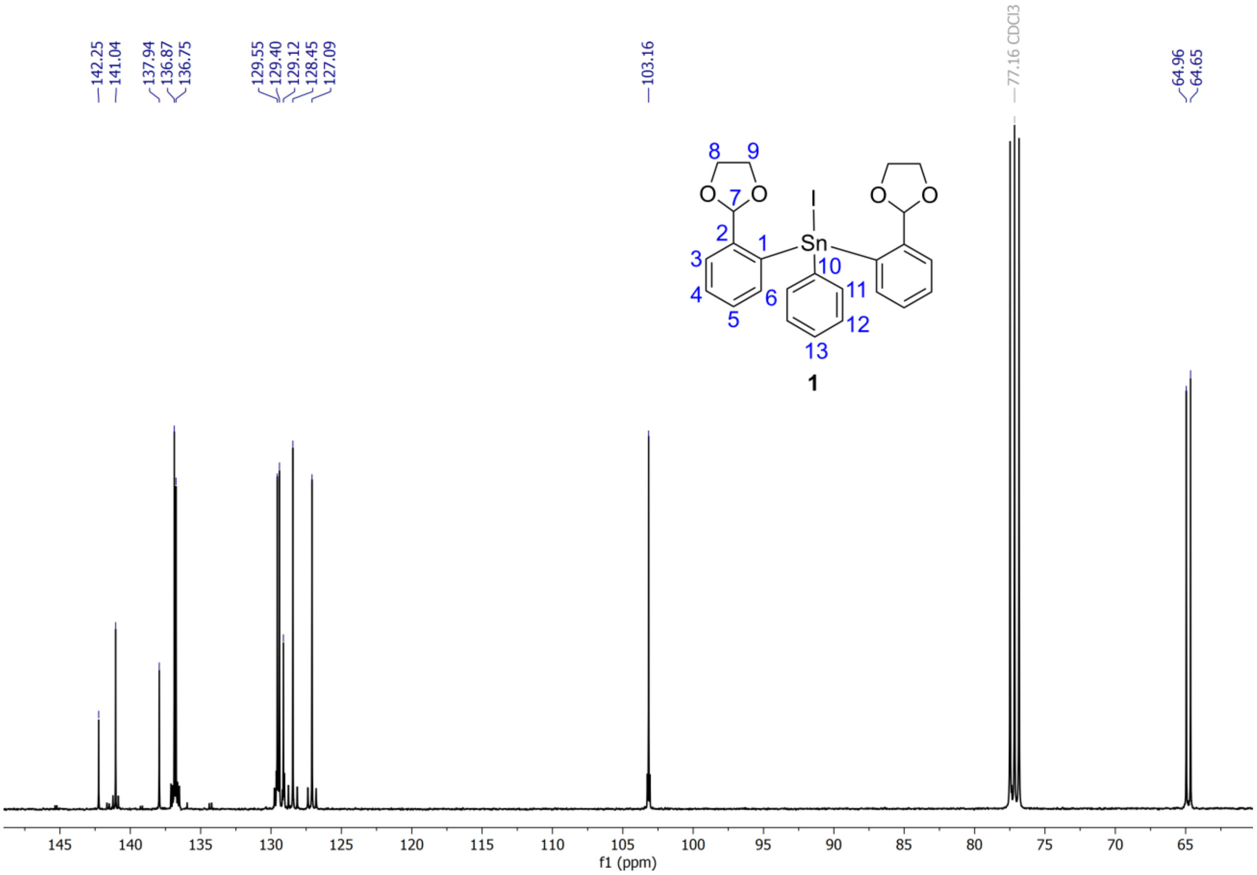

Figure S2. $^{13}C\{^{1}H\}$ NMR spectrum (100.62 MHz, 21 °C, $CDCl_3$) of L_2PhSnI (**1**).

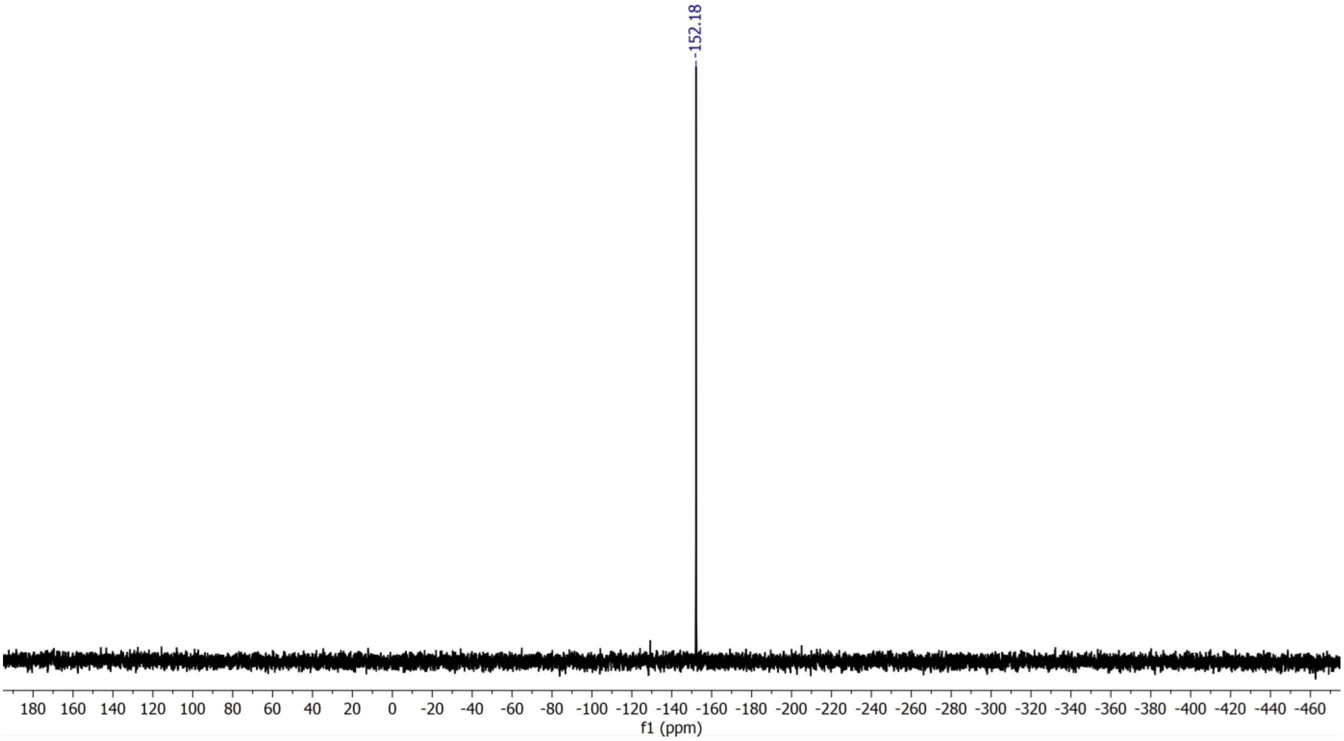

Figure S3. $^{119}Sn\{^1H\}$ NMR spectrum (149.19 MHz, 21 °C, $CDCl_3$) of L_2PhSnI (**1**).

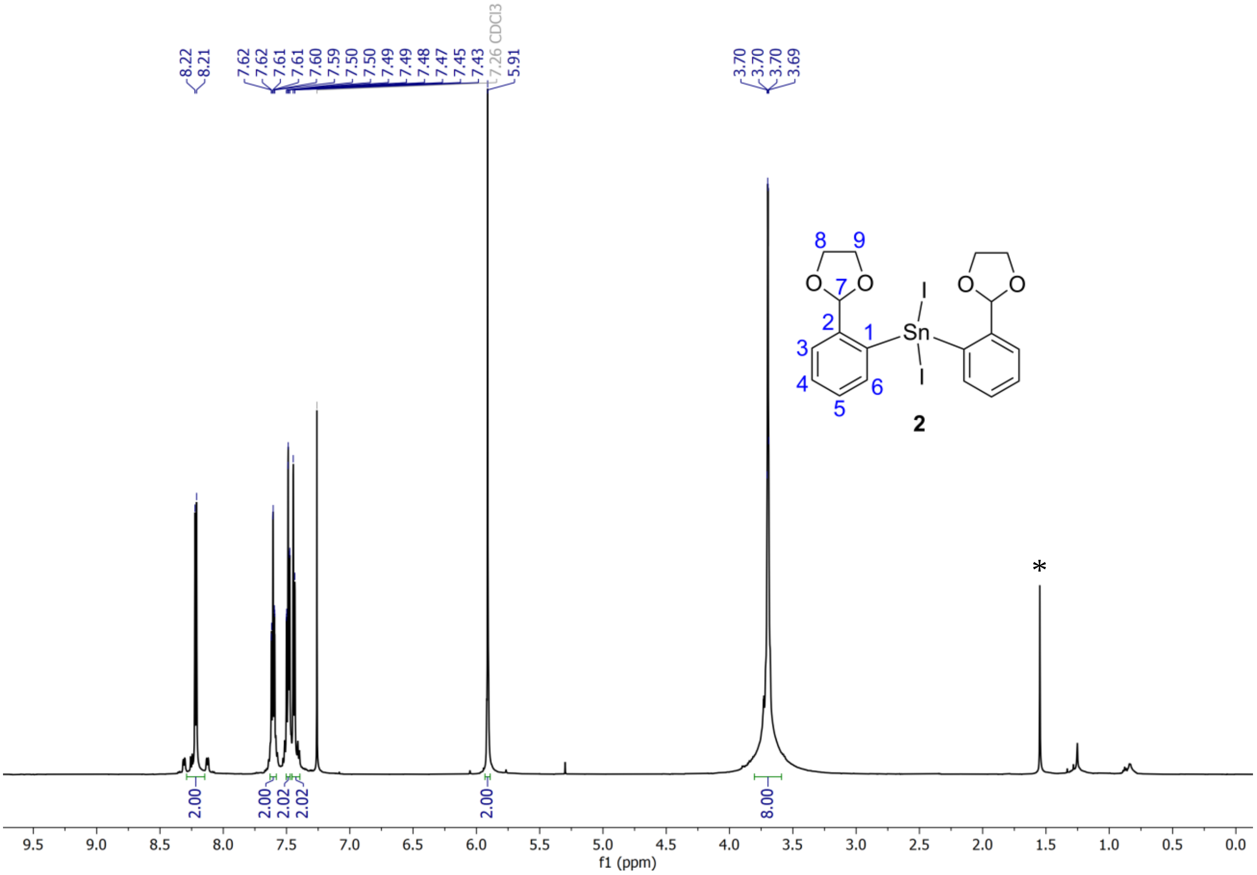

Figure S4. ^{1}H NMR spectrum (600.13 MHz, 21 °C, $CDCl_3$) of L_2SnI_2 (**2**). Traces of water are indicated by *.

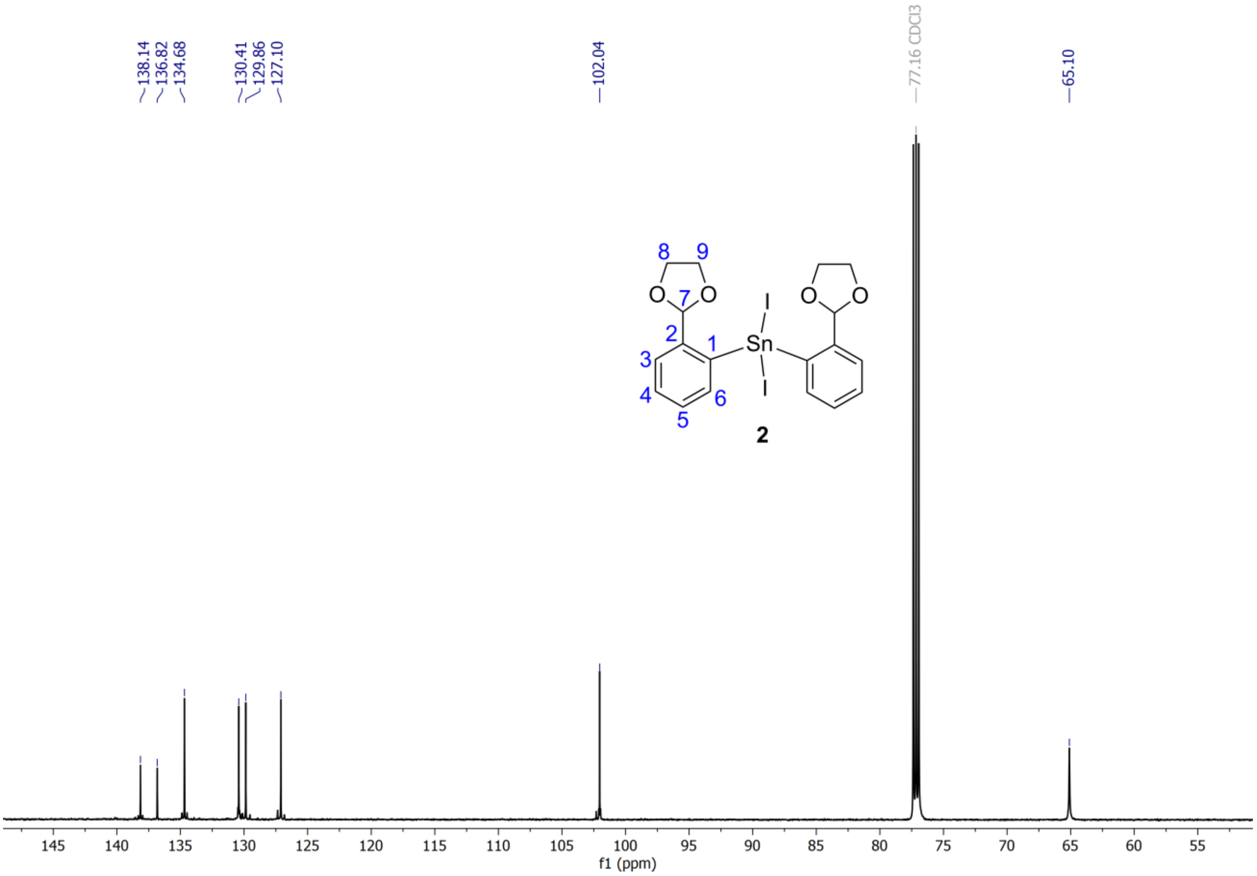

Figure S5. $^{13}C\{^{1}H\}$ NMR spectrum (150.92 MHz, 21 °C, $CDCl_3$) of L_2SnI_2 (**2**).

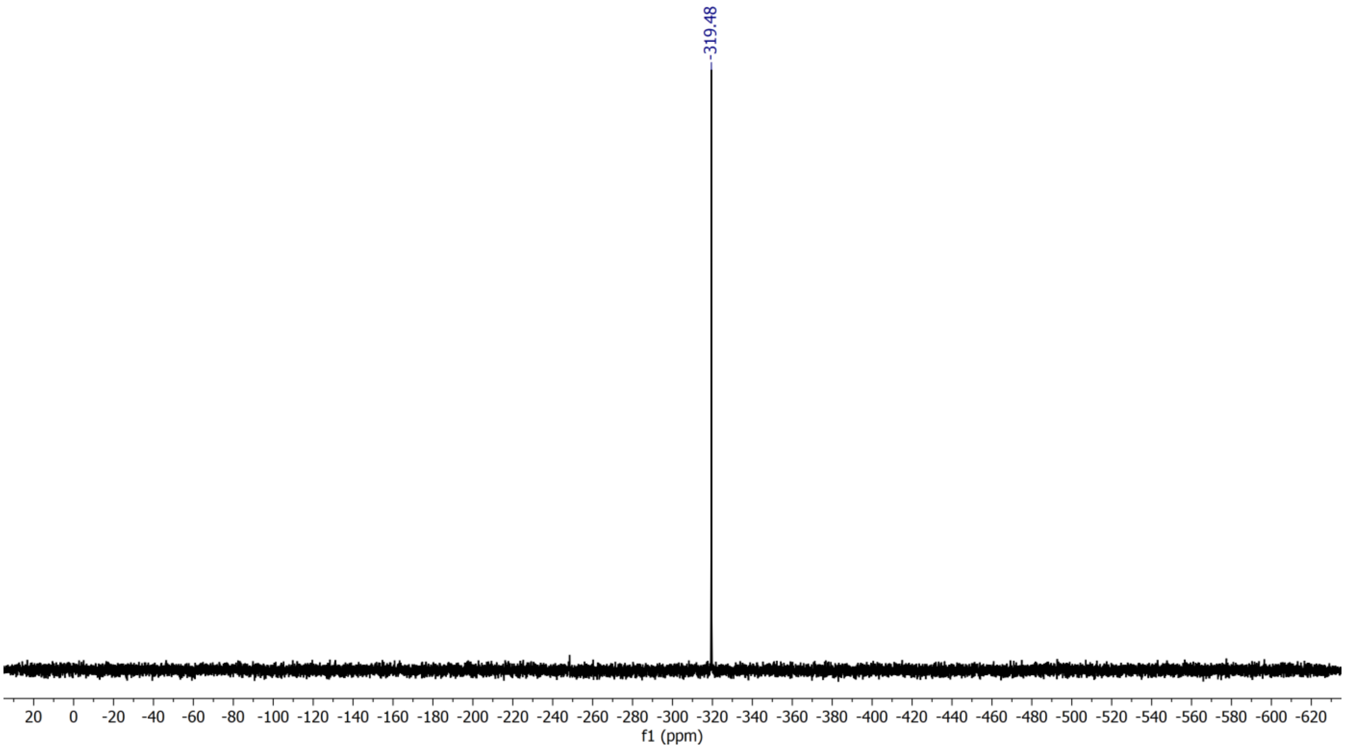

Figure S6. $^{119}Sn\{^1H\}$ NMR spectrum (223.73 MHz, 21 °C, $CDCl_3$) of L_2SnI_2 (**2**).

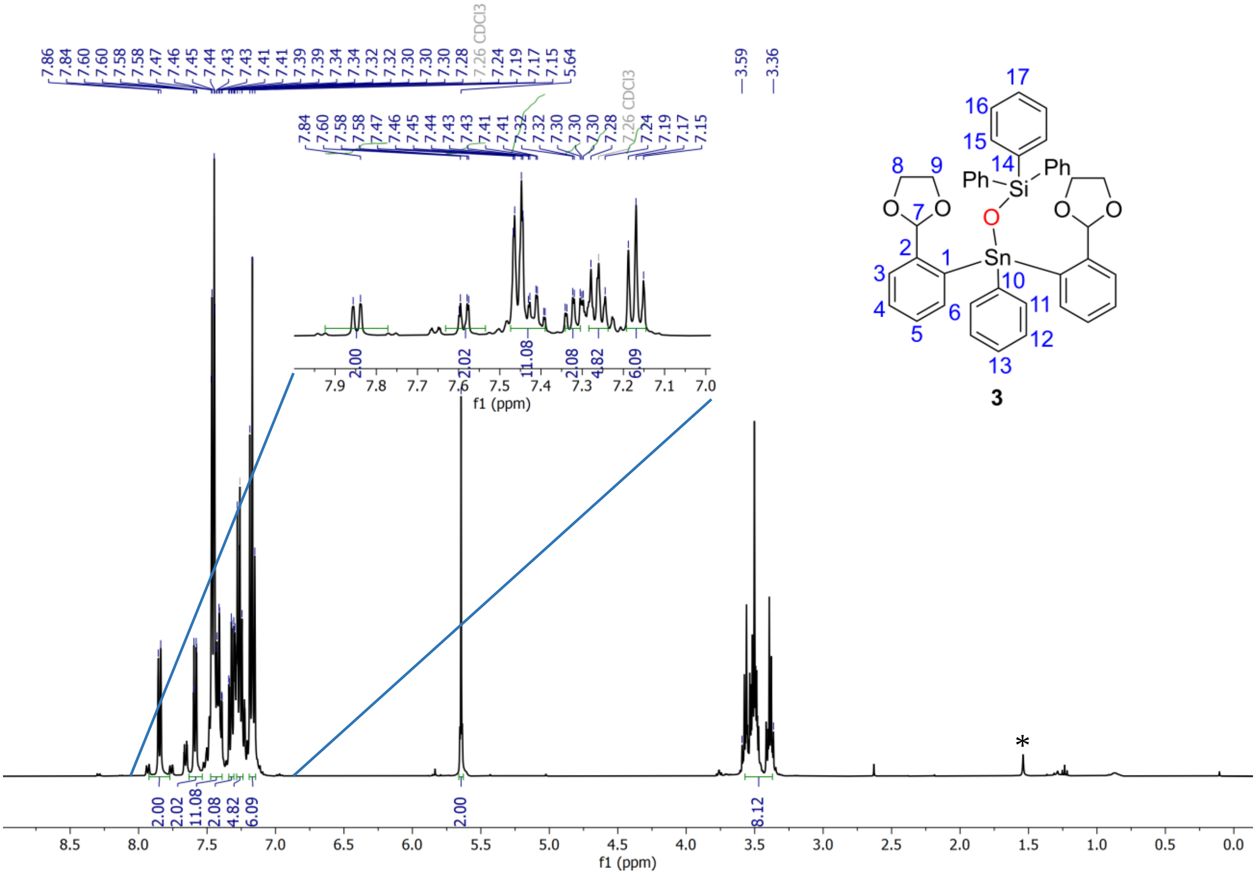

Figure S7. ^{1}H NMR spectrum (400.13 MHz, 20 °C, $CDCl_3$) of $L_2PhSnOSiPh_3$ (**3**). Traces of water are indicated by *

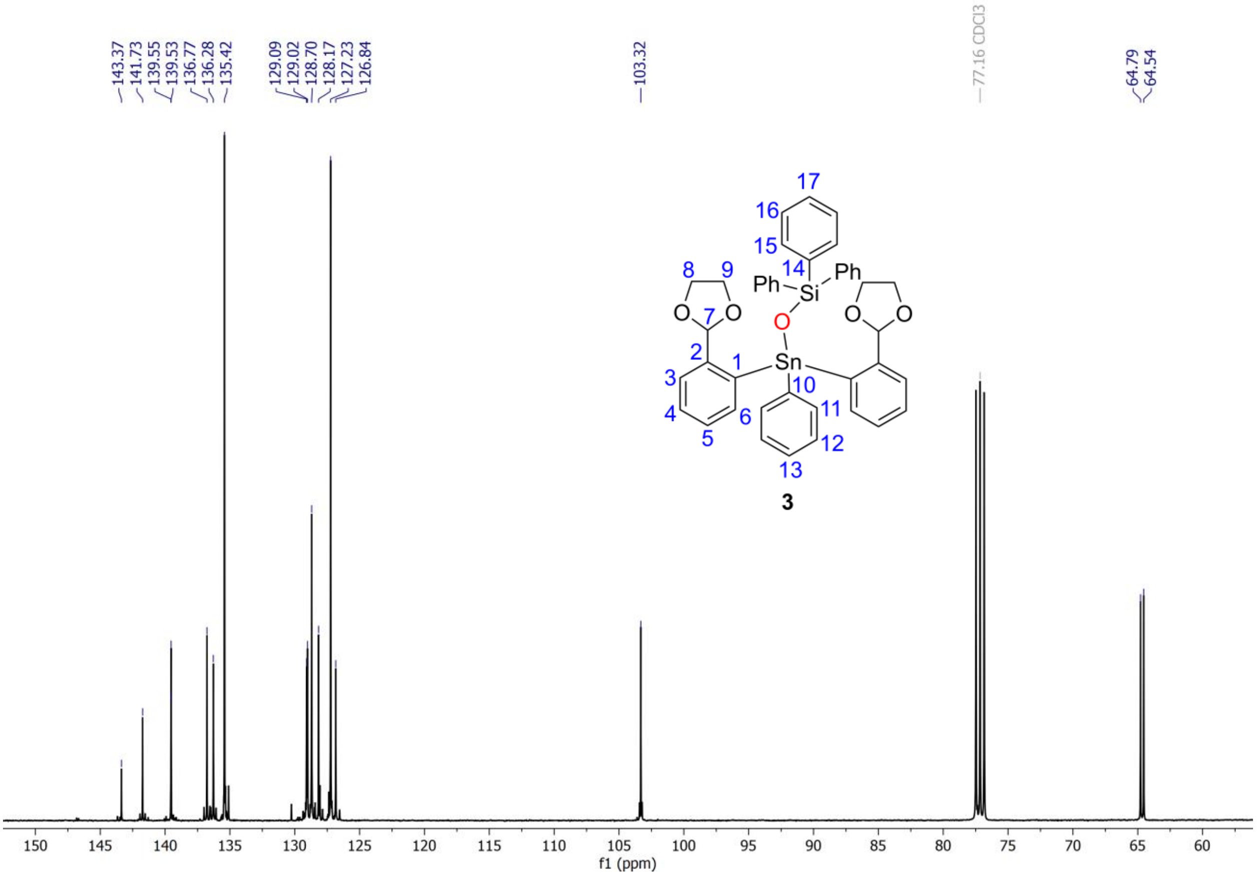

Figure S8. $^{13}C\{^{1}H\}$ NMR spectrum (100.62 MHz, 20 °C, $CDCl_3$) of $L_2PhSnOSiPh_3$ (**3**).

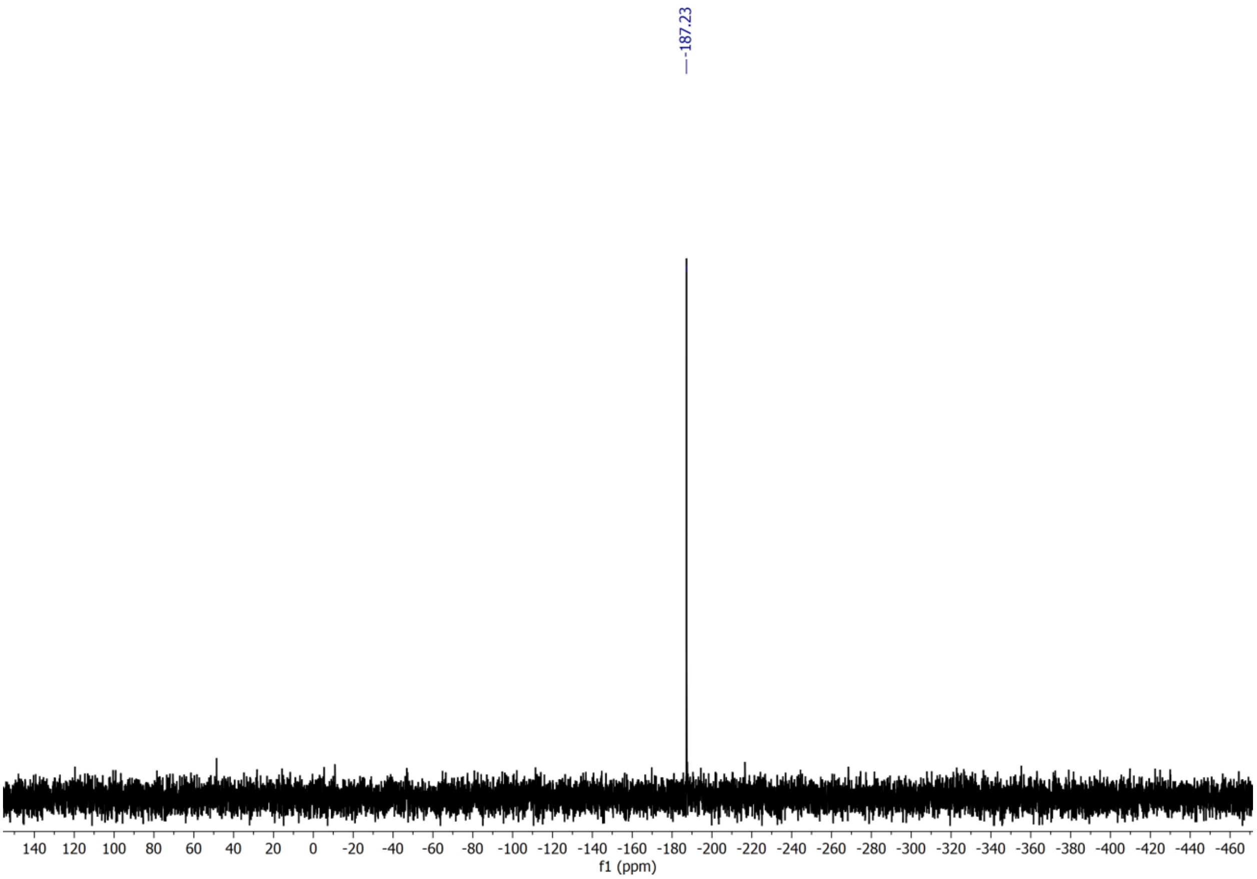

Figure S9. $^{119}Sn\{^{1}H\}$ NMR spectrum (149.19 MHz, 21 °C, $CDCl_3$) of $L_2PhSnOSiPh_3$ (**3**).

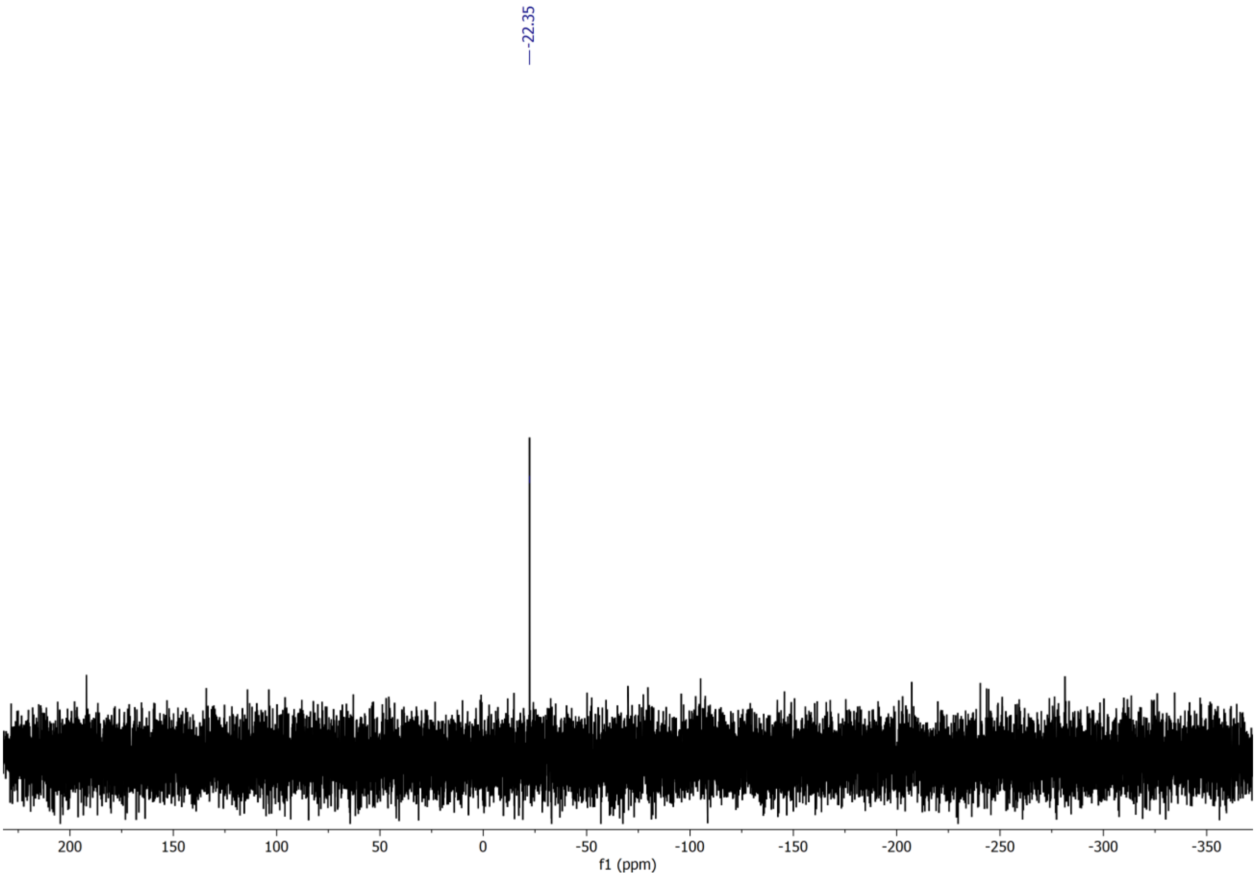

Figure S10. ^{29}Si INEPT NMR spectrum (79.49 MHz, 20 °C, $CDCl_3$) of $L_2PhSnOSiPh_3$ (**3**).

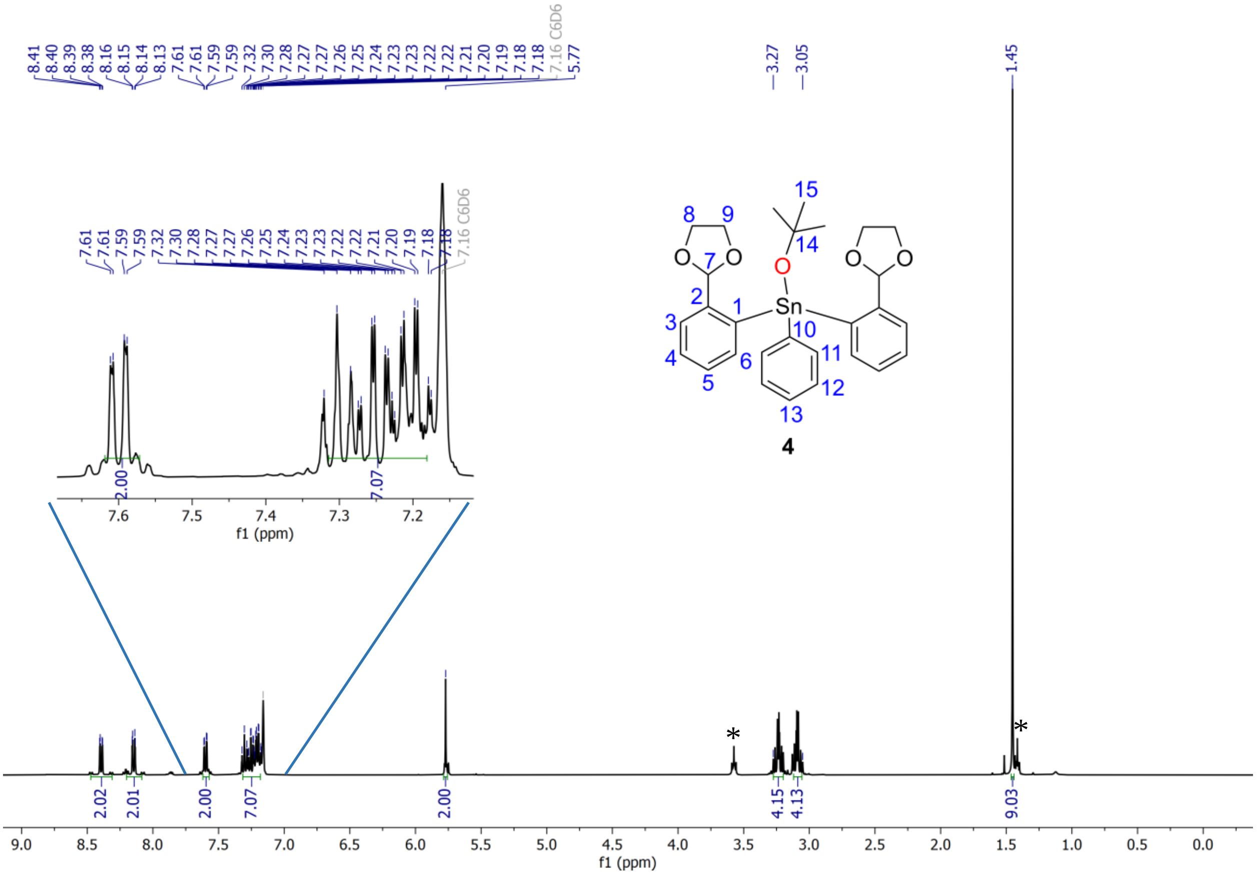

Figure S11. ^{1}H NMR spectrum (400.13 MHz, 22 °C, C_6D_6) of L_2PhSnO^tBu (**4**). Residual THF indicated by *.

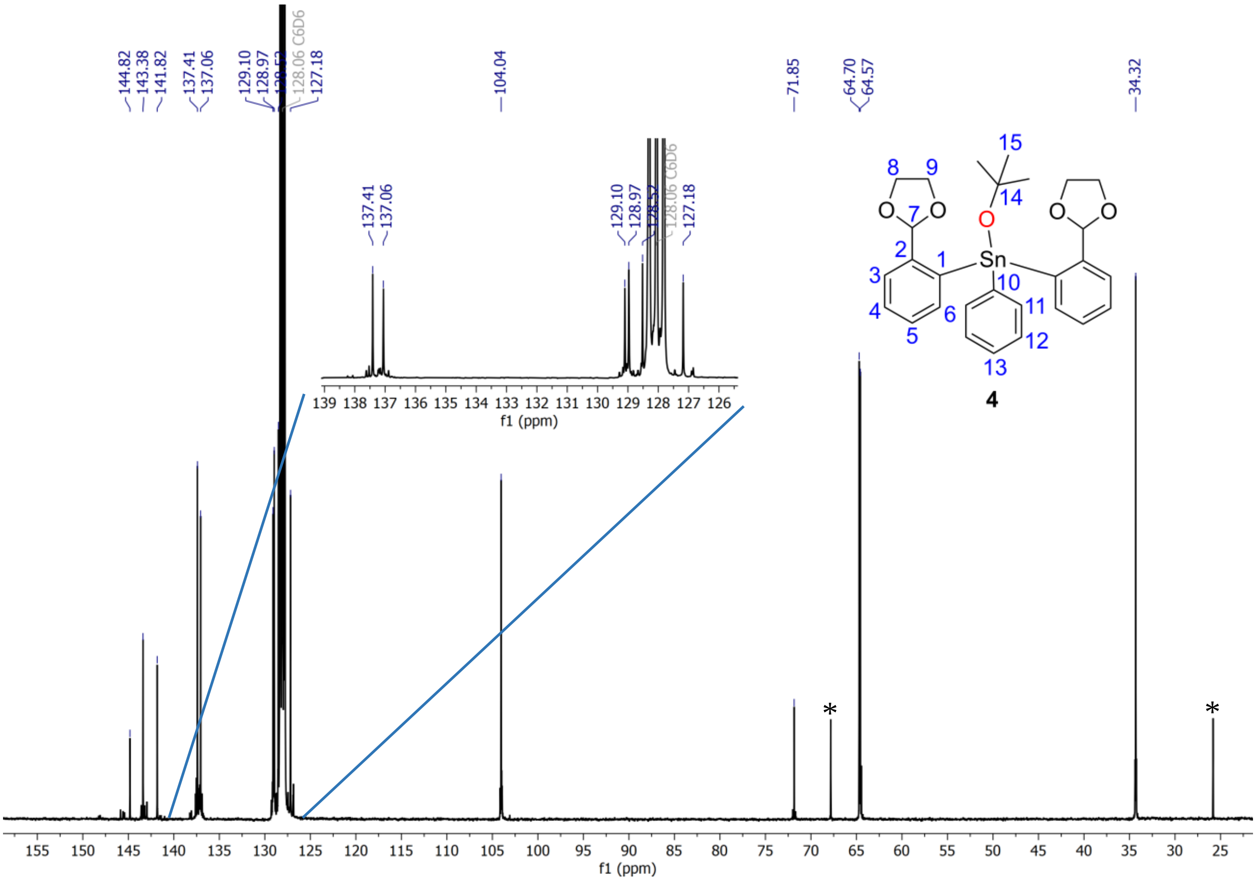

Figure S12. $^{13}C\{^1H\}$ NMR spectrum (100.62 MHz, 22 °C, C_6D_6) of L_2PhSnO^tBu (**4**). Residual THF indicated by *.

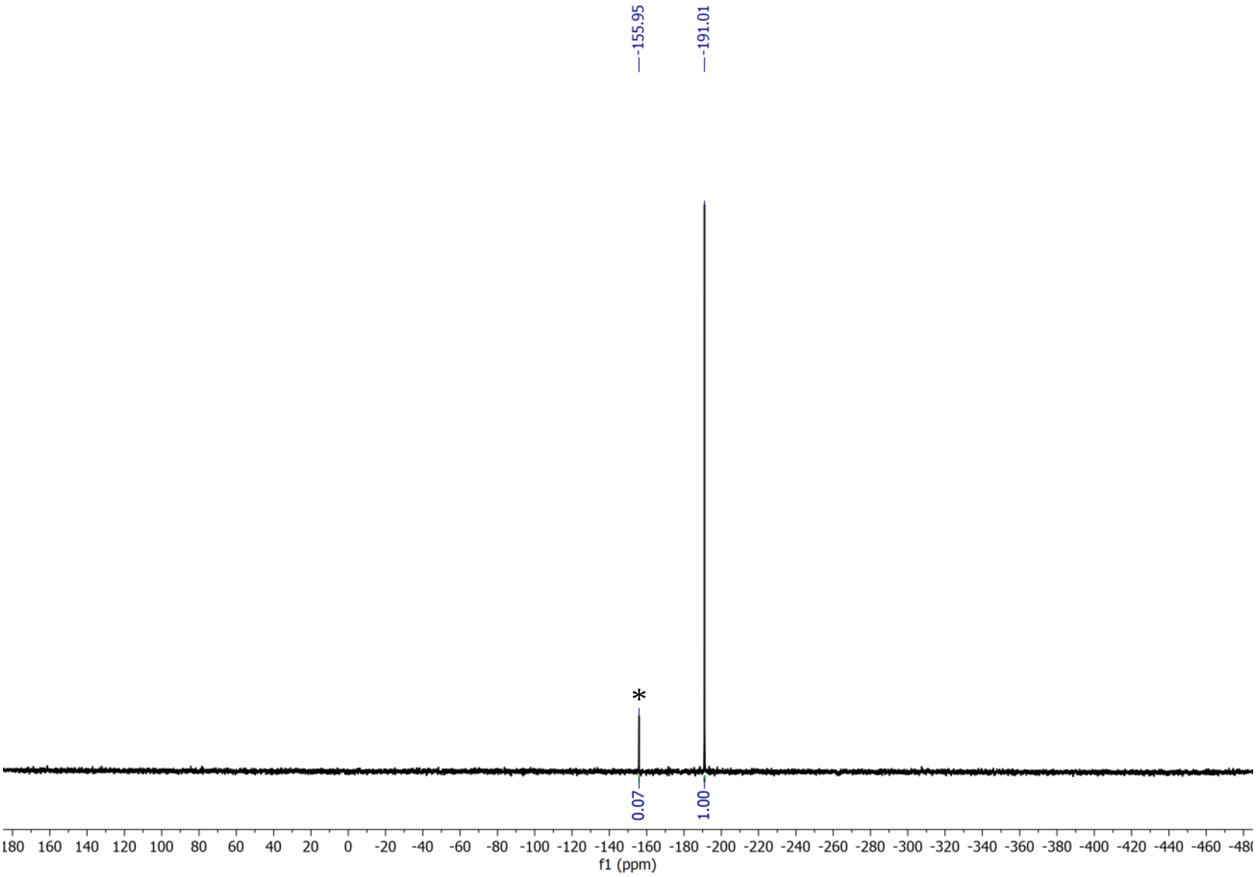

Figure S13. ^{119}Sn{^{1}H} NMR spectrum (149.19 MHz, 21 °C, C_6D_6) of L_2PhSnO^{t}Bu (**4**). Traces of $(L_2PhSn)_2O$ (**5**) indicated by *.

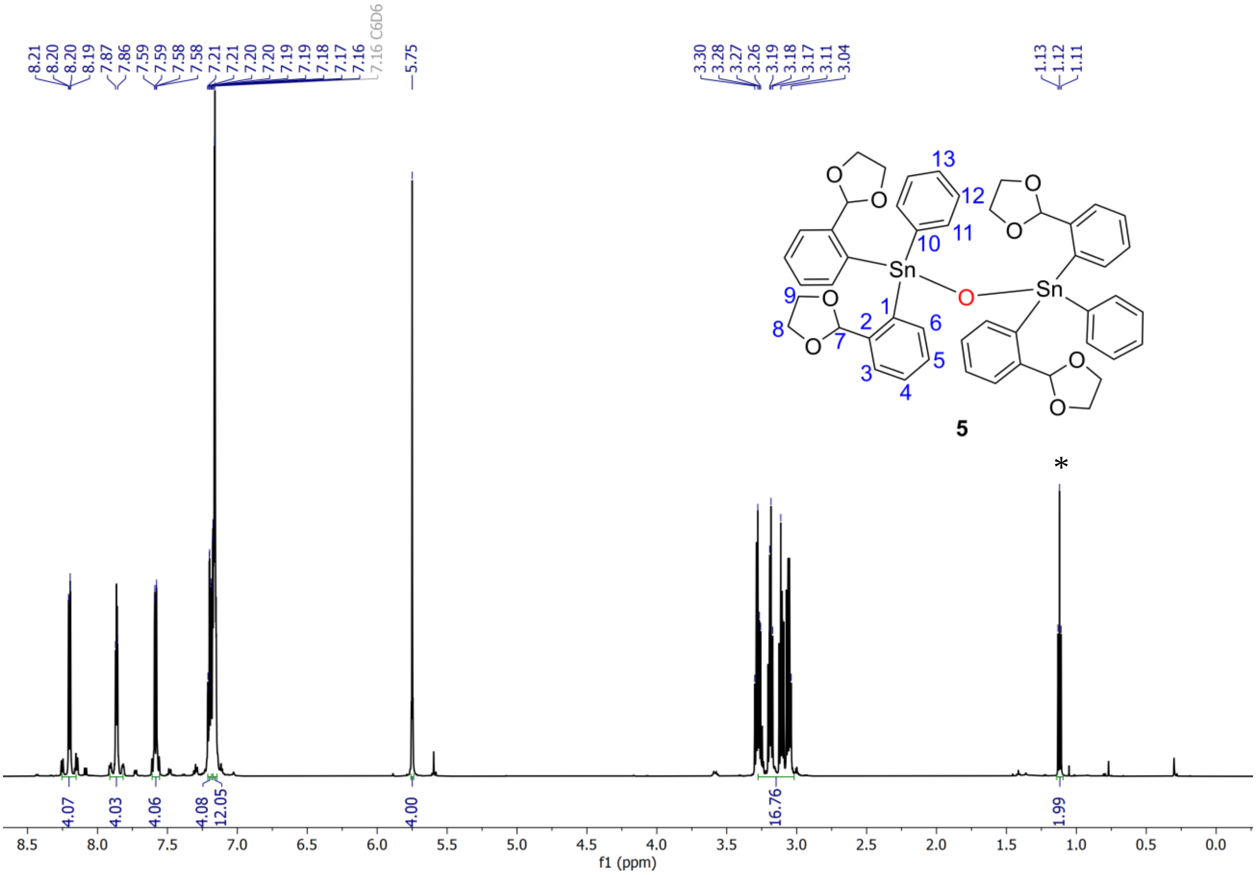

Figure S14. ^{1}H NMR spectrum (600.13 MHz, 21 °C, C_6D_6) of $(L_2PhSn)_2O$ (**5**). Crystallisation Et_2O indicated by *.

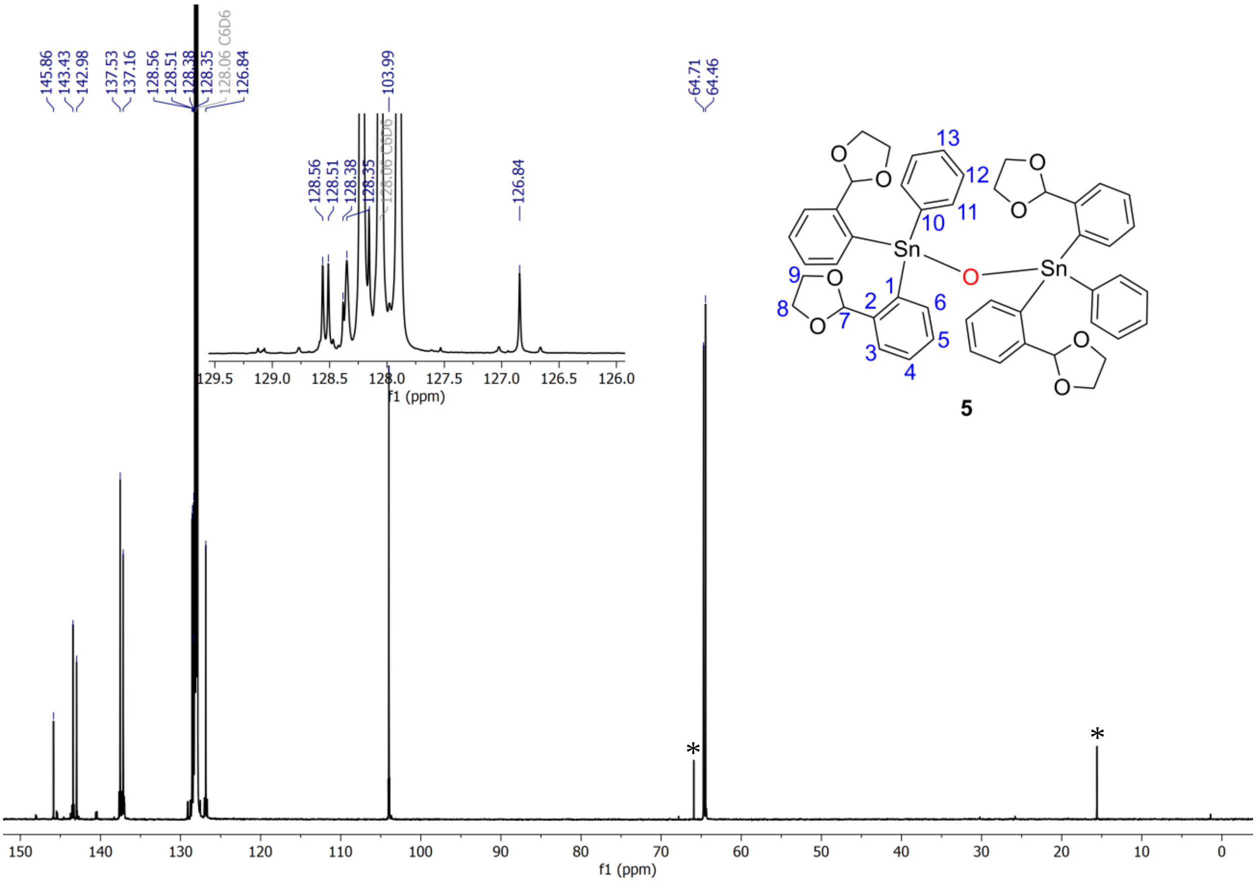

Figure S15. $^{13}C\{^{1}H\}$ NMR spectrum (150.92 MHz, 21 °C, C_6D_6) of $(L_2PhSn)_2O$ (**5**). Crystallisation Et_2O indicated by *.

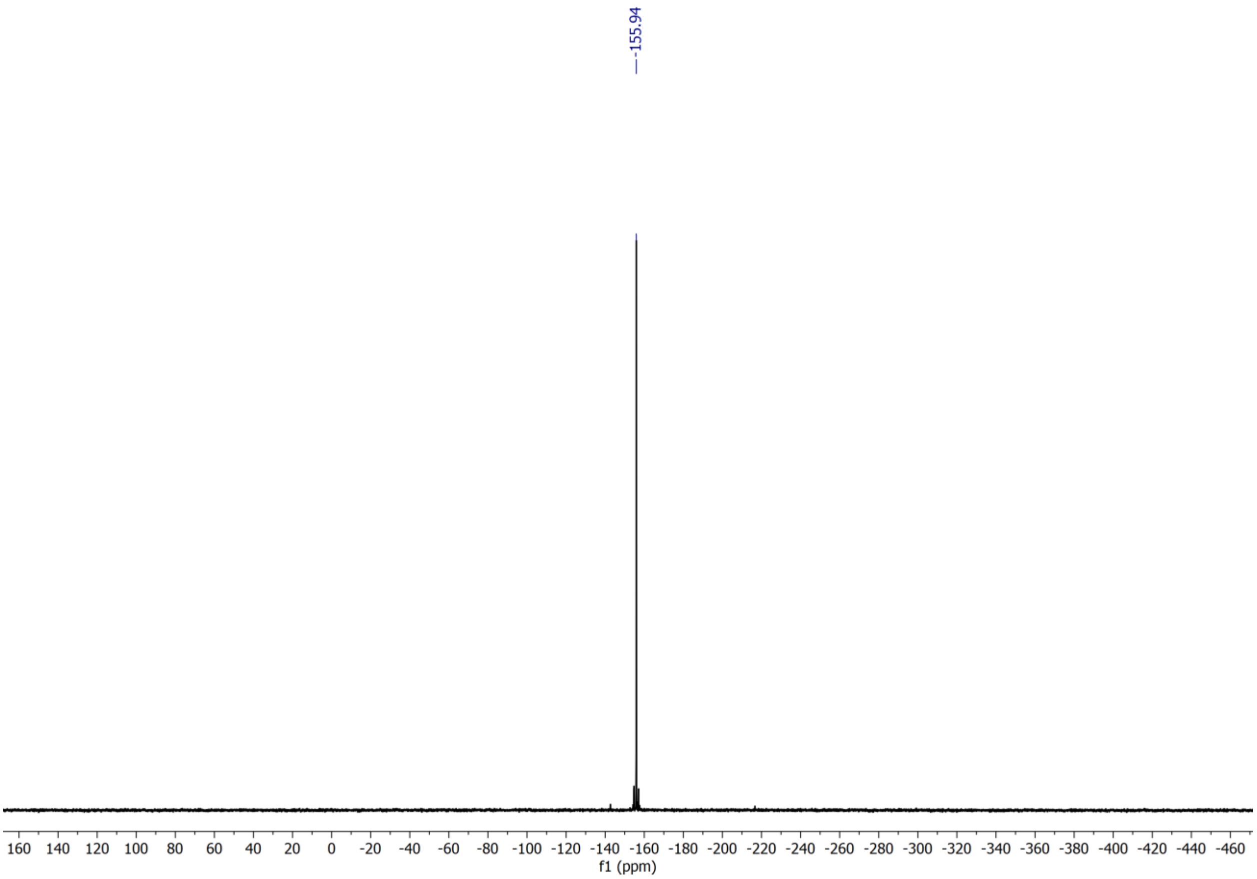

Figure S16. $^{119}Sn\{^{1}H\}$ NMR spectrum (223.76 MHz, 21 °C, C_6D_6) of $(L_2PhSn)_2O$ (**5**).

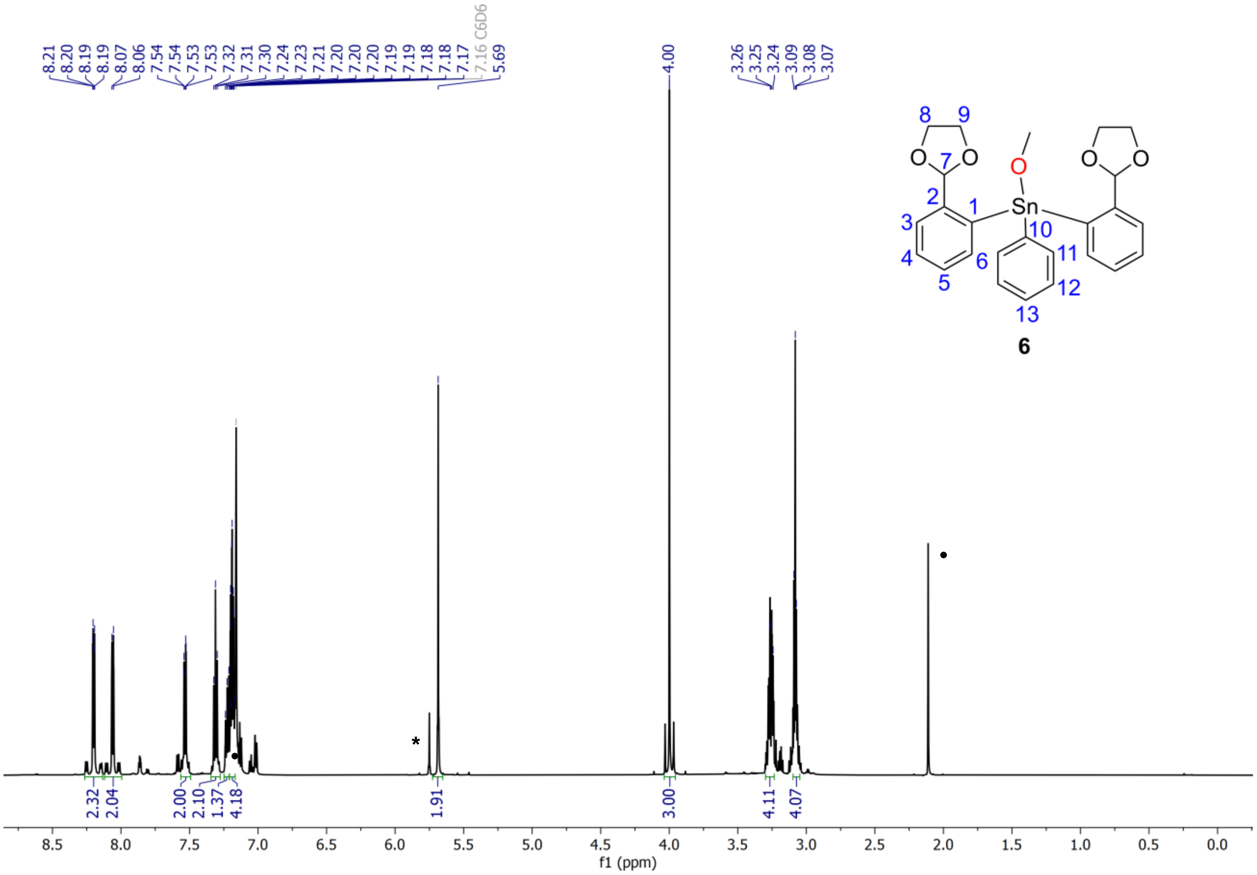

Figure S17. ^{1}H NMR spectrum (600.13 MHz, 21 °C, C_6D_6) of $L_2PhSnOMe$ (**6**). Decomposition product $(L_2PhSn)_2O$ (**5**) indicated by *. Residual toluene indicated by •.

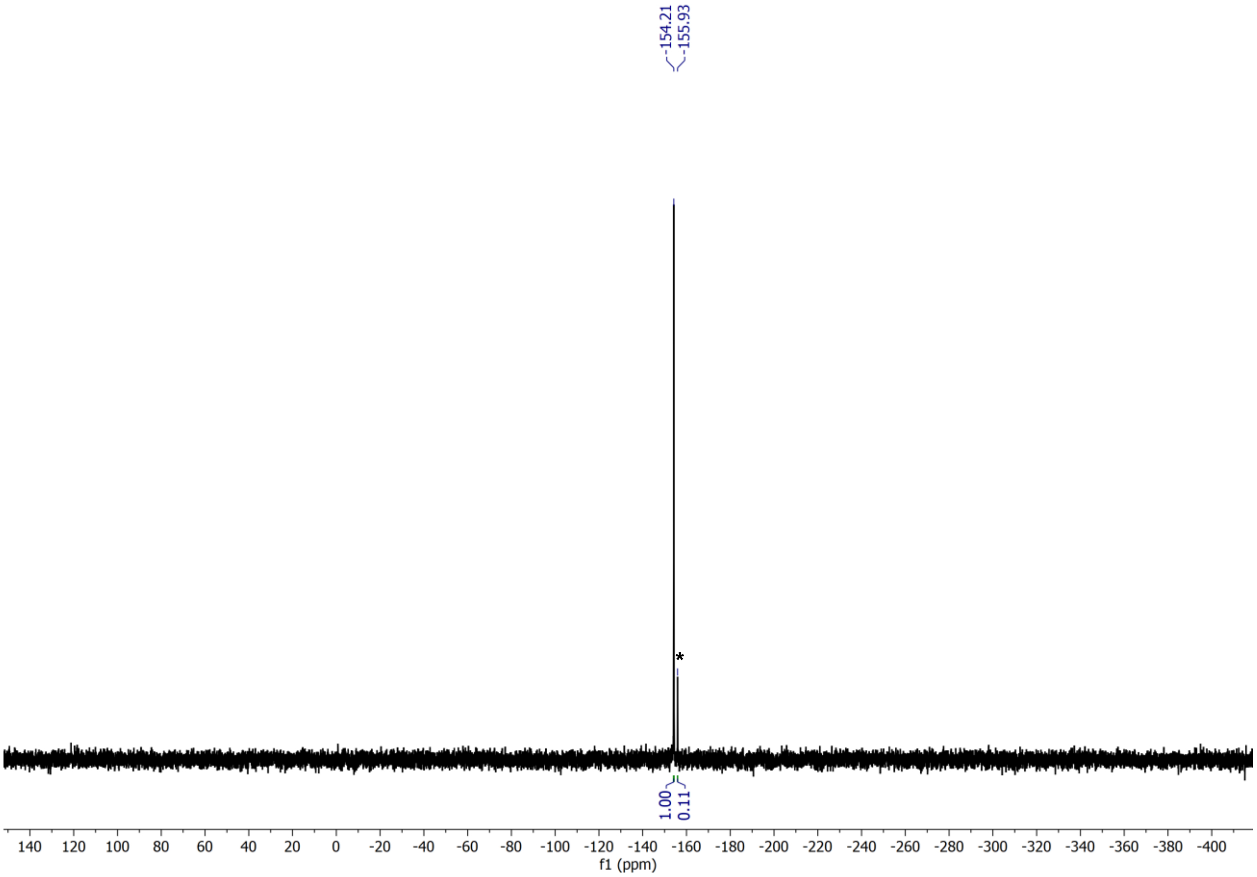

Figure S18. $^{119}Sn\{^{1}H\}$ NMR spectrum (223.77 MHz, 22 °C, C_6D_6) of $L_2PhSnOMe$ (**6**). Decomposition product $(L_2PhSn)_2O$ (**5**) indicated by *.

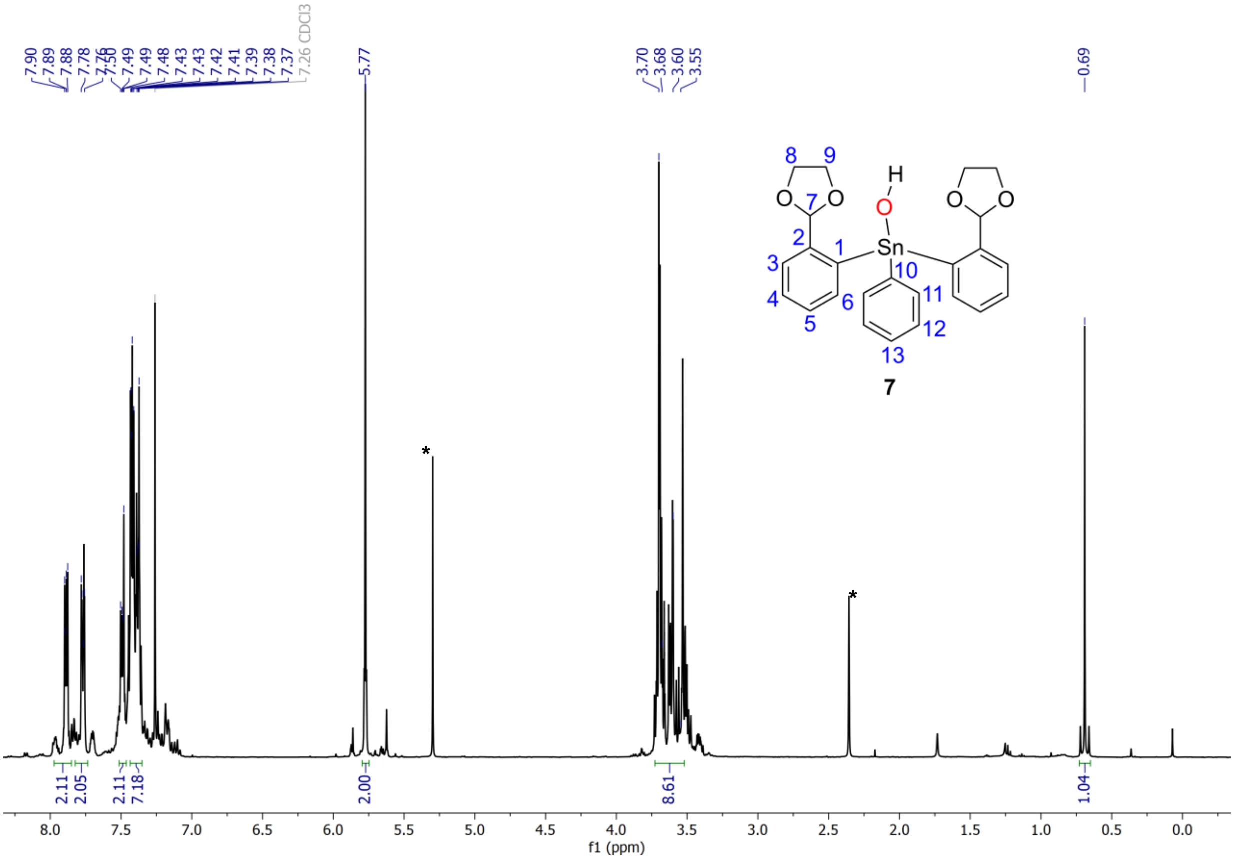

Figure S19. ^{1}H NMR spectrum (400.13 MHz, 20 °C, $CDCl_3$) of $L_2PhSnOH$ (**7**). Traces of solvents (CH_2Cl_2 and toluene) are indicated by *.

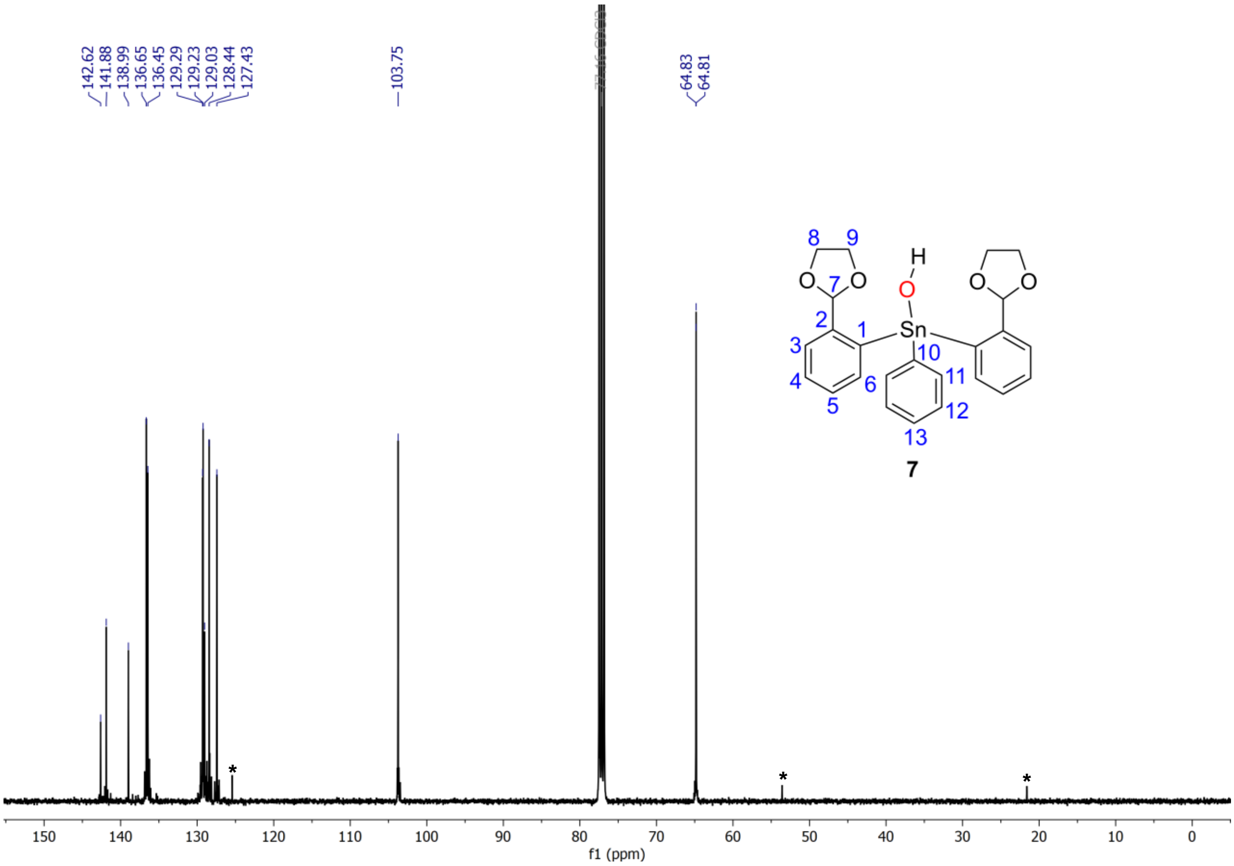

Figure S20. $^{13}C\{^{1}H\}$ NMR spectrum (100.62 MHz, 20 °C, $CDCl_3$) of $L_2PhSnOH$ (**7**). Traces of solvents (CH_2Cl_2 and toluene) are indicated by *.

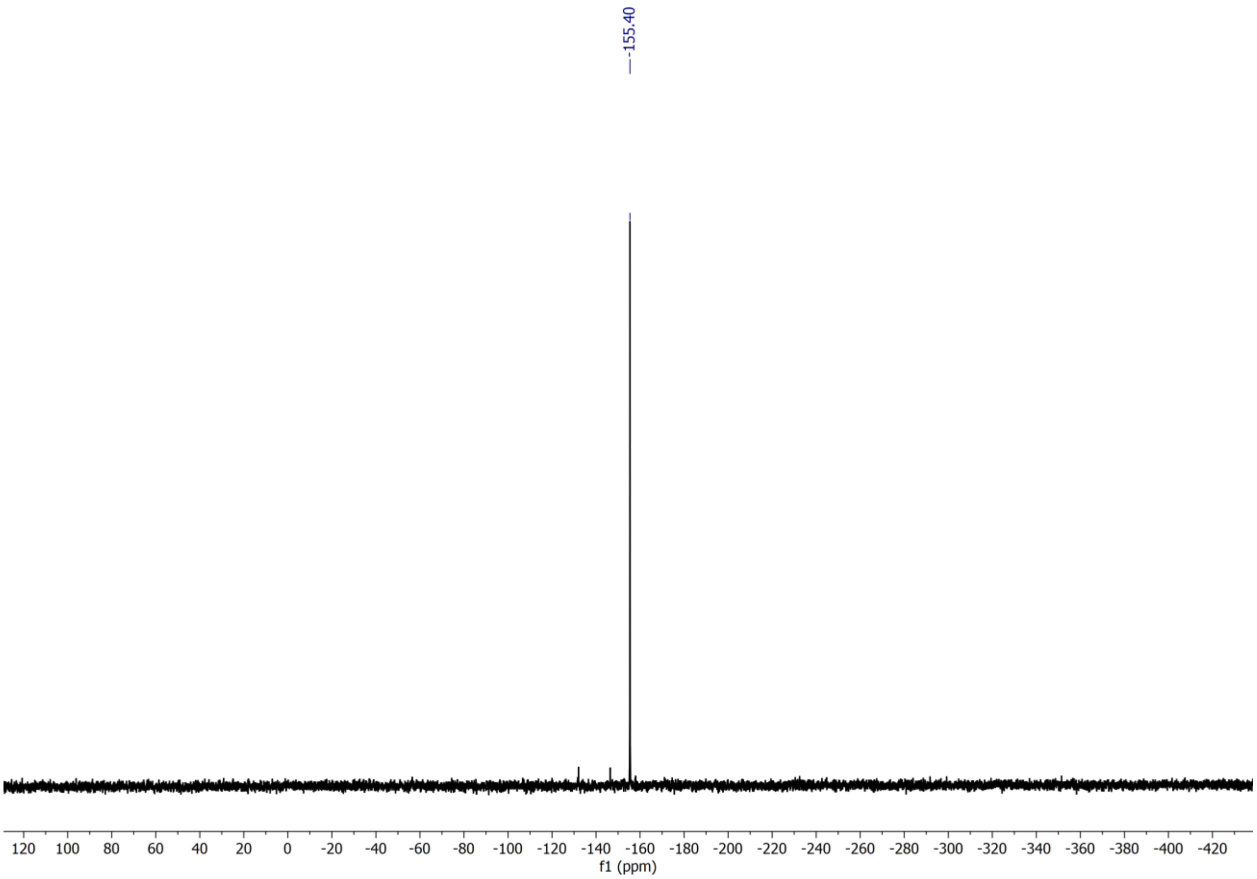

Figure S21. $^{119}Sn\{^1H\}$ NMR spectrum (149.19 MHz, 20 °C, $CDCl_3$) of $L_2PhSnOH$ (**7**).

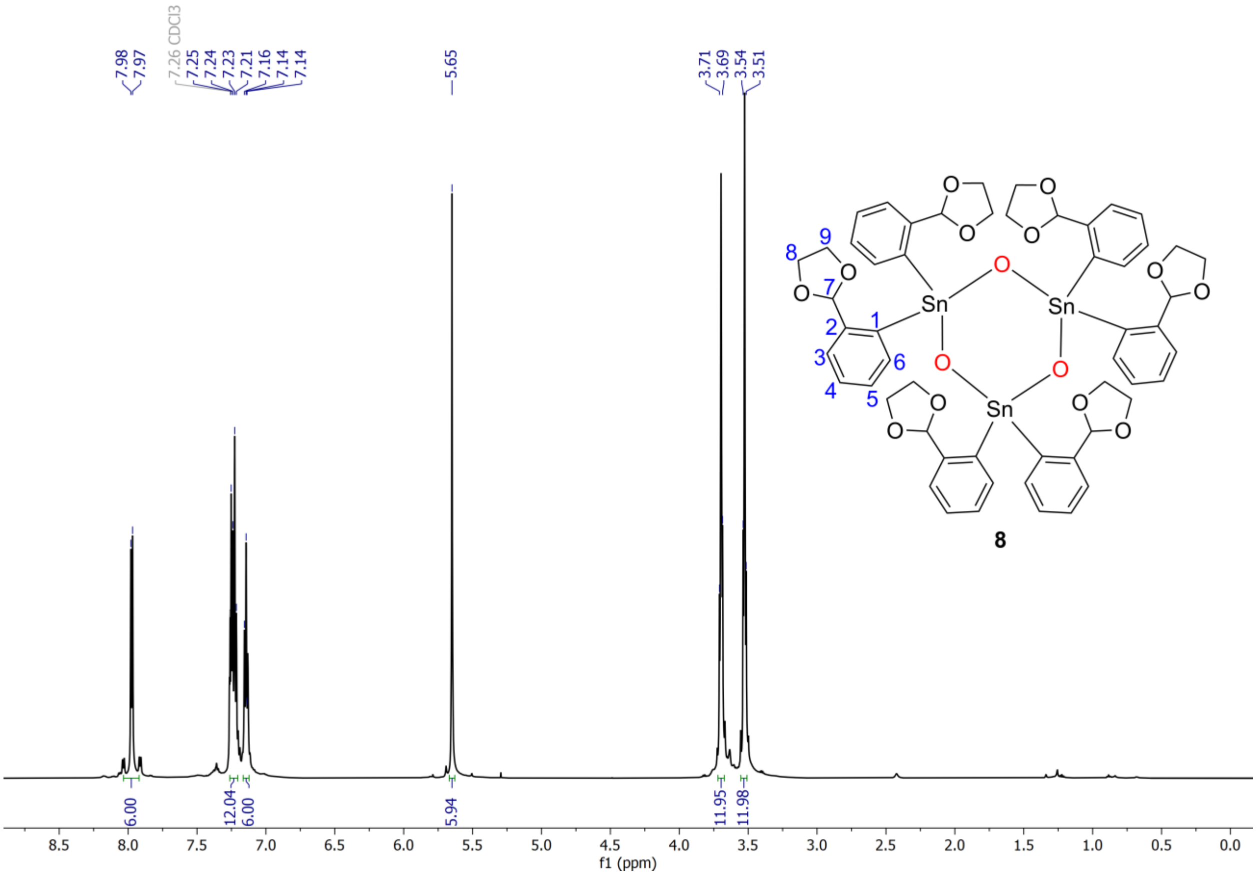

Figure S22. ^{1}H NMR spectrum (600.13 MHz, 21 °C, $CDCl_3$) of $(L_2SnO)_3$ (**8**).

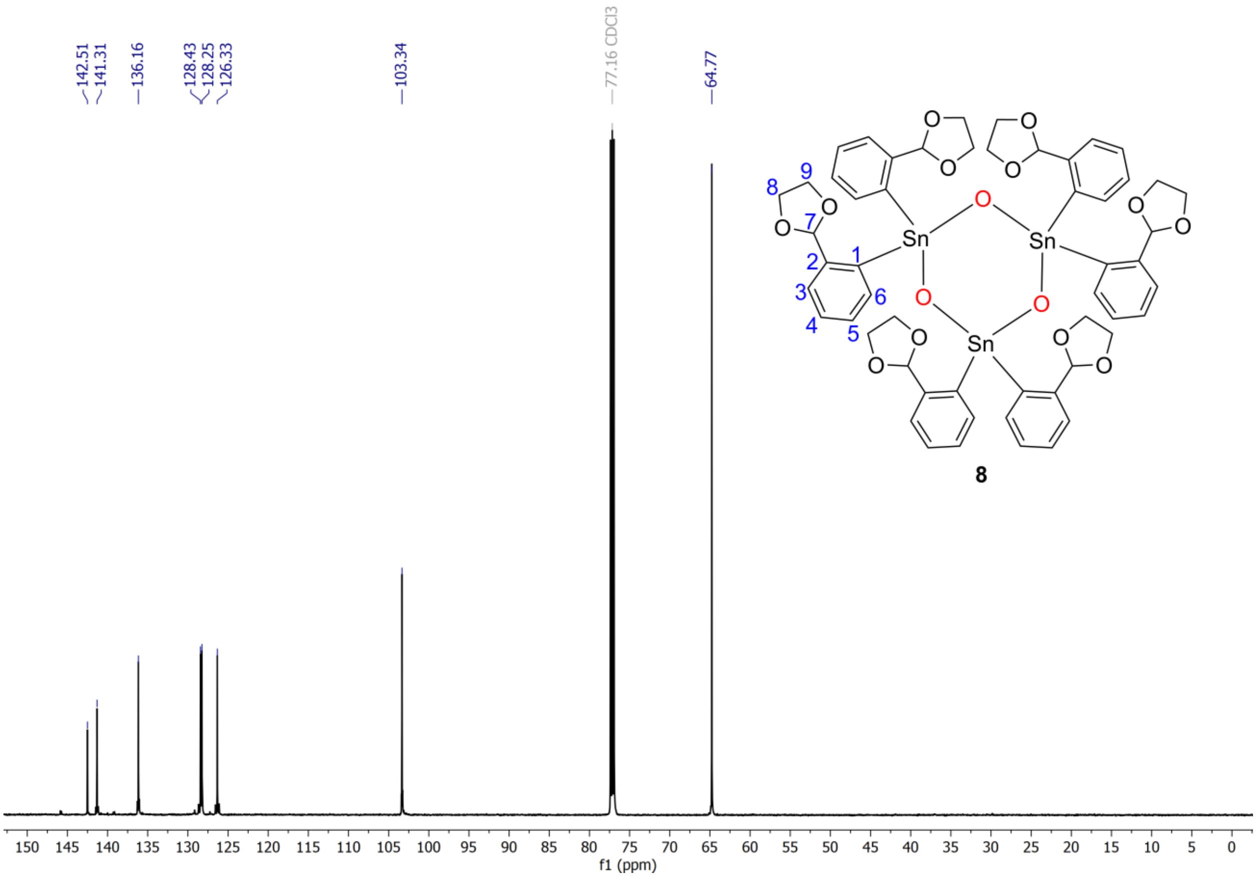

Figure S23. $^{13}C\{^{1}H\}$ NMR spectrum (150.92 MHz, 21 °C, $CDCl_3$) of $(L_2SnO)_3$ (**8**).

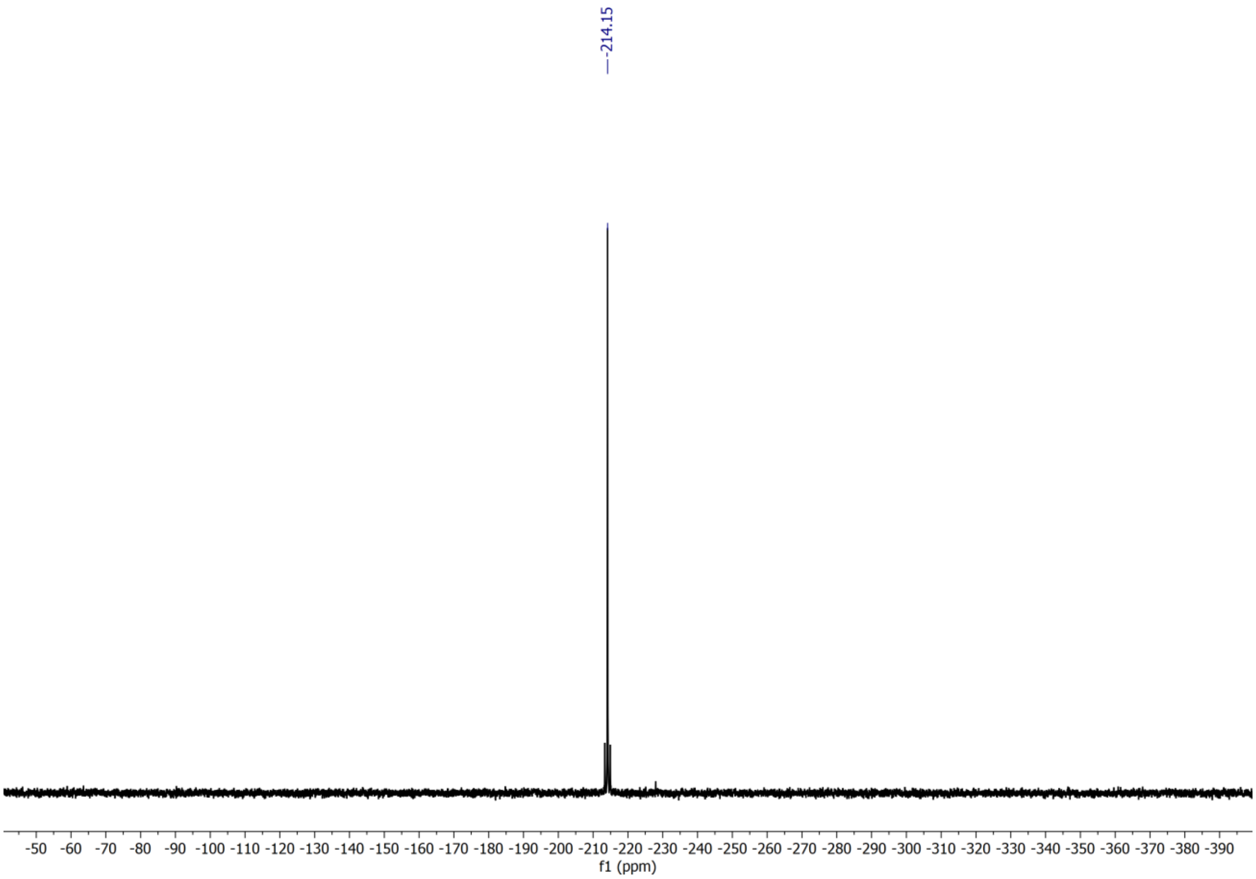

Figure S24. $^{119}Sn\{^{1}H\}$ NMR spectrum (223.77 MHz, 22 °C, $CDCl_3$) of $(L_2SnO)_3$ (**8**).

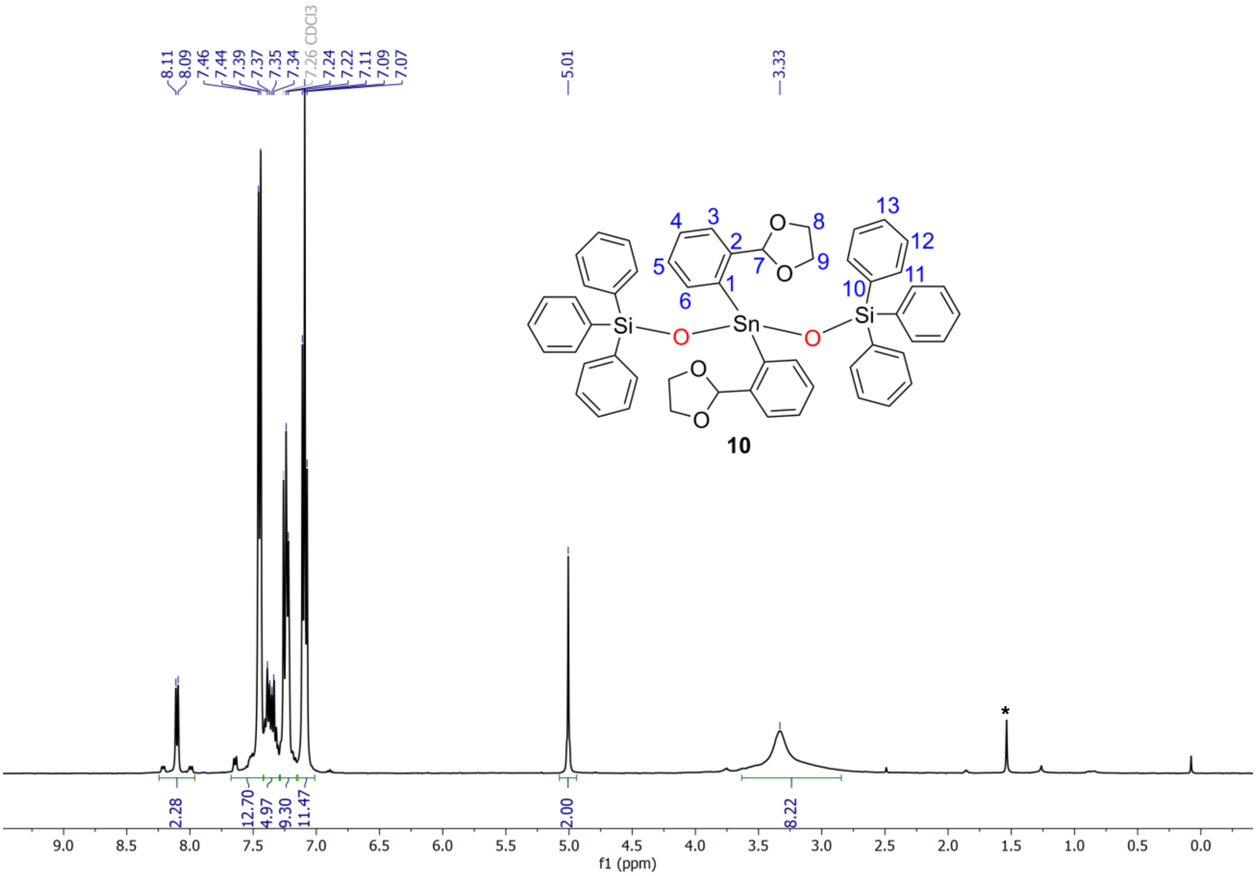

Figure S25. ^{1}H NMR spectrum (600.13 MHz, 21 °C, $CDCl_3$) of $L_2Sn(OSiPh_3)_2$ (**10**). Water traces are indicated by *.

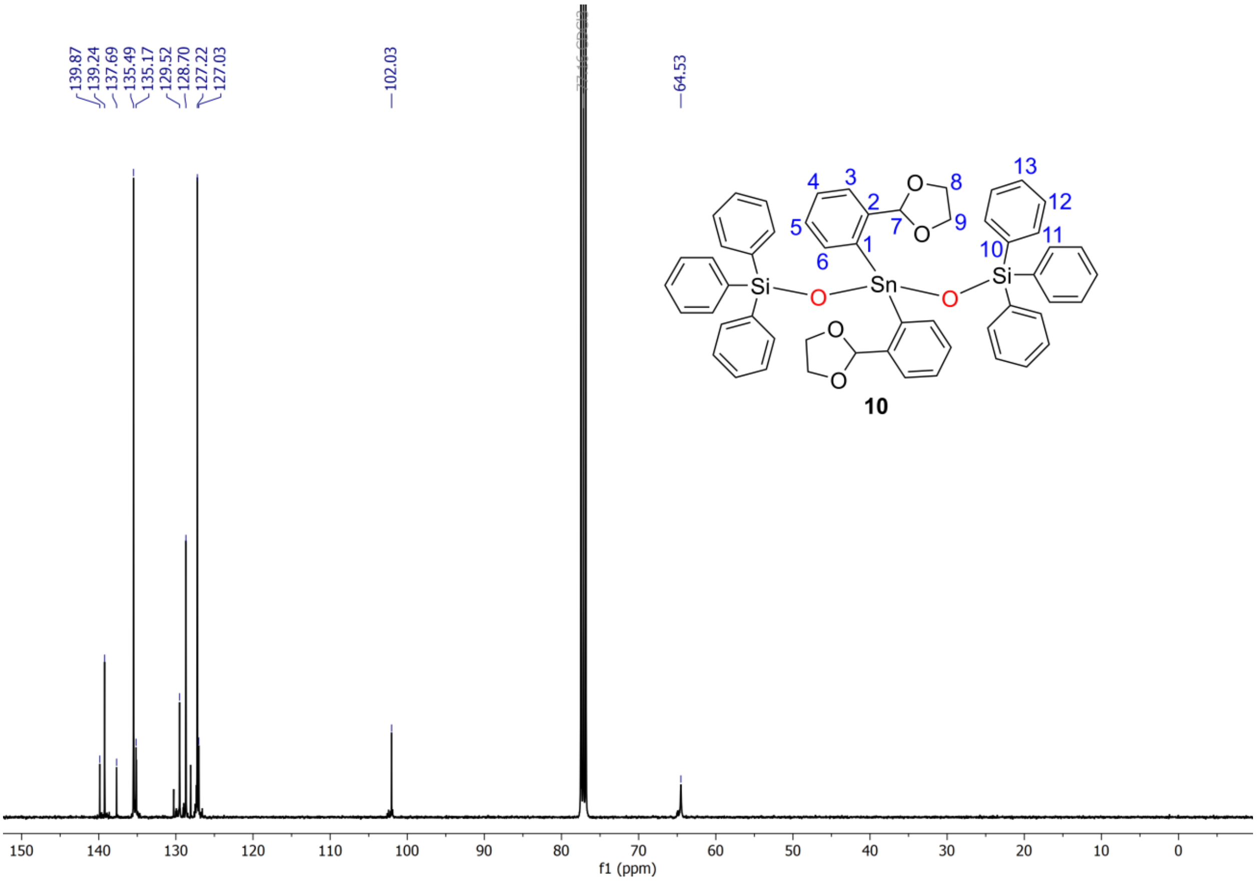

Figure S26. $^{13}C\{^{1}H\}$ NMR spectrum (150.92 MHz, 21 °C, $CDCl_3$) of $L_2Sn(OSiPh_3)_2$ (**10**).

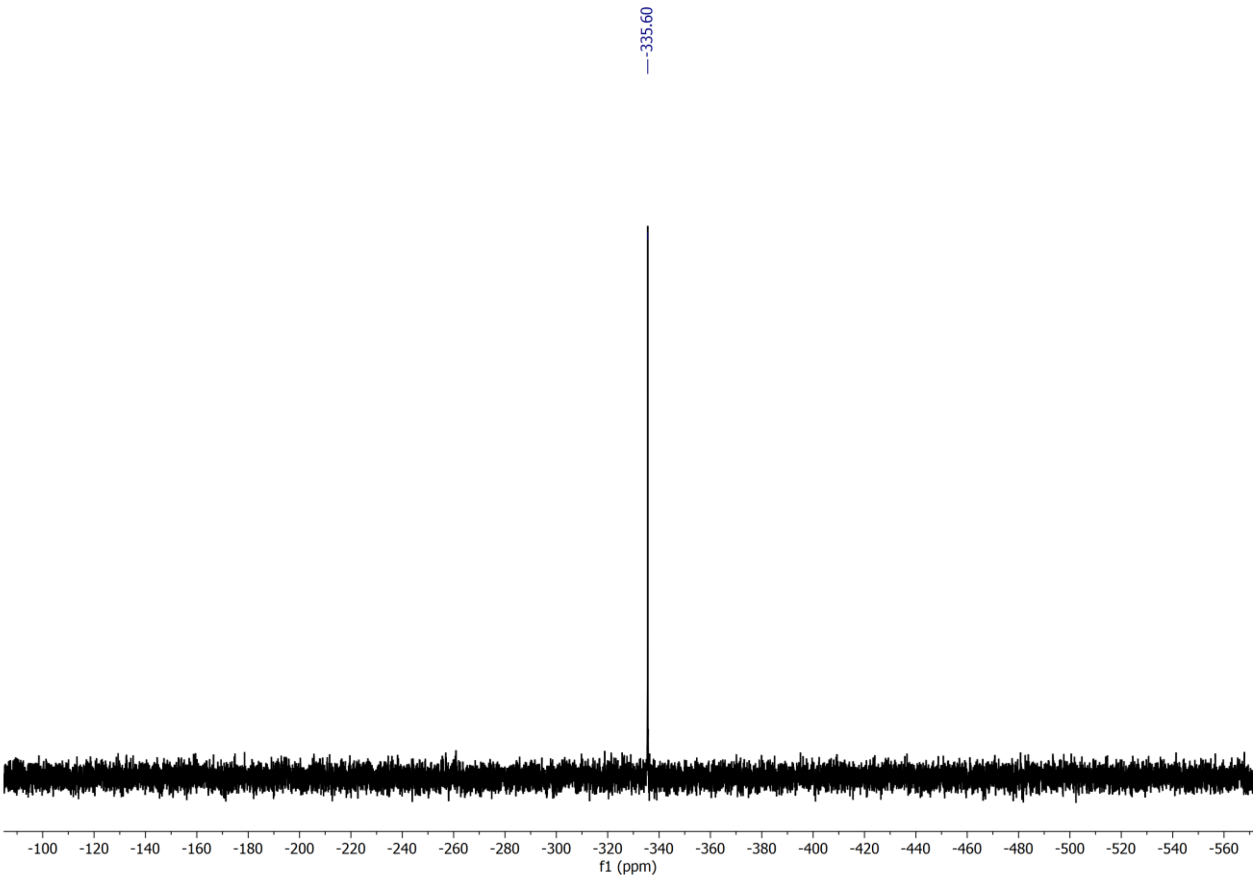

Figure S27. $^{119}Sn\{^1H\}$ NMR spectrum (223.73 MHz, 21 °C, $CDCl_3$) of $L_2Sn(OSiPh_3)_2$ (**10**).

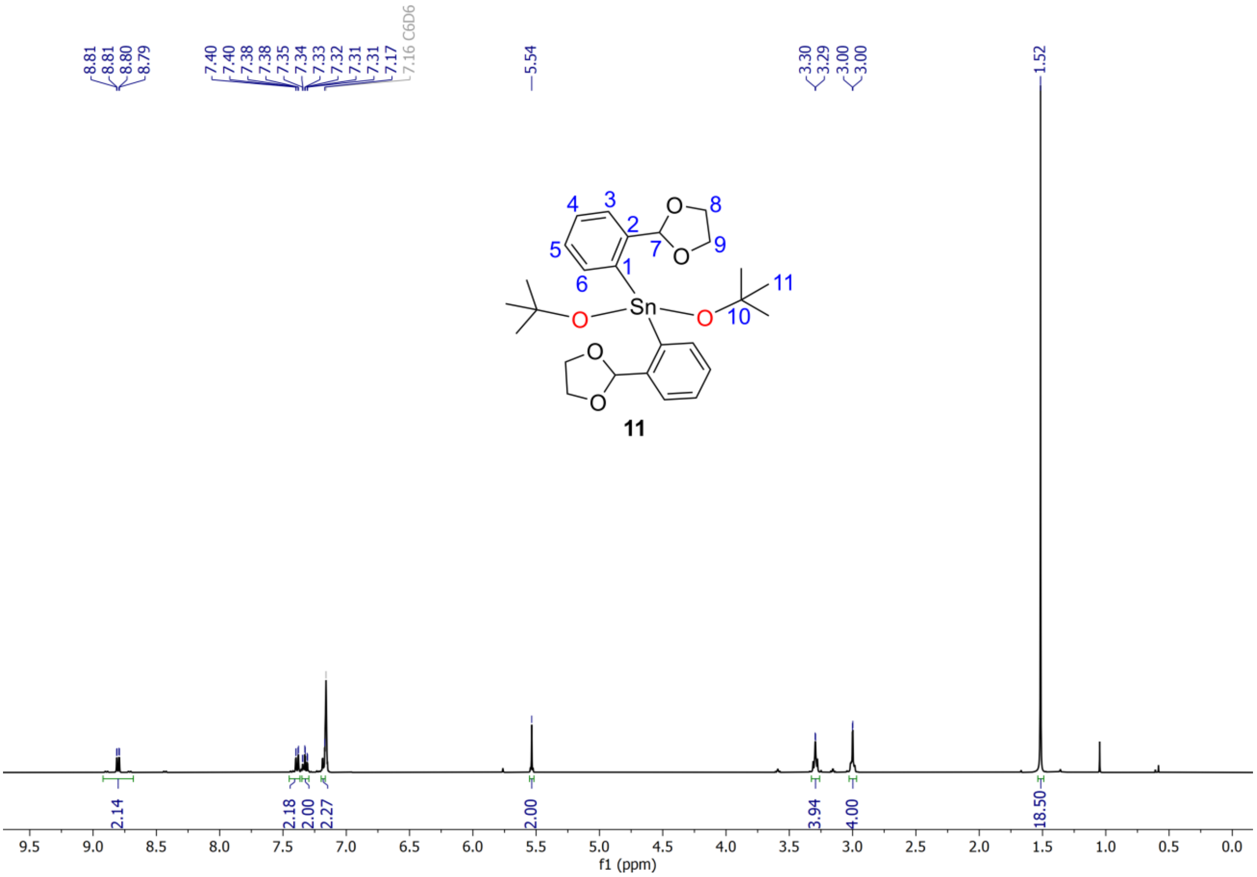

Figure S28. ^{1}H NMR spectrum (400.13 MHz, 21 °C, C_6D_6) of $L_2Sn(O^tBu)_2$ (**11**).

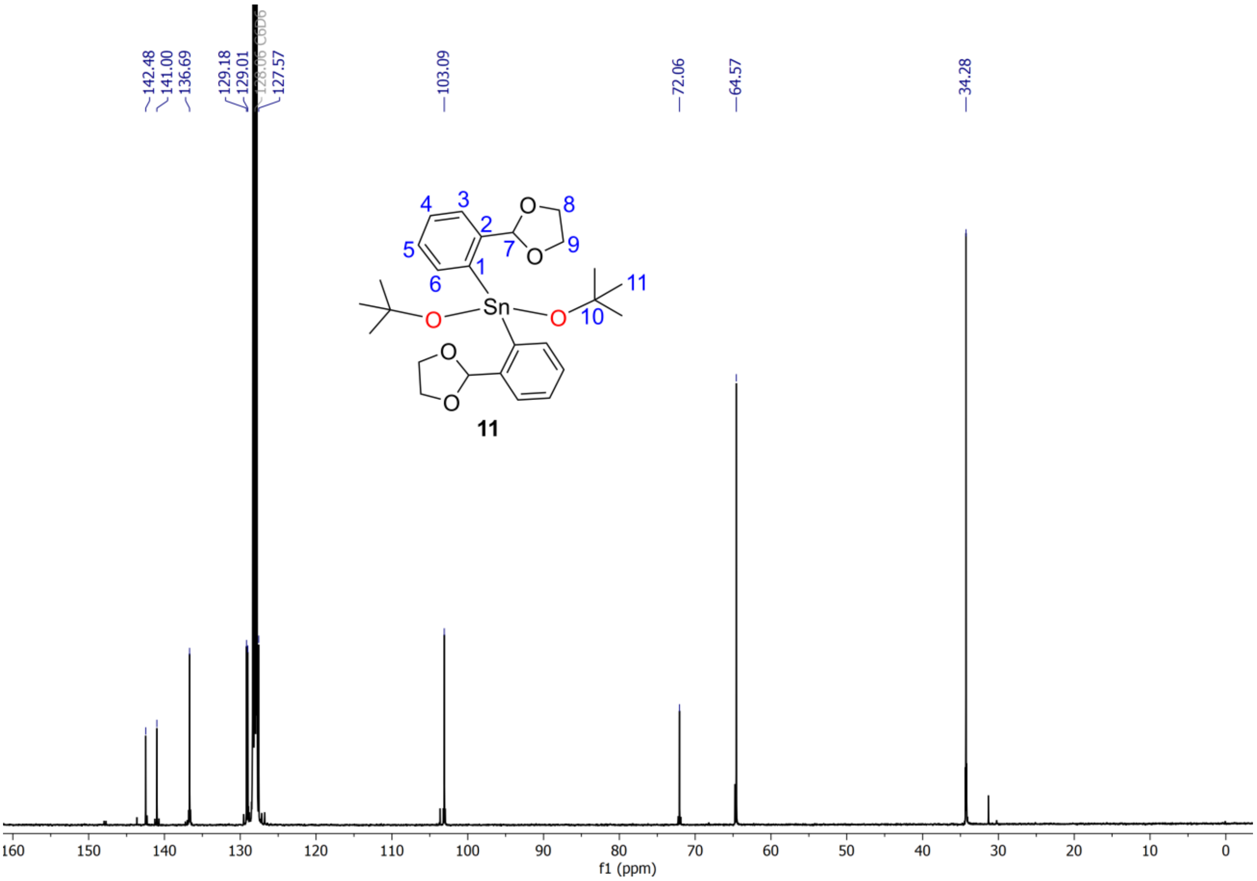

Figure S29. $^{13}C\{^{1}H\}$ NMR spectrum (100.62 MHz, 21 °C, C_6D_6) of $L_2Sn(O^tBu)_2$ (**11**).

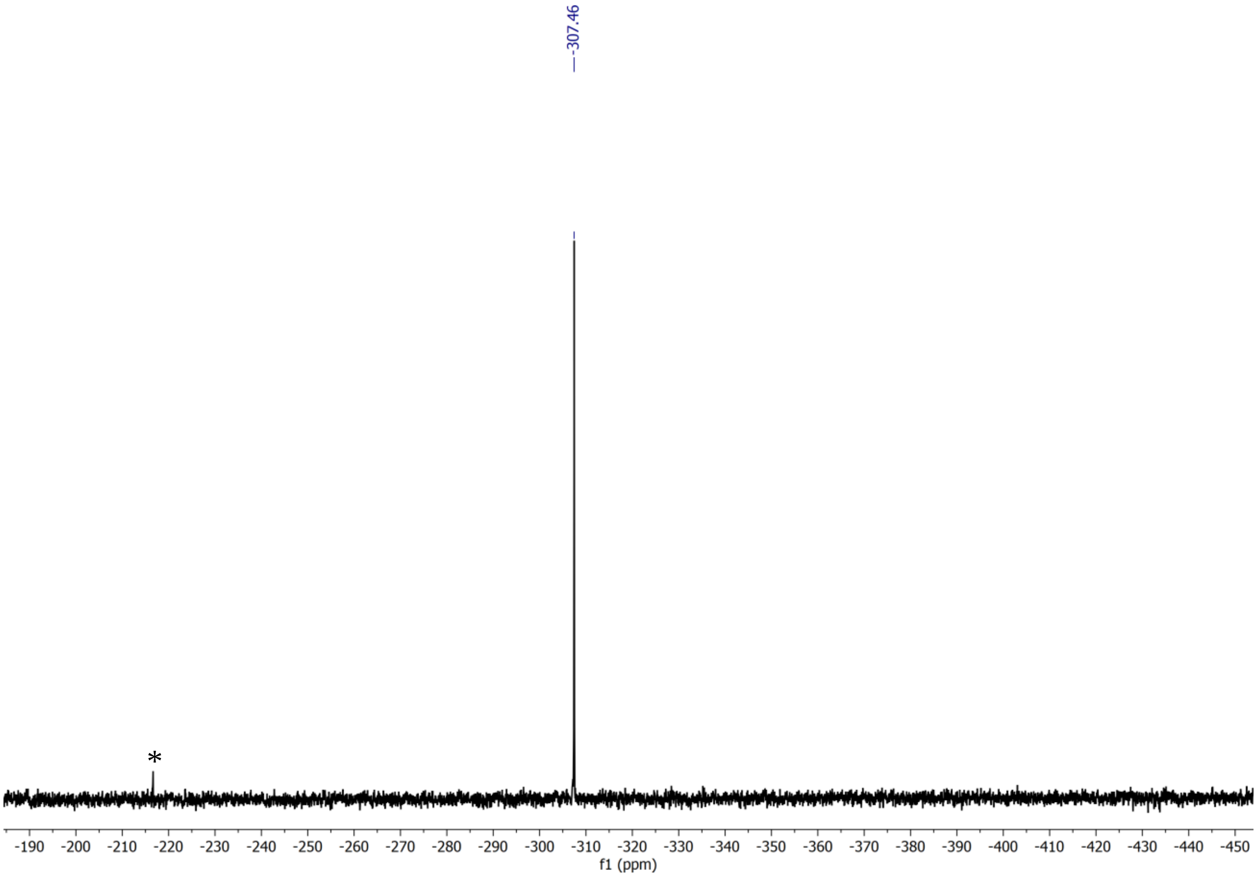

Figure S30. $^{119}Sn\{^1H\}$ NMR spectrum (149.19 MHz, 21 °C, C_6D_6) of $L_2Sn(O^tBu)_2$ (**11**). Resonance for decomposition product $(L_2SnO)_3$ (**8**) indicated by *.

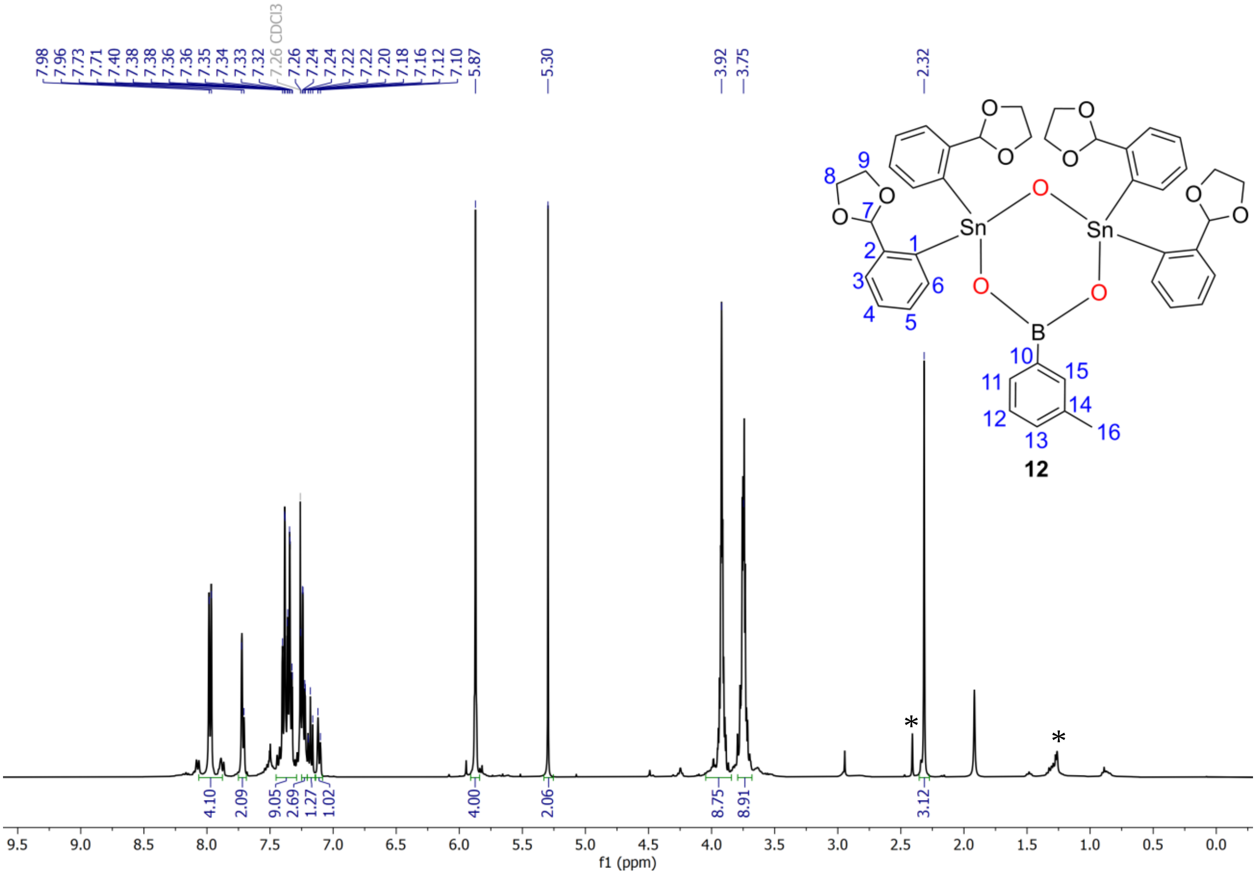

Figure S31. ^{1}H NMR spectrum (400.13 MHz, 21 °C, $CDCl_3$) of $(L_2SnO)_2OB$(*m*-tol) (**12**). Solvent traces (hexanes and toluene) indicated by *.

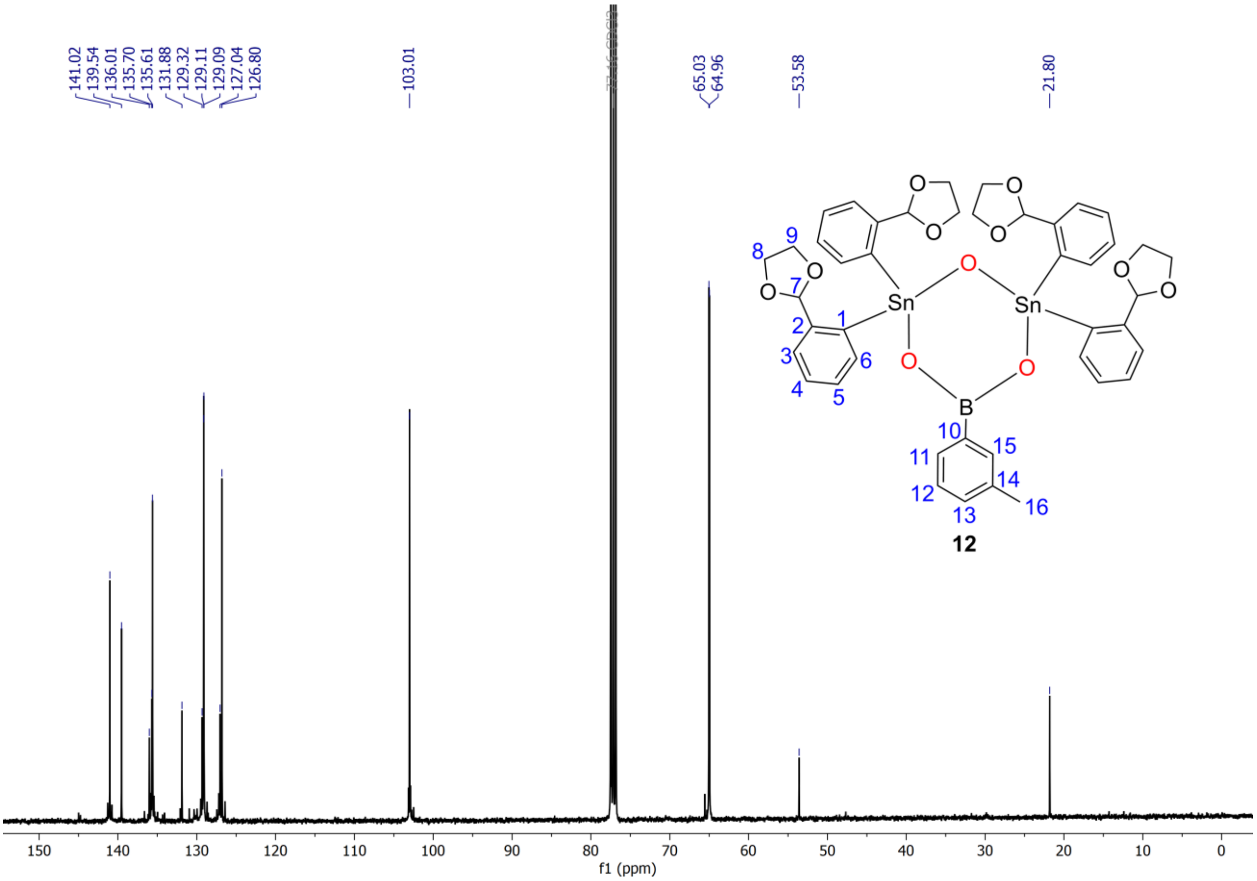

Figure S32. $^{13}C\{^{1}H\}$ NMR spectrum (100.62 MHz, 21 °C, $CDCl_3$) of $(L_2SnO)_2OB$(*m*-tol) (**12**).

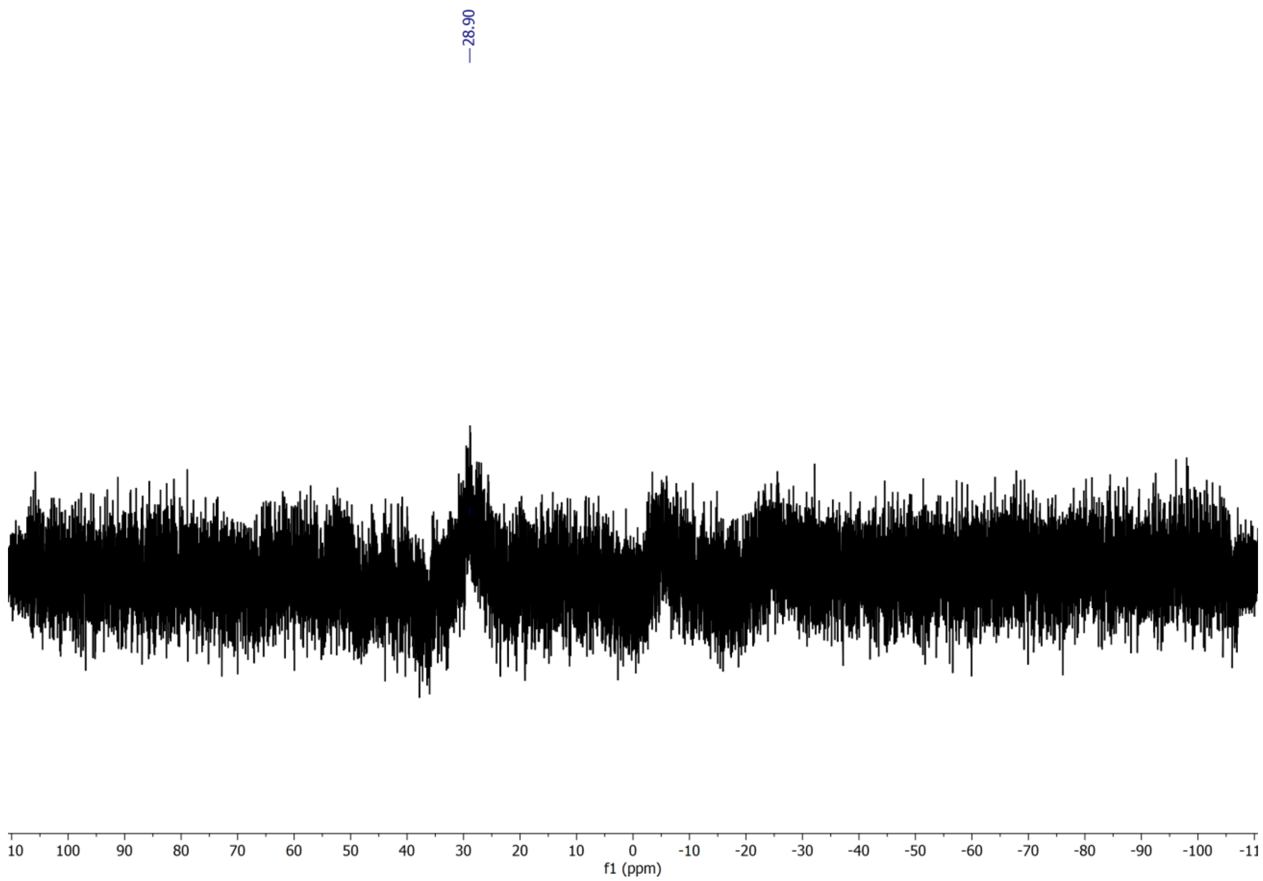

Figure S33. $^{11}B\{^1H\}$ NMR spectrum (128.38 MHz, 21 °C, $CDCl_3$) of $(L_2SnO)_2OB$(*m*-tol) (**12**).

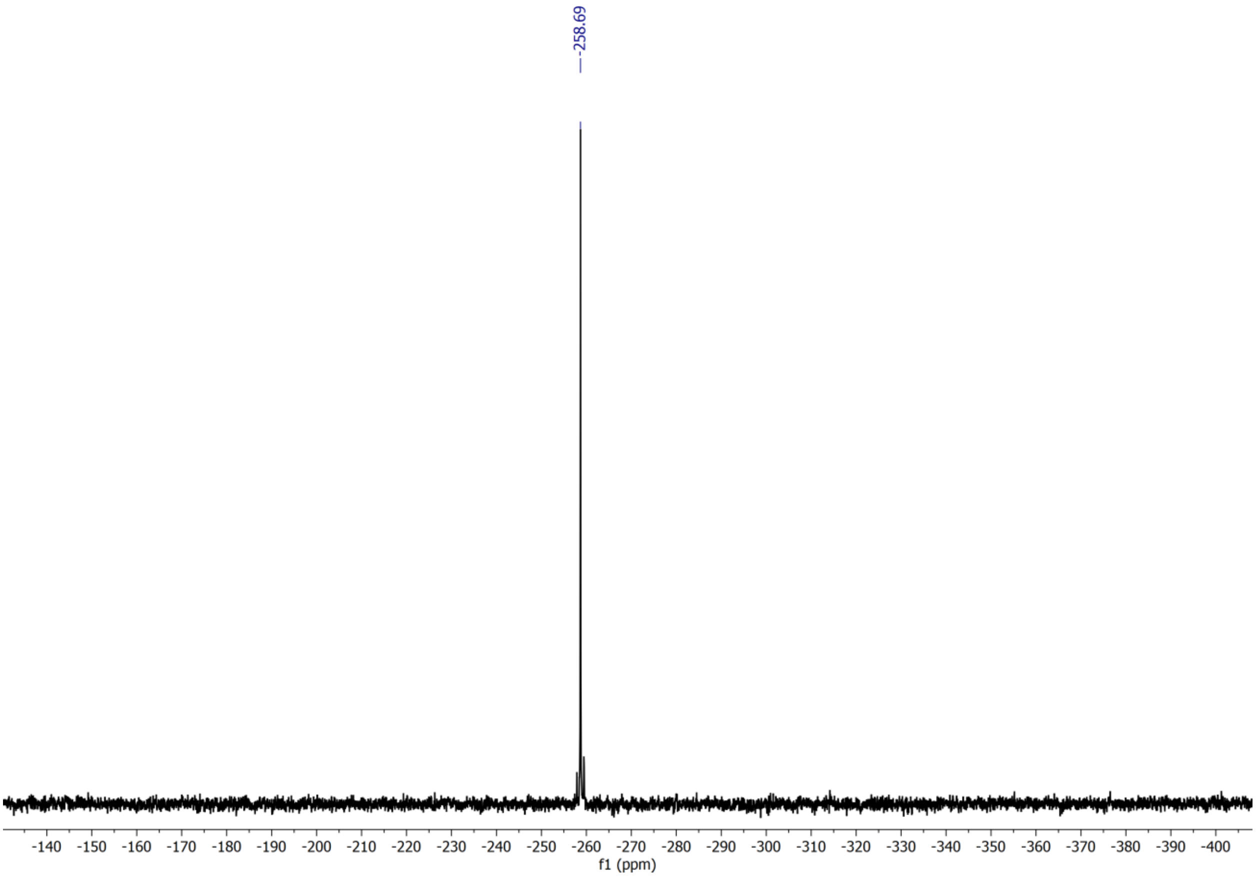

Figure S34. $^{119}Sn\{^1H\}$ NMR spectrum (149.19 MHz, 21 °C, $CDCl_3$) of $(L_2SnO)_2OB$(*m*-tol) (**12**).

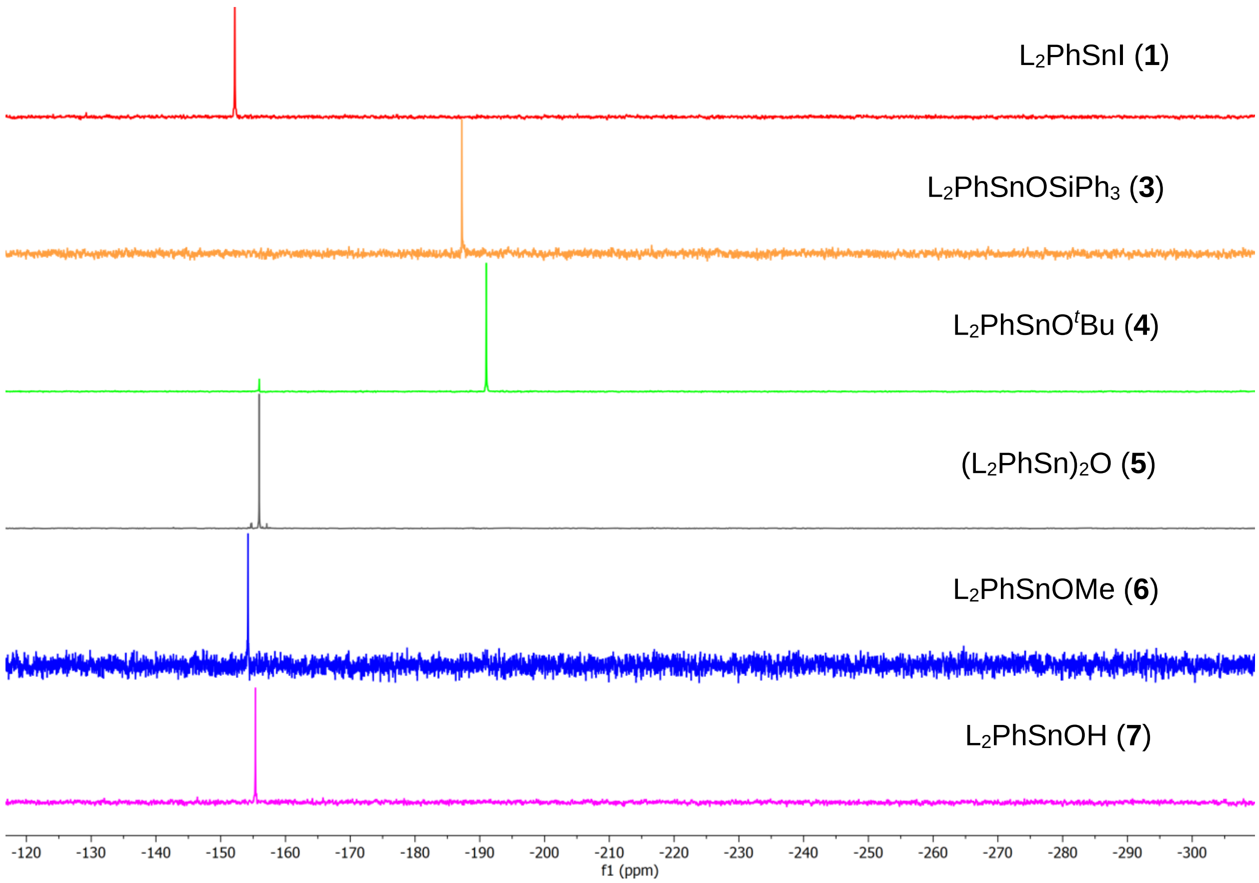

Figure S35. $^{119}Sn\{^1H\}$ NMR stacked spectra ($CDCl_3/C_6D_6$, 21 °C) for **1**, **3-7**.

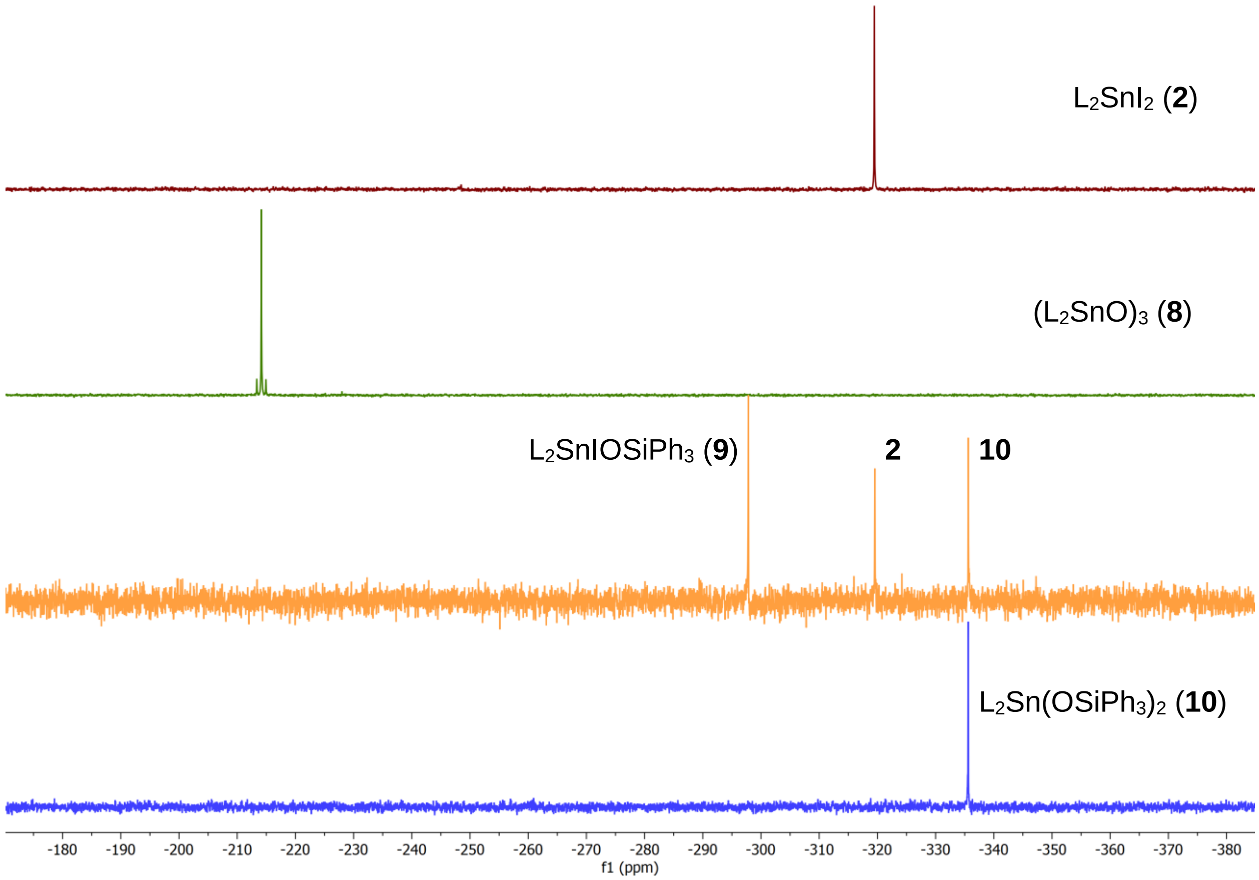

Figure S36. $^{119}Sn\{^1H\}$ NMR stacked spectra ($CDCl_3$, 21 °C) for **2**, **8-10**.

Table S1. X-ray crystallographic data and structure refinement for compounds **1-4**.

	L_2PhSnI (**1**)	L_2SnI_2 (**2**)	$L_2PhSnOSiPh_3$ (**3**)	L_2PhSnO^tBu (**4**)
Empirical formula	$C_{24}H_{23}IO_4Sn$	$C_{18}H_{18}I_2O_4Sn$	$C_{42}H_{38}O_5SiSn$	$C_{28}H_{32}O_5Sn$
Formula weight (g/mol)	621.01	670.81	769.50	567.22
Temperature (K)	100(2)	100(2)	100(2)	100(2)
Wavelength (Å)	0.71073	0.71073	0.71073	0.71073
Crystal system	monoclinic	monoclinic	orthorhombic	triclinic
Space group	*P*-21/c	*C*-2/c	$Pca2_1$	*P*-1
a (Å)	20.8084(7)	7.9394(3)	17.2580(7)	8.2252(4)
b (Å)	12.8972(4)	15.3647(5)	11.7705(5)	9.8585(5)
c (Å)	18.7105(6)	16.0408(5)	17.3289(7)	16.8123(9)
α (º)	90	90	90	83.950(2)
β (º)	116.6200(10)	96.8174(10)	90	83.496(2)
γ (º)	90	90	90	69.768(2)
Volume ($Å^3$)	4489.1(3)	1942.93(11)	3520.1(3)	1267.72(11)
Z	8	4	4	2
D_{calc} (g/cm^3)	1.838	2.293	1.452	1.486
Absorption coefficient (mm^{-1})	2.541	4.512	0.806	1.044
F(000)	2416	1256	1576	580
Crystal size (mm)	0.10x0.12x0.18	0.10x0.15x0.60	0.06x0.10x0.12	0.02x0.04x0.05
θ range for data collection (º)	1.92 to 28.78	2.65 to 28.39	2.09 to 28.30	2.21 to 28.32
Reflections collected	174397	34566	56862	92949
Independent reflections	11681	2444	8673	6294
	[R_{int}= 0.0555]	[R_{int} =0.0295]	[R_{int} =0.0488]	[R_{int} =0.0702]
Absorption correction	Multi-scan[71]	Multi-scan[71]	Multi-scan[71]	Multi-scan[71]
Data / restraints / parameters	11681/0/551	2444/0/114	8673/1/442	6294/0/310
Goodness-of-fit on F^2	1.129	1.192	1.049	1.079
Final *R* indices [I>2σ(I)]	R_1= 0.0326	R_1= 0.0136	R_1= 0.0246	R_1= 0.0229
	wR_2=0.0653	wR_2=0.0314	wR_2=0.0450	wR_2=0.0436
R indices (all data)	R_1= 0.0416	R_1= 0.0142	R_1= 0.0320	R_1= 0.0283
	wR_2= 0.0688	wR_2= 0.0316	wR_2= 0.0482	wR_2= 0.0460
Largest difference peak and hole (e $Å^{-3}$)	2.374 and –1.615	0.458 and –0.577	0.319 and –0.345	0.484 and –0.361
CCDC No.	2387283	2387279	2387280	2387282

Table S2. X-ray crystallographic data and structure refinement for compounds **5, 10-12**.

	$(L_2PhSn)_2O$ (**5**)	$L_2Sn(OSiPh_3)_2$ (**10**)	$L_2Sn(O^tBu)_2$ (**11**)	$(L_2SnO)_2OB$(*m*-tol) (**12**)
Empirical formula	$C_{48}H_{46}O_9Sn$	$C_{108}H_{94}O_{12}Si_4Sn_2$	$C_{26}H_{36}O_6Sn$	$C_{43}H_{43}BO_{11}Sn_2$
Formula weight (g/mol)	1004.23	1933.57	563.24	983.96
Temperature (K)	100(2)	100(2)	100(2)	100(2)
Wavelength (Å)	0.71073	0.71073	0.71073	0.71073
Crystal system	monoclinic	triclinic	orthorhombic	triclinic
Space group	*C*-2/c	*P*-1	*Pccn*	*P*-1
a (Å)	39.631(3)	10.6955(10)	17.2804(4)	9.4629(6)
b (Å)	10.4687(8)	19.1146(16)	8.6408(2)	11.9043(8)
c (Å)	23.4099(17)	23.0366(19)	17.1191(4)	19.9781(14)
α (º)	90	99.072(4)	90	88.909(2)
β (º)	115.374(2)	90.177(3)	90	81.862(2)
γ (º)	90	97.327(6)	90	76.351(2)
Volume ($Å^3$)	8775.5(11)	4611.4	2556.16(10)	2164.7
Z	8	2	4	2
D_{calc} (g/cm^3)	1.520	1.393	1.464	1.510
Absorption coefficient (mm^{-1})	1.194	0.657	1.037	1.211
F(000)	4048	1988	1160	988
Crystal size (mm)	0.03x0.08x0.10	0.05x0.10x0.11	0.09x0.13x0.15	0.10x0.12x0.15
θ range for data collection (º)	2.03 to 28.30	2.10 to 28.39	2.36 to 28.30	2.03 to 25.25
Reflections collected	197463	291701	107481	120636
Independent reflections	10891 [R_{int}= 0.0492]	25681 [R_{int}= 0.0463]	3190 [R_{int}= 0.0271]	7835 [R_{int}= 0.0221]
Absorption correction	Multi-scan[71]	Multi-scan[71]	Multi-scan[71]	Multi-scan[71]
Data / restraints / parameters	10891/0/532	25681/0/1136	3190/0/153	7835/0/515
Goodness-of-fit on F^2	1.049	1.059	1.096	1.068
Final *R* indices [$I>2\sigma(I)$]	R_1= 0.0286 wR_2=0.06781	R_1= 0.0438 wR_2=0.0971	R_1= 0.0220 wR_2=0.0540	R_1= 0.0264 wR_2=0.0593
R indices (all data)	R_1= 0.0343 wR_2= 0.0714	R_1= 0.0581 wR_2= 0.01067	R_1= 0.0242 wR_2= 0.0554	R_1= 0.0269 wR_2= 0.0595
Largest difference peak and hole (e $Å^{-3}$)	1.691 and –0.909	1.622 and –0.663	1.199 to –0.401	2.017 and –1.345
CCDC No.	2403846	2387281	2403845	2389609

[2-{$(CH_2O)_2CH$}C_6H_4]$_2$PhSnI (1)

- the crystal of **1** contains two distinct molecules in the asymmetric unit.

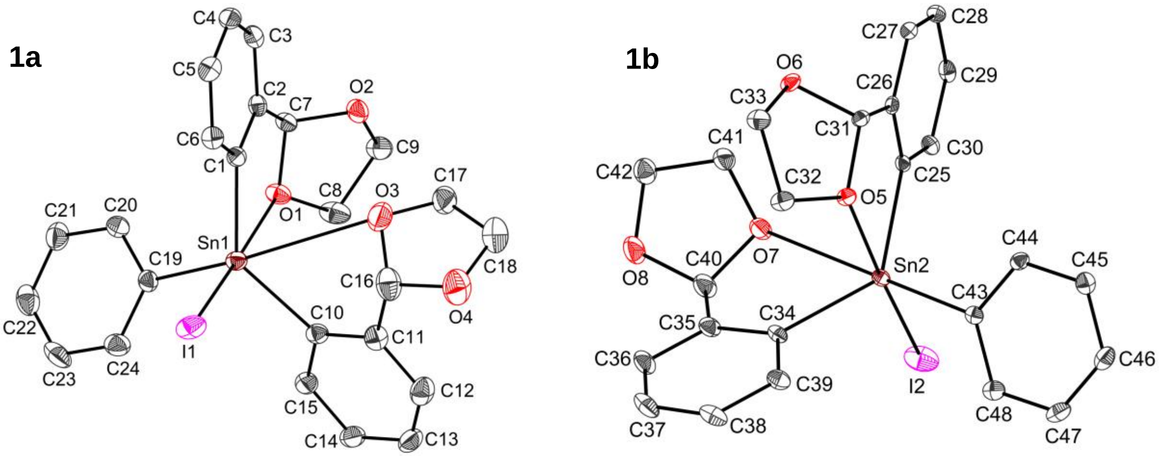

Figure S37. ORTEP representations of the two distinct molecules in the crystal of L_2PhSnI (**1**). Ellipsoids at the 50% probability level. H atoms not depicted for clarity.

Table S3. Interatomic distances (Å) and bond angles (°) in the distinct molecules of **1**.

	1a		1b
Sn1–C1	2.134(4)	Sn2–C25	2.144(3)
Sn1–C10	2.144(3)	Sn2–C34	2.138(3)
Sn1–C19	2.147(3)	Sn2–C43	2.156(3)
Sn1–I1	2.817(3)	Sn2–I2	2.798(3)
Sn1–O1	2.430(2)	Sn2–O5	2.492(2)
Sn1–O3	3.120(3)	Sn2–O7	2.847(2)
O1–Sn1–C1	74.22(2)	O5–Sn2–C25	72.76(1)
O1–Sn1–C19	85.75(1)	O5–Sn2–C43	85.65(1)
O1–Sn1–I1	174.35(5)	O5–Sn2–I2	175.72(5)
O1–Sn1–C10	89.90(1)	O5–Sn2–C34	83.57(1)
O1–Sn1–O3	79.49(8)	O5–Sn2–O7	95.68(8)
C1–Sn1–C19	110.54(1)	C25–Sn2–C43	107.51(1)
C1–Sn1–I1	100.44(8)	C25–Sn2–I2	102.98(8)
C1–Sn1–C10	132.37(1)	C25–Sn2–C34	130.90(1)
C1–Sn1–O3	68.27(1)	C25–Sn2–O7	72.05(1)
C19–Sn1–I1	97.99(9)	C43–Sn2–I2	96.22(8)
C19–Sn1–C10	112.71(1)	C43–Sn2–C34	112.93(1)
C19–Sn1–O3	164.99(1)	C43–Sn2–O7	178.37(1)
I1–Sn1–C10	92.50(9)	I2–Sn2–I2	99.19(9)
I1–Sn1–O3	96.91(5)	I2–Sn2–O7	82.39(5)
C10–Sn1–O3	64.75(1)	C34–Sn2–O7	68.21(1)

$[2\text{-}\{(CH_2O)_2CH\}C_6H_4]_2PhSnI$ (1)

- the crystal of **1** contains a racemate of S_{C7}-S_{C16}-**1a**, R_{C7}-R_{C16}-**1a**, S_{C7}-S_{C16}-**1b** and R_{C7}-R_{C16}-**1b** isomers

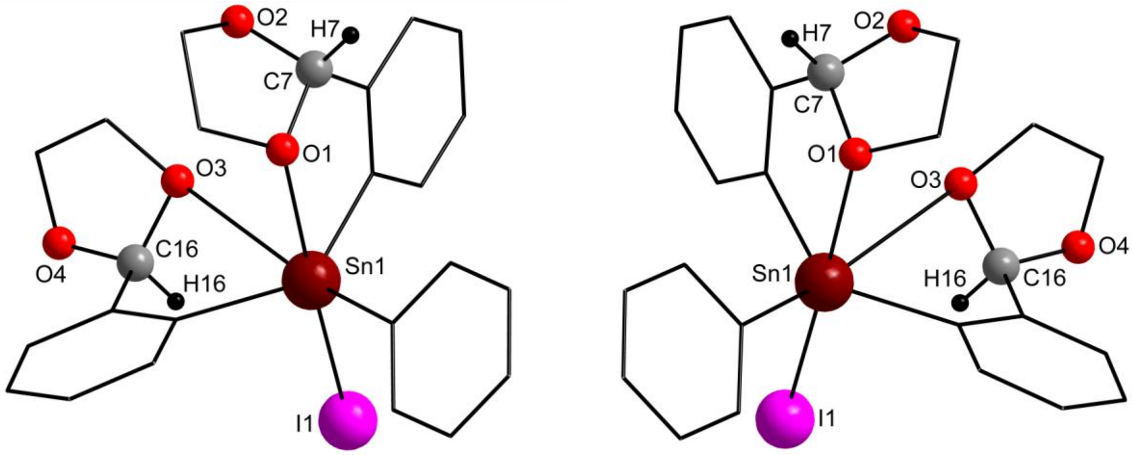

Figure S38. Molecular solid-state structure of S_{C7}-S_{C16}-**1a** isomer (*left*) and R_{C7}-R_{C16}-**1a** isomer (*right*) in the crystal of L_2PhSnI (**1**). H atoms not depicted for clarity, except H7 and H16.

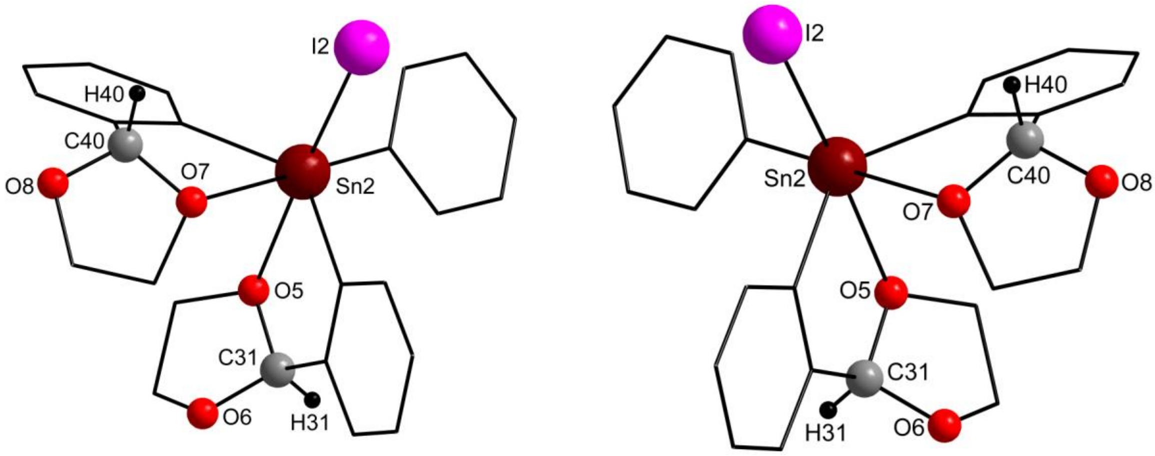

Figure S39. Molecular solid-state structure of S_{C31}-S_{C40}-**1b** isomer (*left*) and R_{C7}-R_{C16}-**1b** isomer (*right*) in the crystal of L_2PhSnI (**1**). H atoms not depicted for clarity, except H7 and H16.

$[2\text{-}\{(CH_2O)_2CH\}C_6H_4]_2SnI_2$ (2)

- the crystal of **2** contains a 1:1 mixture of S_{C7} -**2** and R_{C7}-**2** isomers

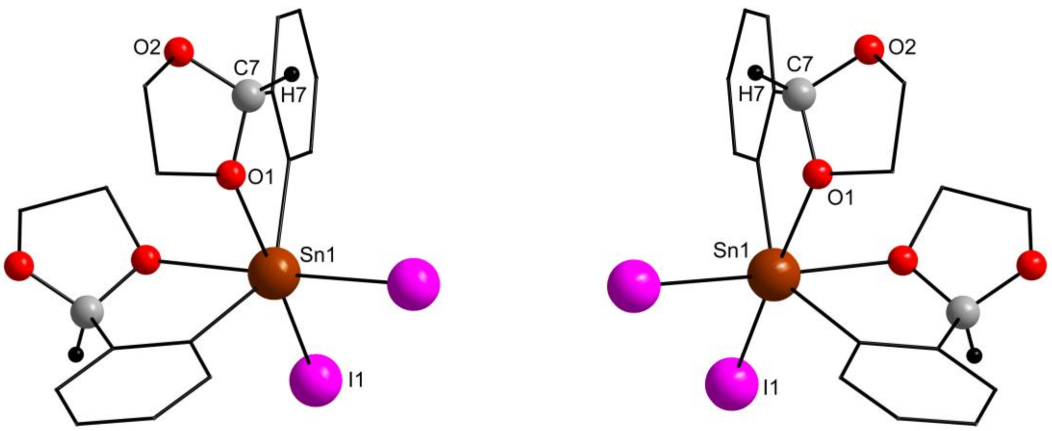

Figure S40. Molecular solid-state structure of S_{C7}-**2** isomer (*left*) and R_{C7}-**2** isomer (*right*) in the crystal of L_2SnI_2 (**2**). H atoms not depicted for clarity, except H7.

[2-{$(CH_2O)_2$CH}C_6H_4]$_2$PhSnOSiPh$_3$ (3)

- the crystal of **3** contains a 1:1 mixture of S_{C7}-R_{C16}-**3** and R_{C7}-S_{C16}-**3** isomers

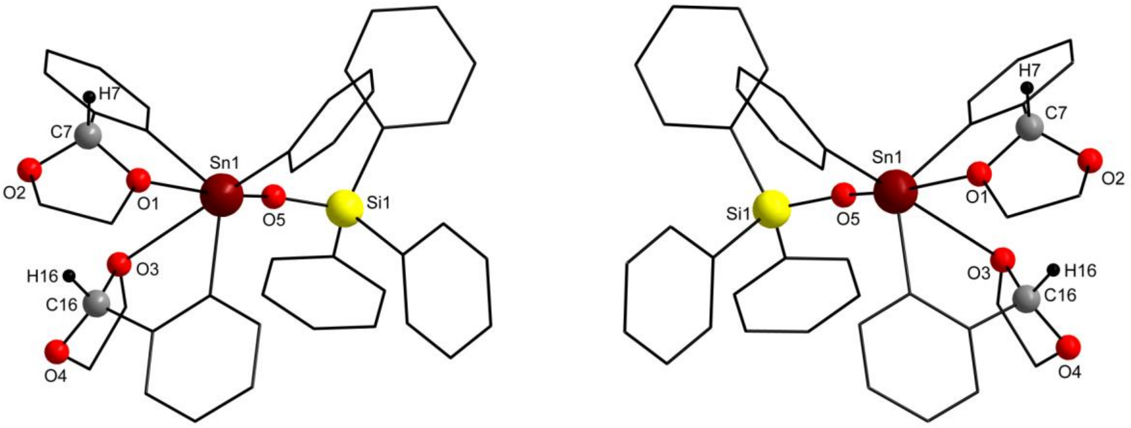

Figure S41. Molecular solid-state structure of S_{C7}-R_{C16}-**3** isomer (left) and R_{C7}-S_{C16}-**3** isomer (right) in the crystal of L_2PhSnOSiPh$_3$ (**3**). H atoms not depicted for clarity, except H7 and H16.

[2-{$(CH_2O)_2$CH}C_6H_4]$_2$PhSnO^{t}Bu (4)

- the crystal of **4** contains a 1:1 mixture of S_{C7}-S_{C16}-**4** and R_{C7}-R_{C16}-**4** isomers

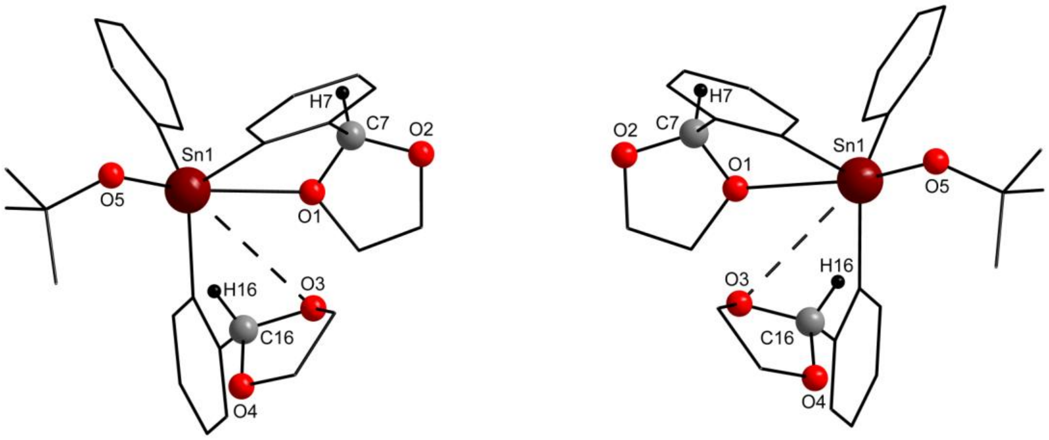

Figure S42. Molecular solid-state structure of S_{C7}-S_{C16}-**4** isomer (left) and R_{C7}-R_{C16}-**4** isomer (right) in the crystal of L_2PhSnO^{t}Bu (**4**). H atoms not depicted for clarity, except H7 and H16.

{[2-{(CH$_2$O)$_2$CH}C$_6$H$_4$]$_2$PhSn}$_2$O (5)

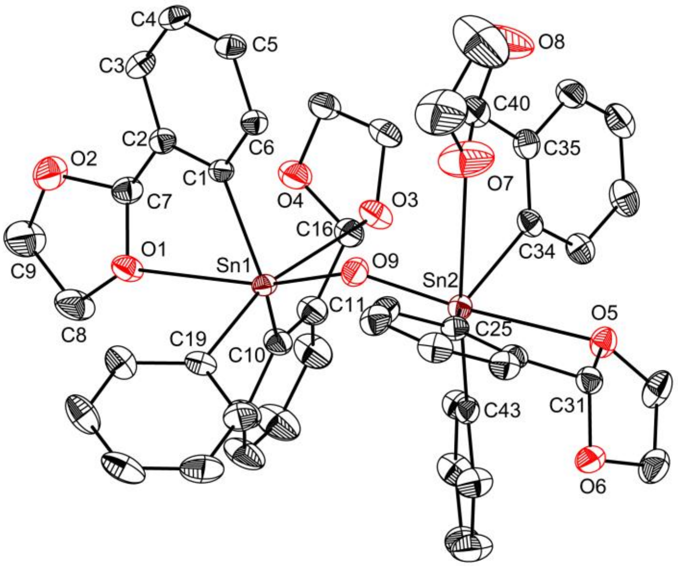

Figure S43. ORTEP Representation of the molecular solid-state structure of (L$_2$PhSnO)$_2$ (**5**). Only the R_{C7}-S_{C16}-S_{C31}-R_{C40}-**5** isomer is depicted.

Table S4. Interatomic distances (Å) and bond angles (°) in the distinct molecules of **5**.

	**5**		**5**
Sn1–C1	2.140(2)	Sn2–C25	2.143(2)
Sn1–C10	2.150(2)	Sn2–C34	2.151(2)
Sn1–C19	2.155(2)	Sn2–C43	2.153(2)
Sn1–O9	1.9668(17)	Sn2–O9	1.9631(17)
Sn1–O1	2.755(2)	Sn2–O5	2.795(2)
Sn1–O3	2.890(2)	Sn2–O7	2.924(3)
O1–Sn1–O9	165.47(7)	O5–Sn2–O9	164.65(6)
O3–Sn1–C19	164.91(7)	O7–Sn2–C43	172.20(10)
C1–Sn1–C10	121.37(9)	C25–Sn2–C34	125.93(8)
C1–Sn1–O3	82.85(7)	C25–Sn2–O7	72.32(9)
C1–Sn1–C19	112.10(9)	C25–Sn2–C43	113.58(9)
C10–Sn1–O3	65.90(9)	C34–Sn2–07	65.23(9)
C10–Sn1–C19	105.70(9)	C34–Sn2–C43	107.00(9)
O1–Sn1–C1	67.77(7)	O5–Sn2–C34	79.39(7)
O1–Sn1–C10	73.72(9)	O5–Sn2–C25	67.58(7)
O1–Sn1–C19	84.01(8)	O5–Sn2–C43	88.88(7)
O1–Sn1–O3	104.49(6)	O5–Sn2–O7	88.86(7)
O9–Sn1–C1	98.12(8)	O9–Sn2–C25	98.97(8)
O9–Sn1–C10	118.57(9)	O9–Sn2–C34	104.54(9)
O9–Sn1–O3	76.04(6)	O9–Sn2–O7	79.67(9)
O9–Sn1–C19	98.91(9)	O9–Sn2–C43	103.79(8)
Sn1–O9–Sn2	143.26(10)		

- the crystal of **5** contains a racemate of R_{C7}-S_{C16}-S_{C31}-R_{C40}-**5**and S_{C7}-R_{C16}-R_{C31}-S_{C40}-**5** isomers

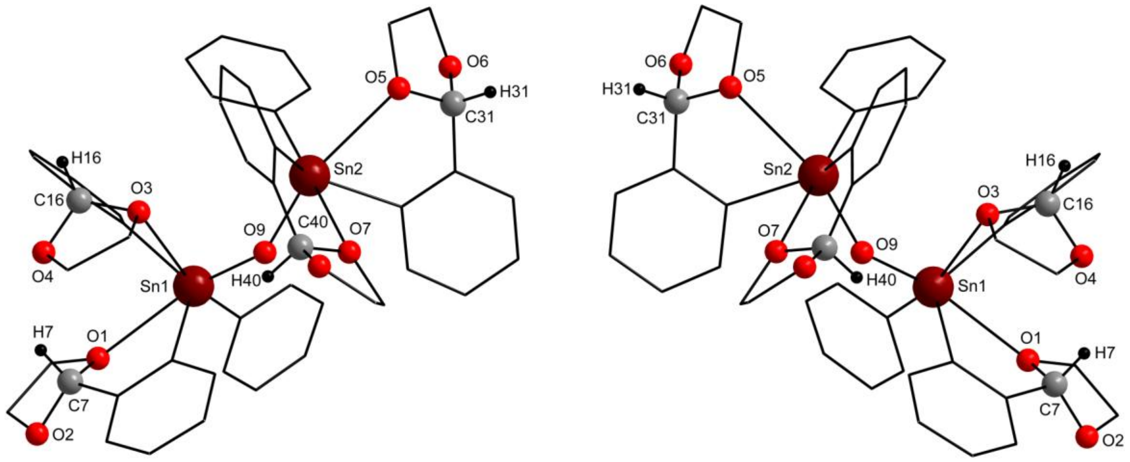

Figure S44. Molecular solid-state structure of R_{C7}-S_{C16}-S_{C31}-R_{C40}-**5** isomer (*left*) and S_{C7}-R_{C16}-R_{C31}-S_{C40}-**5** isomer (*right*) in the crystal of $(L_2PhSn)_2O$ (**5**). H atoms not depicted for clarity, except H7, H16, H31 and H40.

$[2\text{-}\{(CH_2O)_2CH\}C_6H_4]_2Sn(OSiPh_3)_2$ (10)

- the crystal of **10** contains two distinct molecules in the asymmetric unit.

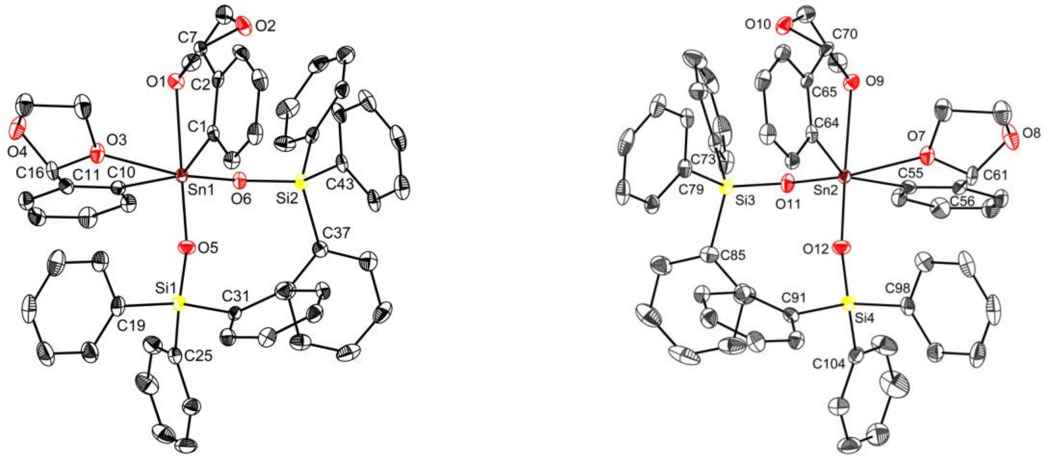

Figure S45. ORTEP representations of the two distinct molecules in the crystal of $L_2Sn(OSiPh_3)_2$ (**10**). Ellipsoids at the 50% probability level. H atoms not depicted for clarity.

Table S5. Interatomic distances (Å) and bond angles (°) in the distinct molecules of **10**.

	**10a**		**10b**
Sn1–C1	2.125(3)	Sn2–C55	2.123(3)
Sn1–C10	2.118(3)	Sn2–C64	2.124(3)
Sn1–O1	2.492(3)	Sn2–O7	2.538(3)
Sn1–O3	2.567(3)	Sn2–O9	2.498(3)
Sn1–O5	1.996(2)	Sn2–O11	1.994(3)
Sn1–O6	1.991(3)	Sn2–O12	1.988(3)
O1–Sn1–C1	73.21(1)	O7–Sn2–C55	72.16(1)
O1–Sn1–C10	87.30(1)	O7–Sn2–C64	80.79(1)
O1–Sn1–O3	81.50(1)	O7–Sn2–O9	81.74(1)
O1–Sn1–O5	171.97(1)	O7–Sn2–O11	166.67(1)
O1–Sn1–O6	87.11(1)	O7–Sn2–O12	91.60(1)
C1–Sn1–C10	148.09(1)	C55–Sn2–C64	147.60(1)
C1–Sn1–O3	80.38(1)	C55–Sn2–O9	85.62(1)
C1–Sn1–O5	99.25(1)	C55–Sn2–O11	98.18(1)
C1–Sn1–O6	104.79(1)	C55–Sn2–O12	99.39(1)
C10–Sn1–O3	71.77(1)	C64–Sn2–O9	72.92(1)
C10–Sn1–O5	98.25(1)	C64–Sn2–O11	105.06(1)
C10–Sn1–O6	99.04(1)	C64–Sn2–O12	98.85(1)
O3–Sn1–O5	94.98(1)	C9–Sn2–O11	88.49(1)
O3–Sn1–O6	165.60(1)	O9–Sn2–O12	170.09(1)
O5–Sn1–O6	97.67(1)	O11–Sn2–O12	99.18(1)
Sn1–O5–Si1	168.41(2)	Sn2–O11–Si3	150.02(1)
Sn1–O6–Si2	149.68(1)	Sn2–O12–Si4	170.98(1)

- the crystal of **10** contains a racemate of S_{C7}-R_{C16}-**10a**, R_{C7}-R_{C16}-**10a**, S_{C7}-S_{C16}-**10b** and R_{C7}-R_{C16}-**10b** isomers

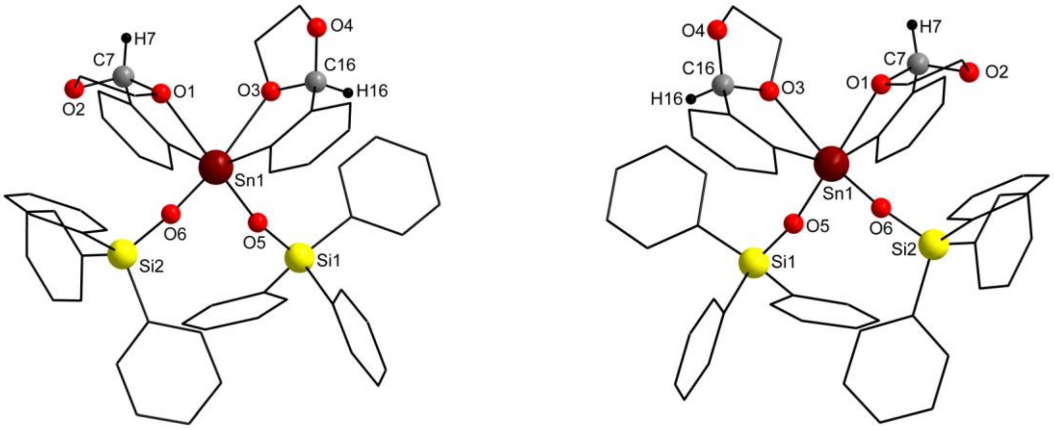

Figure S46. Molecular solid-state structure of S_{C7}-R_{C16}-**10a** isomer (*left*) and R_{C7}-S_{C16}-**10a** isomer (*right*) in the crystal of $L_2Sn(OSiPh_3)_2$ (**10**). H atoms not depicted for clarity, except H7 and H16.

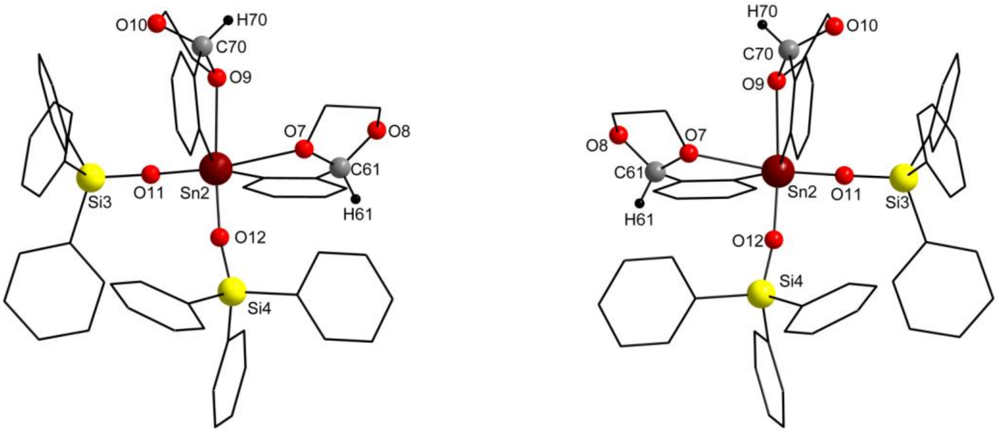

Figure S47. Molecular solid-state structure of S_{C61}-R_{C70}-**10b** isomer (*left*) and R_{C61}-S_{C70}-**10b** isomer (*right*) in the crystal of $L_2Sn(OSiPh_3)_2$ (**10**). H atoms not depicted for clarity, except H61 and H70.

[2-{$(CH_2O)_2CH$}C_6H_4]$_2$Sn(O^{t}Bu)$_2$ (11)

- the crystal of **11** contains a racemate of R_{C7}-$R_{C7'}$-**11** and S_{C7}-$S_{C7'}$-**11** isomers

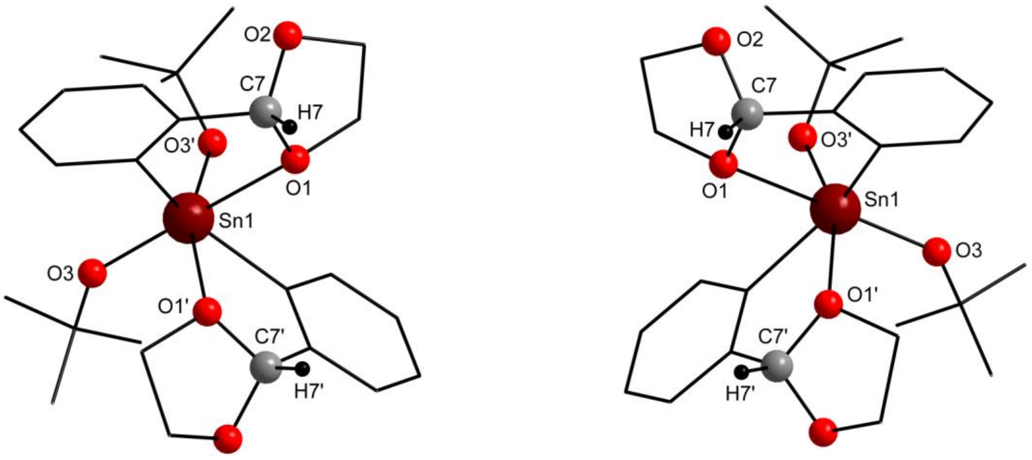

Figure S48. Molecular solid-state structure of R_{C7}-$R_{C7'}$-**11** isomer (left) and S_{C7}-$S_{C7'}$-**11** isomer (right) in the crystal of $L_2Sn(O^tBu)_2$ (**11**). H atoms not depicted for clarity, except H7.

Table S6. Interatomic distances (Å) and bond angles (°) in the distinct molecules of **11**.

	**11**
Sn1–C1	2.1362(16)
Sn1–O3	1.9908(13)
Sn1–O1	2.563(1)
O1–Sn1–O3	160.64(5)
C1–Sn1–C1’	138.92(9)
C1–Sn1–O1	71.32(5)
C1–Sn1–O3	94.25(6)
C1–Sn1–O3’	110.02(6)
C1–Sn1–O1’	76.11(5)
O3–Sn1–O1’	90.25(5)
O3–Sn1–O3’	107.17(8)
O1–Sn1–O1’	74.05(4)

{[2-{(CH$_2$O)$_2$CH}C$_6$H$_4$]$_2$SnO}OB-(3-C$_6$H$_4$-CH$_3$) (12)

- the crystal of **12** contains a 1:1 mixture of S_{C7}-S_{C16}-S_{C25}-R_{C34}-**12** and R_{C7}-R_{C16}-R_{C25}-S_{C34}-**12** isomers

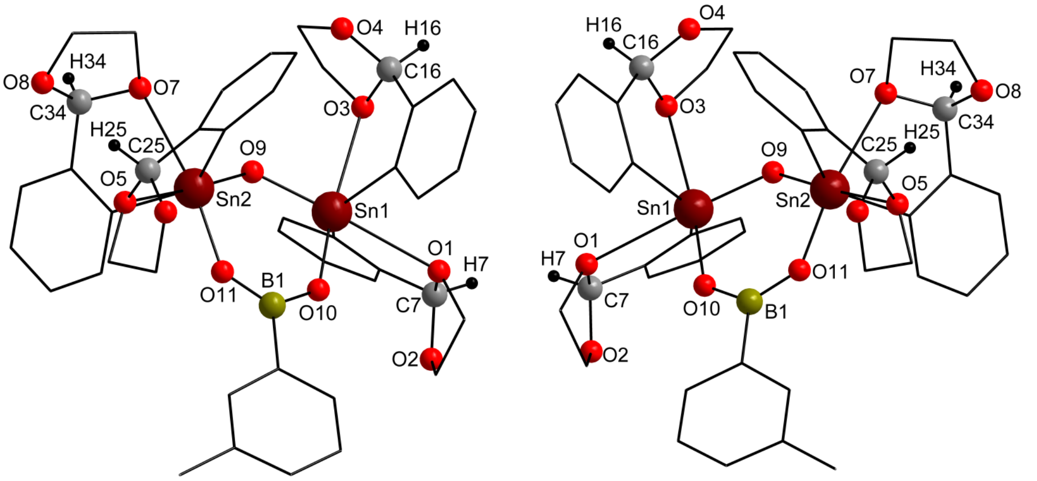

Figure S49. Molecular solid-state structure of S_{C7}-S_{C16}-S_{C25}-R_{C34}-**12** isomer (*left*) and R_{C7}-R_{C16}-R_{C25}-S_{C34}-**12** isomer (*right*) in the crystal of (L$_2$SnO)$_2$OB(*m*-tol) (**12**). H atoms not depicted for clarity, except H7, H16, H25 and H34.

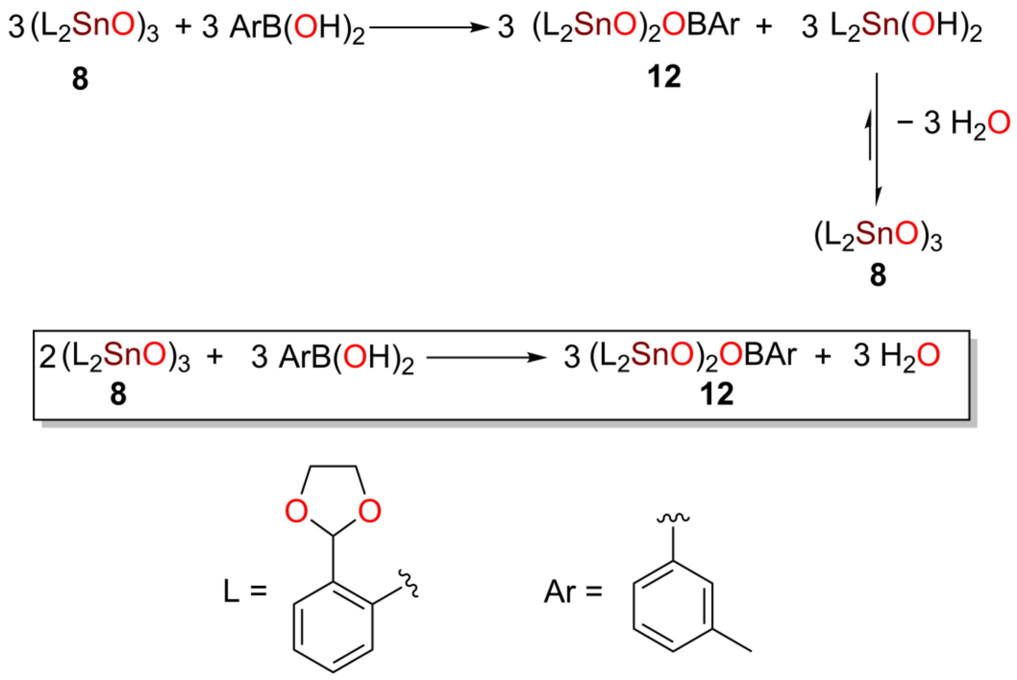

Figure S50. Reaction pathway for the formation of stannaboroxane **12.**

($(L_2SnO)_2OB$(*m*-tol) (12)

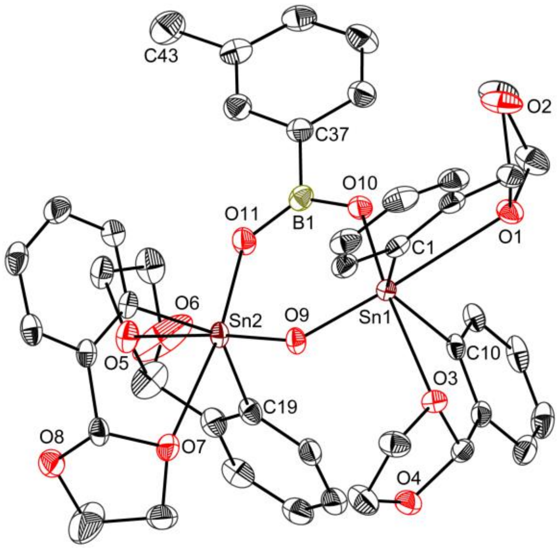

Figure S51. ORTEP representations of $(L_2SnO)_2OB$(*m*-tol) (**12**). Ellipsoids at the 50% probability level. H atoms not depicted for clarity.

Table S7. Interatomic distances (Å) and bond angles (°) in **12**.

	12		12
Sn1–C1	2.131(3)	Sn2–C19	2.132(3)
Sn1–C10	2.125(3)	Sn2–C28	2.121(3)
Sn1–O1	2.781(2)	Sn2–O5	2.604(2)
Sn1–O3	2.624(1)	Sn2–O7	2.635(2)
Sn1–O9	1.967(2)	Sn2–O9	1.982(2)
Sn1–O10	2.008(2)	Sn2–O11	2.006(2)
O1–Sn1–O9	169.73(7)	O5–Sn2–O9	173.56(7)
O3–Sn1–O10	171.75(7)	O7–Sn2–O11	170.47(7)
C1–Sn1–C10	132.97(10)	C28–Sn2–C19	139.11(10)
C1–Sn1–O3	80.40(8)	C28–Sn2–O7	71.23(8)
C1–Sn1–O10	104.91(9)	C28–Sn2–O11	99.32(10)
C10–Sn1–O3	70.53(7)	C19–Sn2–07	82.14(8)
C10–Sn1–O10	101.38(10)	C19–Sn2–O11	105.21(10)
O1–Sn1–C1	67.93(7)	O5–Sn2–C19	70.67(9)
O1–Sn1–C10	78.65(8)	O5–Sn2–C28	80.99(8)
O1–Sn1–O10	81.14(7)	O5–Sn2–O11	82.33(7)
O1–Sn1–O3	98.25(1)	O5–Sn2–O7	94.71(7)
O9–Sn1–C1	101.81(9)	O9–Sn2–C28	104.89(9)
O9–Sn1–C10	109.78(9)	O9–Sn2–C19	102.92(10)
O9–Sn1–O3	82.47(7)	O9–Sn2–O7	84.88(7)
O9–Sn1–O10	102.32(8)	O9–Sn2–O11	99.07(8)
Sn1–O9–Sn2	123.41(9)		
Sn1–O10–B1	129.07(18)	Sn2–O11–B1	134.09(2)

High-resolution mass spectrometry (HRMS spectra)

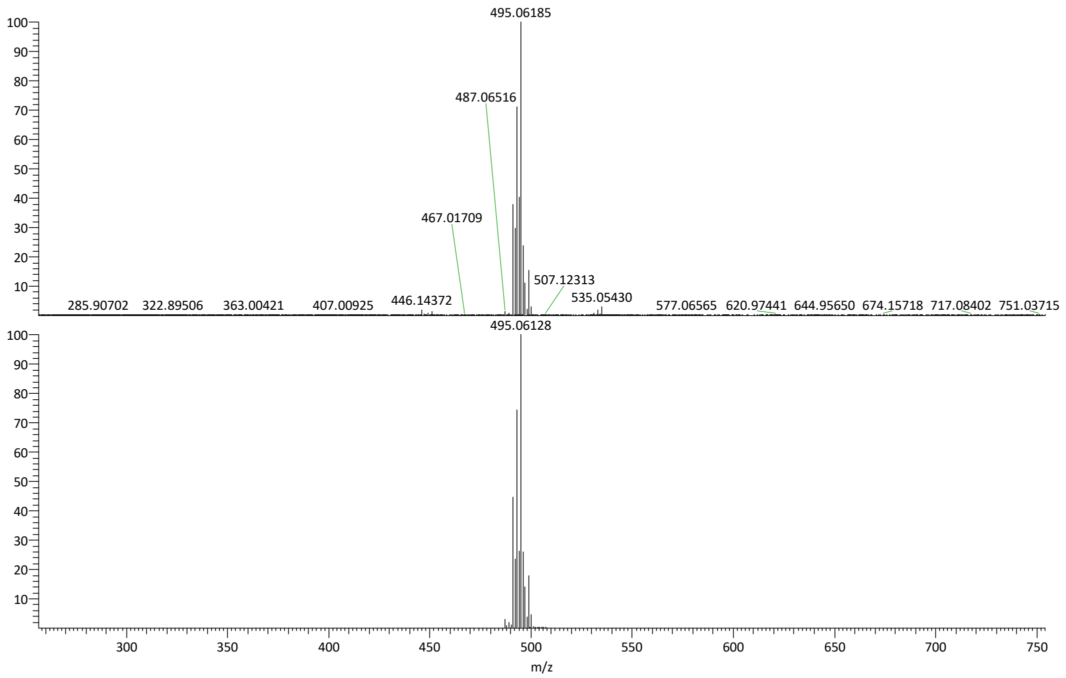

Figure S52. HRMS (ESI+, MeCN) recorded spectrum (*up*) and simulated spectrum (*down*) of compound **1**

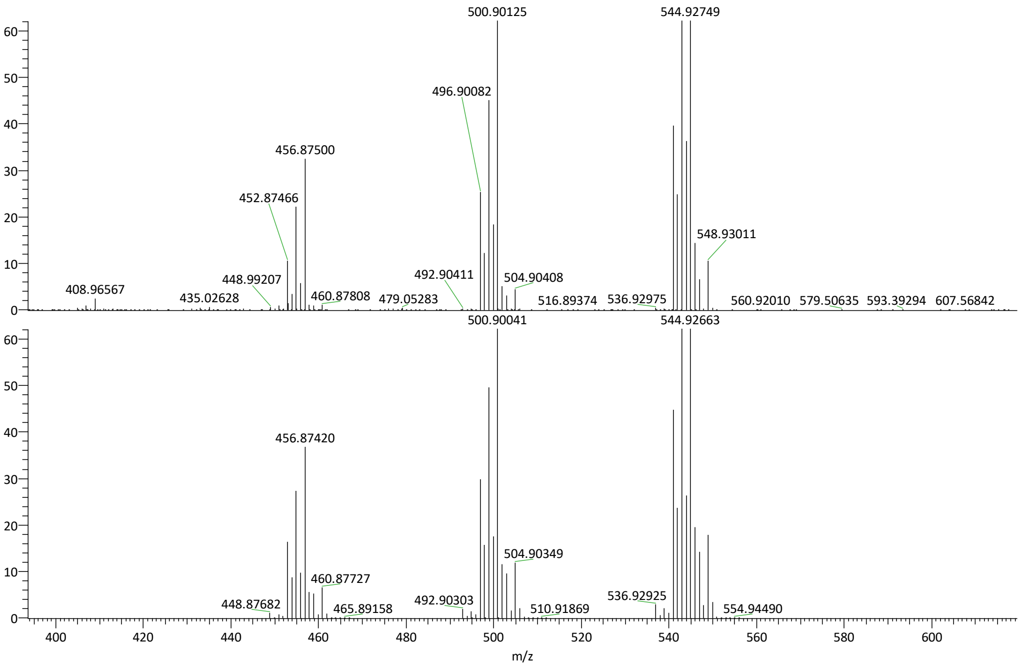

Figure S53. HRMS (APCI+, MeCN) recorded spectrum (*up*) and simulated spectrum (*down*) of compound **2**

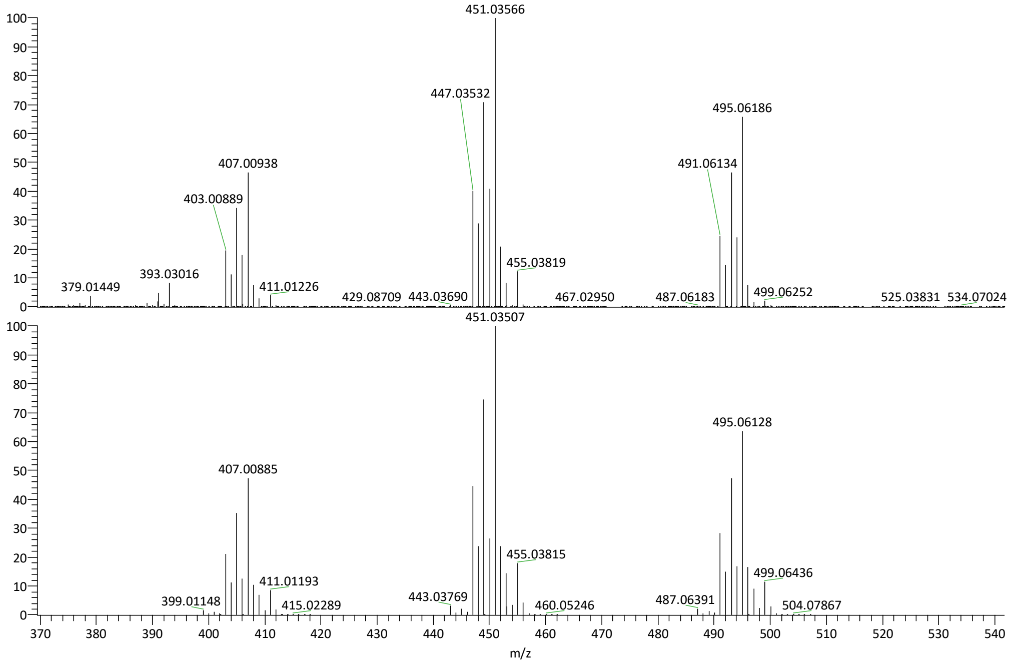

Figure S54. HRMS (APCI+, MeCN) recorded spectrum (*up*) and simulated spectrum (*down*) of compound **3**

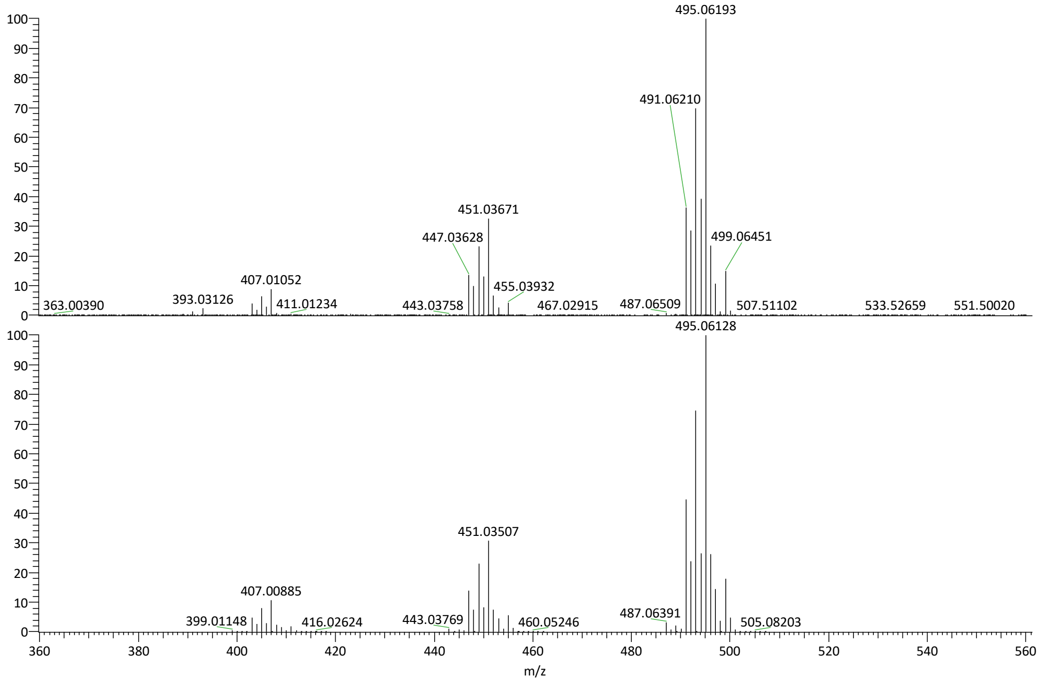

Figure S55. HRMS (APCI+, MeCN) recorded spectrum (*up*) and simulated spectrum (*down*) of compound **5**

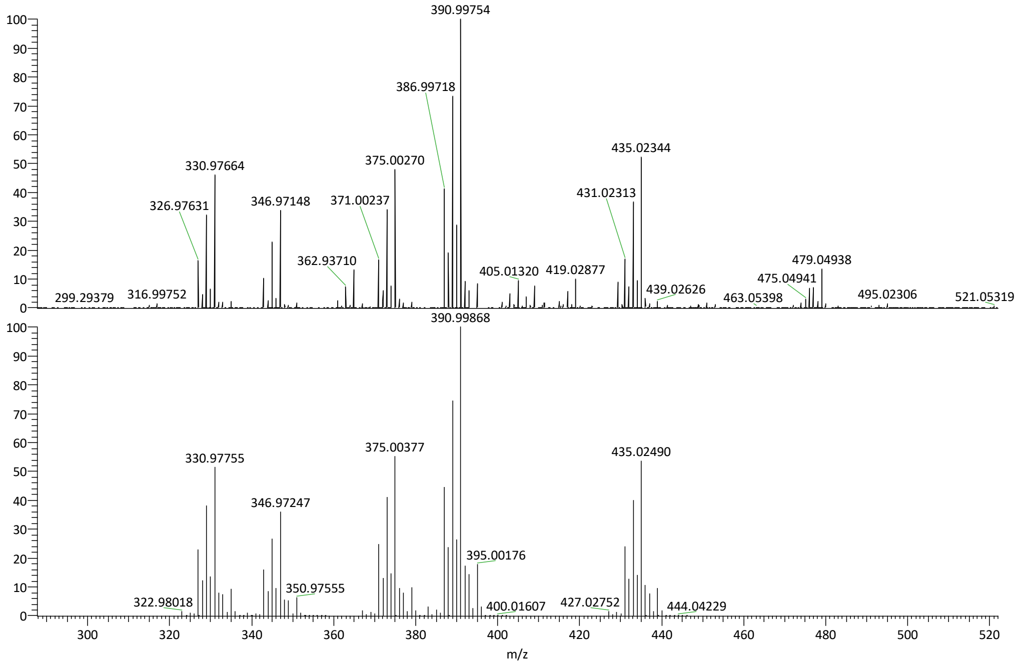

Figure S56. HRMS (APCI+, MeCN) recorded spectrum (*up*) and simulated spectrum (*down*) of compound **8**

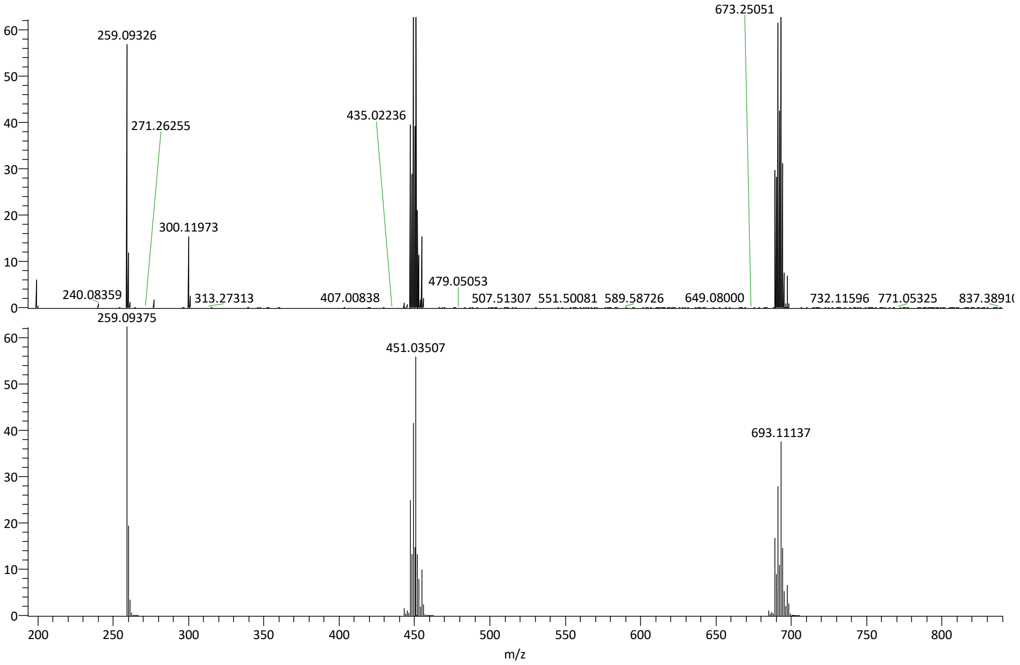

Figure S57. HRMS (APCI+, MeCN) recorded spectrum (*up*) and simulated spectrum (*down*) of compound **10**

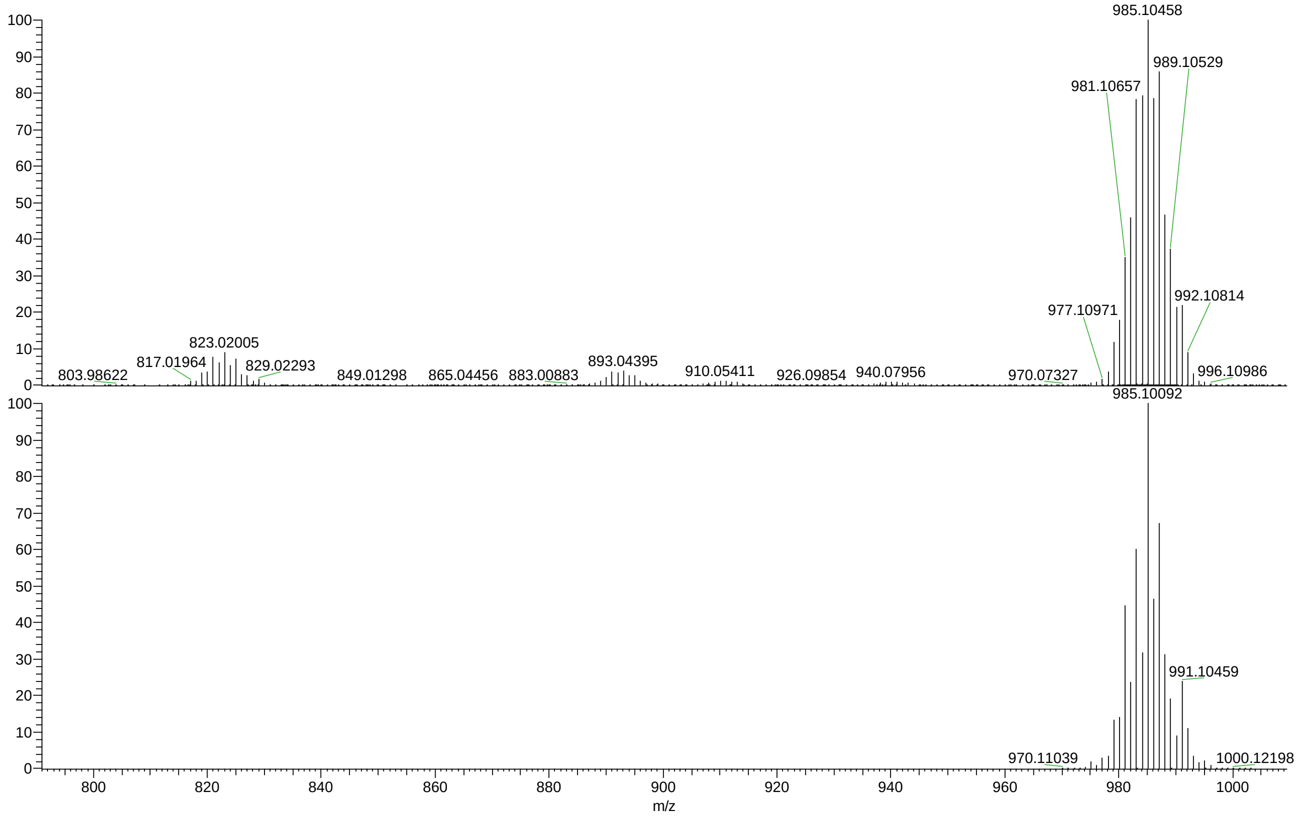

Figure S58. HRMS (APCI+, MeCN) recorded spectrum (*up*) and simulated spectrum (*down*) of compound **12**.

Thermogravimetric analysis (TGA and DTA curves)

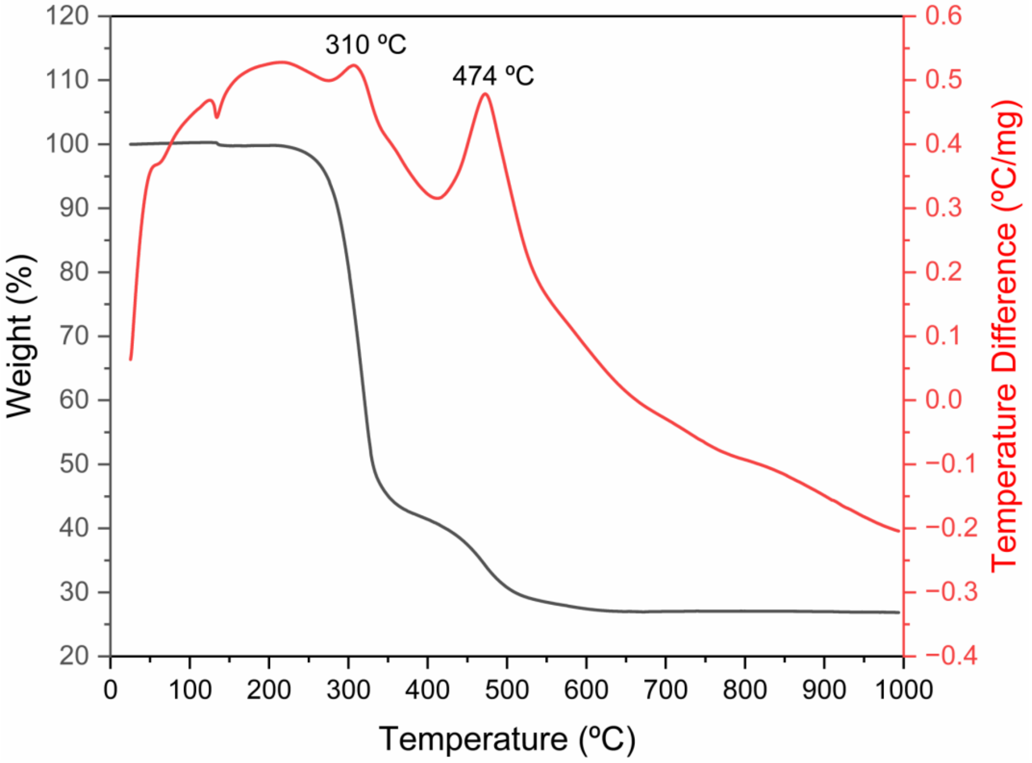

Figure S59. Thermogravimetry (TG- black) and differential thermal analysis (DTA-red) curves of $L_2PhSnOSiPh_3$ (**3**) (exo up).

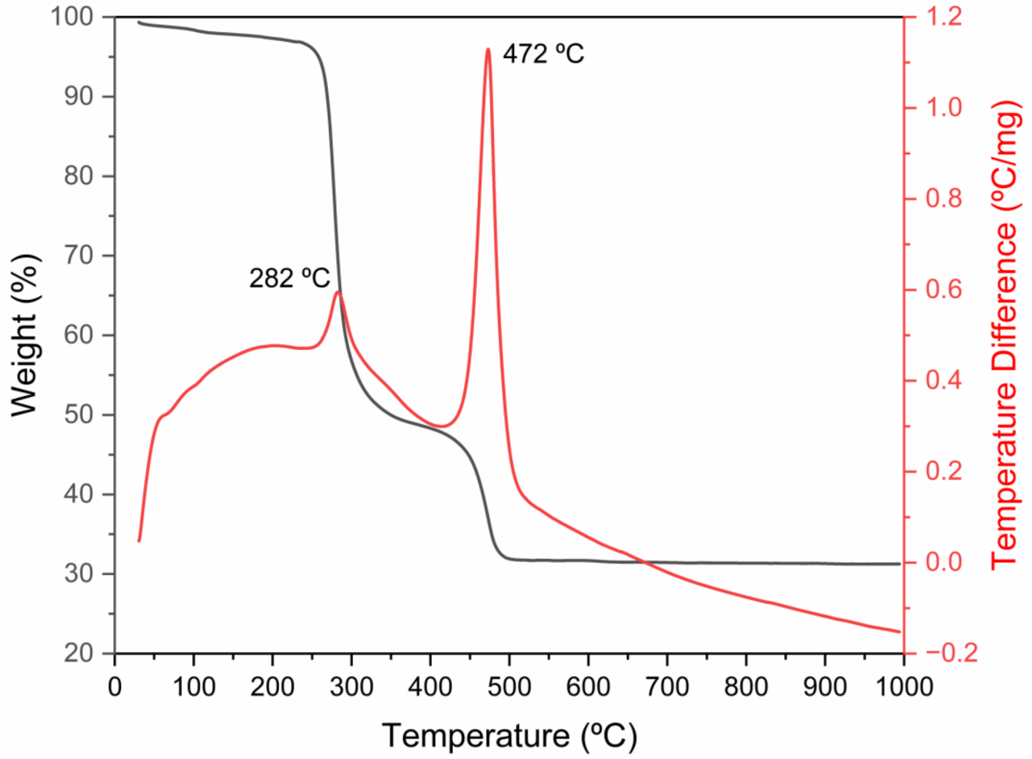

Figure S60. Thermogravimetry (TG- black) and differential thermal analysis (DTA-red) curves of L_2PhSnO^tBu (**4**) (exo up).

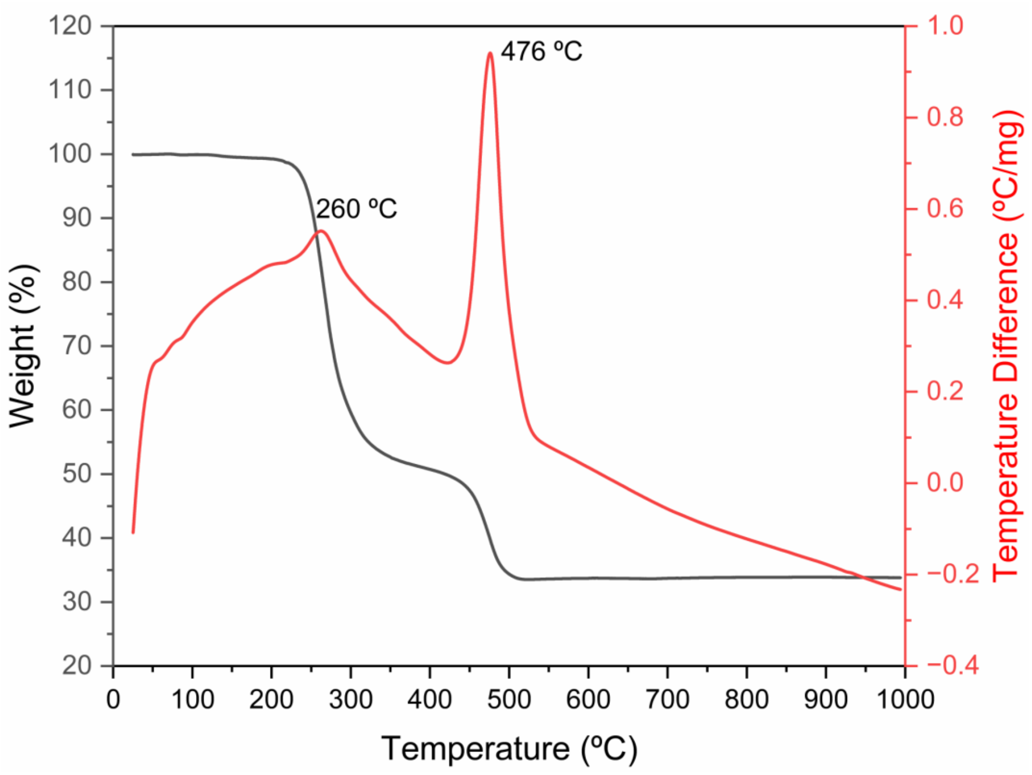

Figure S61. Thermogravimetry (TG- black) and differential thermal analysis (DTA-red) curves of $(L_2SnPh)_2O$ (**5**) (exo up).

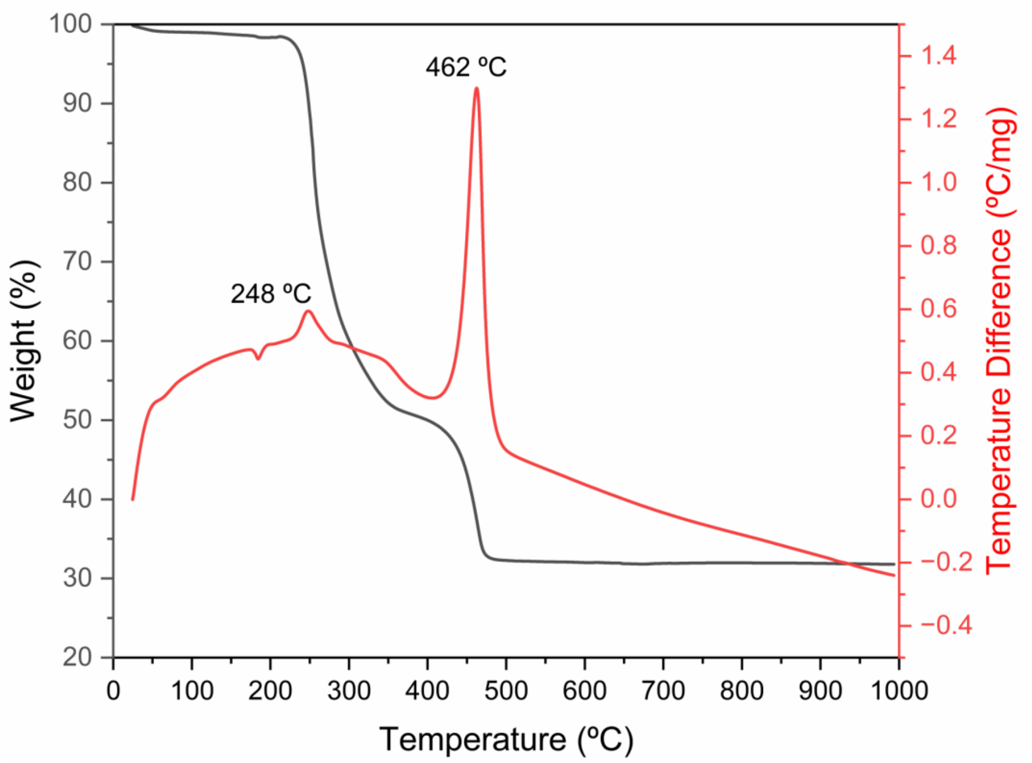

Figure S62. Thermogravimetry (TG- black) and differential thermal analysis (DTA-red) curves of $(L_2SnO)_3$ (**8**) (exo up).

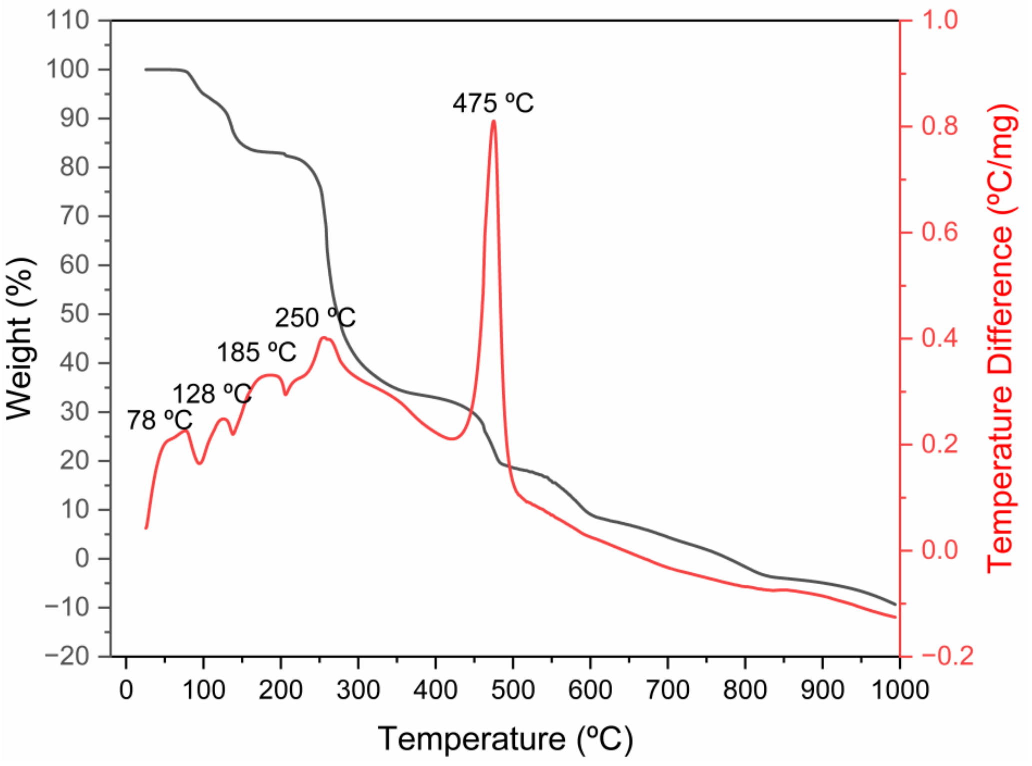

Figure S63. Thermogravimetry (TG- black) and differential thermal analysis (DTA-red) curves of **12** (exo up).

Table S8. TGA data for **3-5**, **8**, **10** and **12**

Compound	Molar mass [g/mol]	Decomposition range [°C]	% Loss observed / calculated	Residual left behind	% Residue observed / calculated
**3**	769.556	550-650	73.1/72.6	$SnO_2.SiO_2$	26.9/27.4
**4**	567.269	420-500	68.6/69.2	2C, SnO_2	31.4/30.8
**5**	1004.307	400-500	66.3/66.8	4C, Sn_2O_3	33.7/33.2
**8**	1299.141	410-500	68.0/68.8	10C, Sn_2O_3	32.0/31.2
**10**	967.852	500-660	78.7/78.2	$SnO_2.SiO_2$	21.3/21.8
**12**	984.036	500-600	90.9/90.2	8C	9.1/9.8

References

[71] G. M. Sheldrick, *Acta Cryst. A*, **2015**, *71*, 3.

Author Contributions

Vlad Penciu performed all synthetic experimental work and took part to analysis of the data. Richard A. Varga performed the X-ray diffraction analysis for all complexes and took part to the review and editing of the manuscript. Liliana Bizo performed the thermogravimetric studies and took part to analysis of the data. Adrian-A. Someșan participated to the design of experiments, analysis of experimental data and writing of the original draft.
